# Regulating Tumor Metabolic Reprogramming with Biomimetic Co‐Delivery of Simvastatin and Kynureninase for Immunotherapy

**DOI:** 10.1002/advs.202508107

**Published:** 2025-12-23

**Authors:** Jiaxin Yin, Shengcai Yang, Zengguang Liu, Songchen Zhao, Siyu Sun, Ziling Liu, Quanshun Li

**Affiliations:** ^1^ Department of Cancer Center The First Hospital of Jilin University Changchun 130012 China; ^2^ Key Laboratory for Molecular Enzymology and Engineering of the Ministry of Education School of Life Sciences Jilin University Changchun 130012 China; ^3^ China‐Singapore Belt and Road Joint Laboratory on Liver Disease Research The First Hospital of Jilin University Changchun 130012 China

**Keywords:** immunotherapy, kynureninase, simvastatin, tumor metabolic reprogramming

## Abstract

The metabolic reprogramming of immunosuppressive tumor microenvironment (ITME) greatly influences the anti‐tumor immunity. Bioinformatic analysis demonstrates that indoleamine 2,3‐dioxygenase 1 and 3‐hydroxy‐3‐methylglutaryl coenzyme A reductase (key enzymes of kynurenine (Kyn) and cholesterol metabolism, respectively), are overexpressed in human colon adenocarcinoma tissues. Herein, biomimetic and pH/ROS dual‐responsive nanoparticles (PTSK@CRM) loaded with simvastatin and kynureninase (KYNase) are prepared to regulate Kyn and cholesterol metabolism, thereby enhancing the immunotherapeutic efficacy of PD‐1 antibody (αPD‐1). The monodisperse spherical PTSK@CRM is stable at pH 7.4, while it can release simvastatin and KYNase under low pH and high H_2_O_2_ concentration. PTSK@CRM achieves excellently homologous tumor targeting to CT26 cells and induces cell apoptosis more effectively than PTSK. Moreover, PTSK@CRM significantly reduces the contents of Kyn and cholesterol and decreases the activation of the Kyn‐AhR pathway in tumor metabolism. In vivo experiments show that PTSK@CRM possesses a favorable tumor‐targeting ability to effectively suppress tumor growth and increase the infiltration of immune cells, including CD8^+^ T cells, CD4^+^ T cells, M1‐like macrophages, and mature dendritic cells. Further, PTSK@CRM reduces the infiltration of immunosuppressive cells, thereby reversing ITME to improve the therapeutic efficacy of αPD‐1. Overall, this immune‐metabolic therapeutic strategy provides a potential route for remodeling ITME to enhance tumor immunotherapy.

## Graphical abstract

After the intravenous injection of biomimetic and pH/ROS‐responsive PTSK@CRM, the nanoparticles can be accumulated in tumors and release Sim and KYNase to inhibit the tumor growth, regulate the metabolism of cholesterol and Kyn, and reverse the immunosuppressive tumor microenvironment. The combined treatment of PTSK@CRM and αPD‐1 further improves the immunotherapeutic efficacy on CT26 tumor‐bearing BALB/c mice.

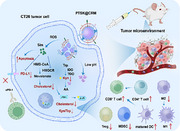

## 1 Introduction

Colorectal cancer (CRC) ranked as the third most prevalent malignant tumor worldwide in 2022 with over 1.9 million new cases and more than 0.9 million associated deaths.^[^ ^1^ ^]^ Immune checkpoint blockades (ICBs) have achieved breakthrough strides in treating various solid tumors including CRC over the past decade.^[^ ^2^ ^]^ ICBs represented by programmed death‐1 antibody (αPD‐1) and programmed death‐ligand 1 (PD‐L1) antibody have been demonstrated to markedly enhance the efficacy and prognosis of patients with CRC.^[^ ^3^ ^]^ However, over half of advanced CRC patients experience resistance against immunotherapy owing to the immunosuppressive tumor microenvironment (ITME).^[^ ^4^, ^5^ ^]^ Therefore, reversing the ITME and reinvigorating the anti‐tumor immunity of hosts will be a potential strategy for CRC therapy.

Aberrant metabolism contributes to the progression of ITME and has attracted considerable attention in recent years.^[^ ^6^ ^]^ Metabolic reprogramming has emerged as a hallmark of malignancy, which refers to the pivotal metabolic alterations to meet the demands of uncontrolled cell proliferation in the tumor microenvironment (TME). Predictably, this gives rise to nutrient depletion, hypoxia, acidity, and metabolites’ accumulation.^[^ ^7^ ^]^ Finally, metabolic dysregulation will lead to drug resistance, immunosuppression, and poor clinical outcomes.^[^ ^8^ ^]^ Various metabolic phenotypes and pathways are involved in the reprogramming process, including glycolysis characterized by pioneered Warburg's effect, and metabolism of amino acids and lipids.^[^ ^9^, ^10^ ^]^

In the metabolism of amino acids, the tryptophan (Trp) and kynurenine (Kyn) pathway stands out in reprogramming the TME.^[^ ^11^ ^]^ Clinically, the overexpression of indoleamine 2,3‐dioxygenase (IDO) and tryptophan 2,3‐dioxygenase (TDO) in tumors results in the depletion of Trp alongside the accumulation of Kyn, followed by the activation of aryl hydrocarbon receptor (AhR) as a transcription factor.^[^ ^12^ ^]^ AhR signaling pathway not only promotes tumor growth but also facilitates immune evasion. The activation of AhR in dendritic cells (DCs) suppresses the expression of MHC‐II and the production of pro‐inflammatory cytokines, thereby increasing the IDO1 expression to improve the Kyn level. In macrophages, the AhR activation drives an immunosuppressive M2‐like phenotype. Further, AhR enhances the secretion of anti‐inflammatory cytokines by Th17 and regulatory T cells (Tregs), synergizing with Kyn to promote the Treg expansion and functional activity.^[^ ^13^, ^14^ ^]^ In conclusion, Kyn exerts multifarious immunosuppressive effects by recruiting Tregs and tumor‐associated macrophages (TAMs) into ITME and inhibits the anti‐tumor activity of effector T cells and DCs.^[^ ^11^, ^15^ ^]^ To date, the anti‐tumor efficacy of IDO1 inhibitors has been successfully proved, such as NLG919 and Indoximod.^[^ ^16^ ^]^ However, these small molecular inhibitors are prone to induce drug‐resistant mutations and do not sufficiently prevent the production of Kyn, as TDO could also catalyze the conversion of Trp to Kyn. Notably, kynureninase (*KYNU*, KYNase), a therapeutic enzyme that specifically hydrolyzes Kyn into anthranilic acid (AA) and alanine, shows the potential to overcome these limitations.^[^ ^17^ ^]^ Previous studies have shown that the KYNase‐mediated elimination of Kyn could reverse the ITME, block the immune escape, and elicit the host immune system.^[^ ^17^, ^18^ ^]^ While KYNase from *Pseudomonas fluorescens* (*Pf*KYNase) preferentially hydrolyzes Kyn compared to *Homo sapiens* KYNase (*Hs*KYNase), concerns related to immunogenicity limited its clinical application.^[^ ^17^ ^]^ An evolved *Hs*KYNase variant (*Hs*KYNase_95) was selected as it possessed high catalytic activity for Kyn, low immunogenicity risk, and superior serum stability.^[^ ^19^ ^]^ However, the in vivo delivery of therapeutic enzymes faces enormous challenges, as they are susceptible to be degraded during the in vivo process and unable to penetrate into cells due to their high molecular weight.^[^ ^18^ ^]^ Thus, it is a great challenge to develop nano‐delivery systems for the enzyme‐based immunotherapy.

Lipid metabolism also plays a predominant role in the synthesis of biological membranes, and signaling molecules related to the proliferation, invasion, and migration of cancer cells.^[^ ^20^ ^]^ The cholesterol and its metabolites synthesized de novo via the mevalonate pathway not only accelerate the tumor growth but also reprogram the immune cells (e.g., T cells and macrophages).^[^ ^21^, ^22^, ^23^ ^]^ Notably, 3‐hydroxy‐3‐methylglutaryl coenzyme A reductase (HMGCR) is the critical rate‐limiting enzyme in the conversion of 3‐hydroxy‐3‐methylglutaryl coenzyme A (HMG‐CoA) into mevalonate.^[^ ^24^, ^25^ ^]^ Previous reports have shown that statins, known as the HMGCR inhibitor, could improve the survival outcome in patients across different kinds of tumors.^[^ ^26^ ^]^ Specifically, growing evidence suggested that statins, including simvastatin (Sim), atorvastatin, and lovastatin, could significantly reduce the cholesterol level and induce cell apoptosis.^[^ ^27^ ^]^ Moreover, Sim could inhibit the PD‐L1 expression through suppressing the lncRNA SNHG29‐mediated YAP signaling axis.^[^ ^28^ ^]^ Taken together, the manipulation of cholesterol metabolism possesses the potential to reinvigorate the anti‐tumor immunity. This strategy may lead to the activation of T helper cells and cytotoxic T cells, the repolarization of TAMs from M2 to M1 phenotype, and the regulation of dysfunctional DCs.^[^ ^24^, ^29^, ^30^, ^31^, ^32^ ^]^ Thus, it will be of great significance to combine the regulation of cholesterol and Kyn metabolism in achieving effective cancer immunotherapy. Additionally, the low solubility and rapid clearance of Sim result in an undesirable bioavailability profile, thereby impeding its clinical application.^[^ ^33^, ^34^ ^]^ Hence, it is essential to develop a suitable delivery platform to enhance the pharmacokinetic profiles of statins.

**Scheme 1:**
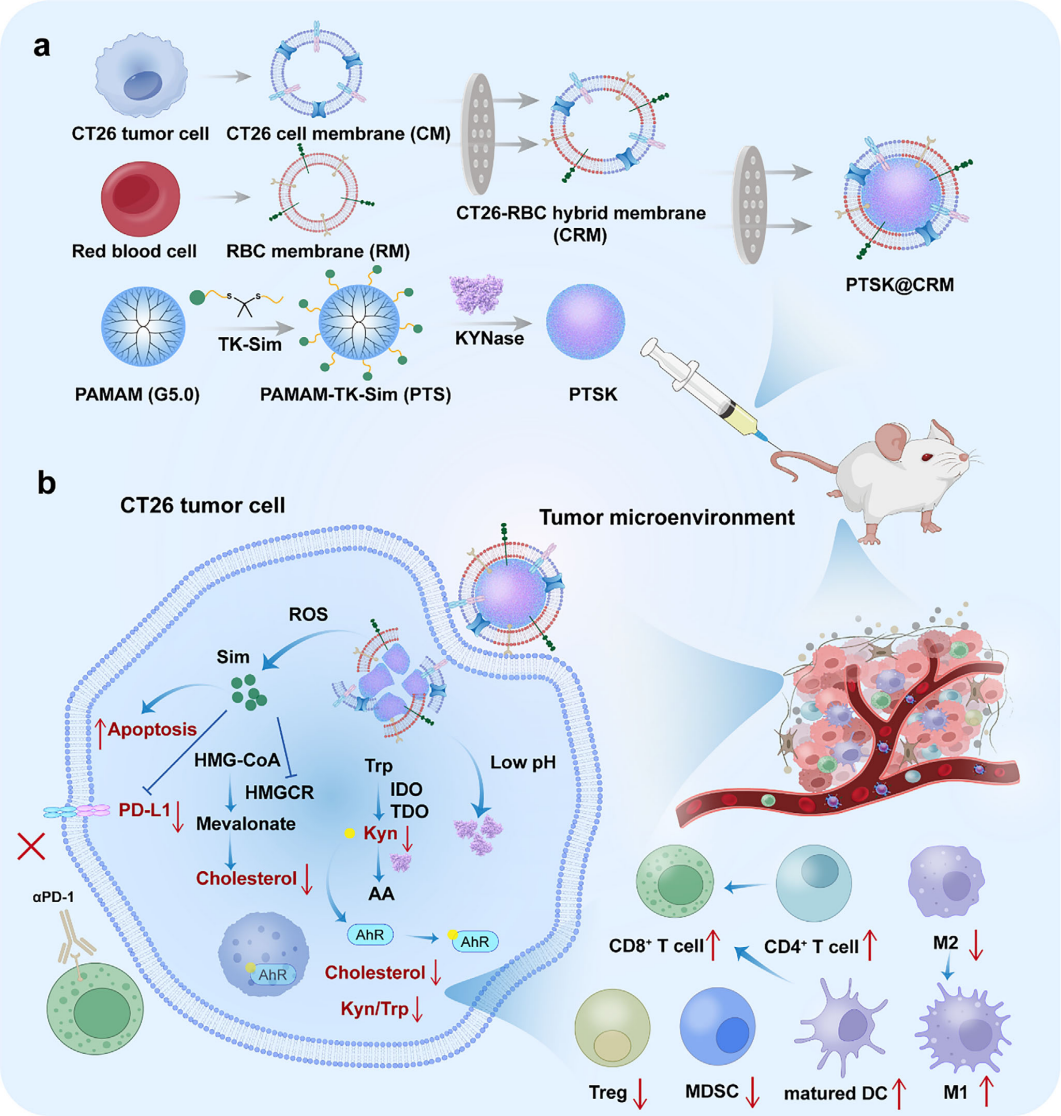
Construction of PTSK@CRM and its immunotherapeutic mechanism. a) Preparation process of biomimetic and pH/ROS dual‐responsive PTSK@CRM nanoparticles. b) Through the intravenous administration, the PTSK@CRM nanoparticles can accumulate in tumors via the membrane‐mediated targeting specificity, and release Sim and KYNase to inhibit the tumor growth, regulate cholesterol and Kyn metabolism, and reverse the ITME. Moreover, the combination of PTSK@CRM and αPD‐1 remarkably improves the immunotherapeutic efficacy on CT26 tumor‐bearing BALB/c mice.

In this work, a novel paradigm was established for cancer immunotherapy through dual modulation of Kyn and cholesterol metabolic pathways. A biomimetic and pH/ROS dual‐responsive nanoparticle (denoted by PTSK@CRM) was prepared, which achieved the co‐delivery of Sim and KYNase to regulate the tumor metabolic reprogramming and enhance the immunotherapeutic efficacy (**Scheme** **1**). Sim was covalently conjugated to polyamidoamine (PAMAM, G5.0) via a ROS‐cleavable thioketal (TK) linker to prepare PAMAM‐TK‐Sim (PTS). This conjugation enhanced the Sim's water solubility and enabled its responsive release in ROS‐rich tumor microenvironment. KYNase was subsequently self‐assembled with PTS through electrostatic and *π–π* stacking interactions to obtain KYNase‐loaded PTS nanoparticles (denoted by PTSK). Then, PTSK was camouflaged with a biomimetic hybrid membrane (CRM) to achieve tumor targeting and in vivo long circulation ability. The particle size, zeta potential, and pH/ROS responsive properties of PTSK@CRM were investigated. The in vitro targeting and anti‐tumor activity were studied against CT26 cells, a well‐established murine colon carcinoma model exhibiting conserved CRC biomarkers and dysregulated metabolic pathways. Then, the in vivo biodistribution, anti‐tumor efficacy, and immune cell infiltration of PTSK@CRM were evaluated on subcutaneous CT26 tumor‐bearing BALB/c mice. The therapeutic efficacy and metabolic reprogramming of its combination with αPD‐1 were further investigated. Finally, the anti‐tumor efficacy of PTSK@CRM was explored in CT26 lung metastasis mice and subcutaneous MC38 tumor‐bearing mice, respectively. Collectively, the combination of PTSK@CRM with the first‐line immunotherapy is expected to achieve remarkable therapeutic efficacy, which enables dual metabolic immunotherapy as a breakthrough strategy.

## 2 Results and Discussion

### 2.1 Expression and Pathway Analysis of *KYNU* and *HMGCR* in Colon Cancer Tissues

**Figure 1:**
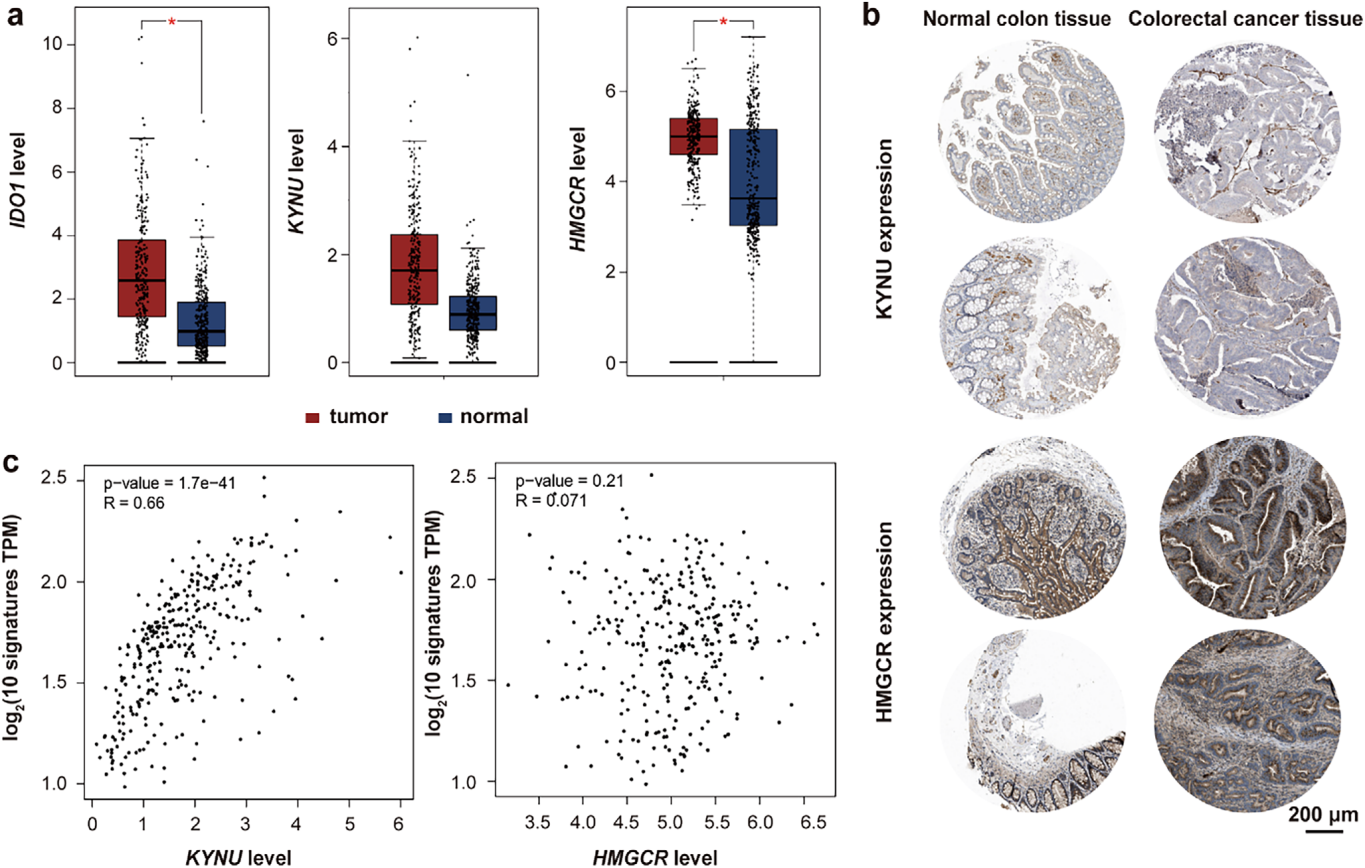
Expression analysis of *KYNU* and *HMGCR* from human colon tissues. a) The mRNA levels of *IDO1*, *KYNU*, and *HMGCR* in COAD patient tissues (*n* = 275) and normal tissues (*n* = 349) from the GEPIA database (unpaired and two‐tailed *t*‐test, *P < 0.05). b) The protein expression of KYNU and HMGCR in colorectal cancer and normal colon tissues from the HPA database (scale bar = 200 µm). c) The Spearman correlation analysis between *KYNU* or *HMGCR* mRNA levels and signature levels of immune cells (effector T cells and Th cells) in COAD tissues from the GEPIA database. Data were pre‐processed by normalization.

To investigate the role of Kyn and cholesterol metabolism in CRC, the *IDO1*, *KYNU*, and *HMGCR* expression of CRC patients was studied based on the Cancer Genome Atlas (TCGA) database. The differential mRNA levels were analyzed between 275 colon adenocarcinoma (COAD) tissue samples and 349 normal colon tissue samples using the Gene Expression Profiling Interactive Analysis (GEPIA) database. As shown in **Figure** **1**a, the *IDO1* and *HMGCR* mRNA levels of COAD tissues were significantly higher than those of normal colon tissues, and no significant differences were observed in the *KYNU* mRNA level. Meanwhile, the protein expression of HMGCR in COAD tissues was stronger than that of normal colon tissue through the Human Protein Atlas (HPA) database (Figure 1b; Figure S1a, Supporting Information). The KYNU expression was similar between these two groups, while the expression level of IDO1 in colorectal cancer tissues was higher than that in normal colon tissues (Figure S1b,c, Supporting Information). Additionally, the Kyoto encyclopedia of genes and genomes (KEGG) analysis was employed to evaluate the correlation of *KYNU* (or *HMGCR*) expression and pathway enrichment. The expression of *KYNU* was positively correlated to the differentiation of Th cells, and the Toll‐like receptor and NF‐κB signaling pathways (Figure S2a, Supporting Information), while *HMGCR* expression was associated with the carbon and pyruvate metabolism (Figure S2b, Supporting Information). The correlation analysis between *KYNU* (or *HMGCR*) and gene signatures of immune cells (effector T cells and Th cells) in COAD tissues was employed by the GEPIA databases. As illustrated in Figure 1c and Figure S1d (Supporting Information), the level of *KYNU* was positively correlated to the level of T cell signatures, whereas the *HMGCR* level had no significance. Besides, the correlation between *KYNU* (or *HMGCR)* expression and immune cell infiltration was explored by the Tumor Immune Estimation Resource 2.0 (TIMER 2.0) database. The *KYNU* expression was positively correlated with the increased infiltration of CD8^+^ T cells, CD4^+^ T cells, macrophages, neutrophils, and DCs, while the *HMGCR* expression was only positively correlated with the infiltration of myeloid‐derived suppressor cells (MDSCs) (Figure S2c,d, Supporting Information). All these results revealed that the aberrant *IDO1* and *HMGCR* expression in colon cancer patients may result in the accumulation of immunosuppressive Kyn and cholesterol in TME. Thus, the depletion of Kyn and cholesterol could probably play a vital role in the activation of anti‐tumor immune response.

### 2.2 Preparation and Characterization of PTSK@CRM Nanoparticles

**Figure 2:**
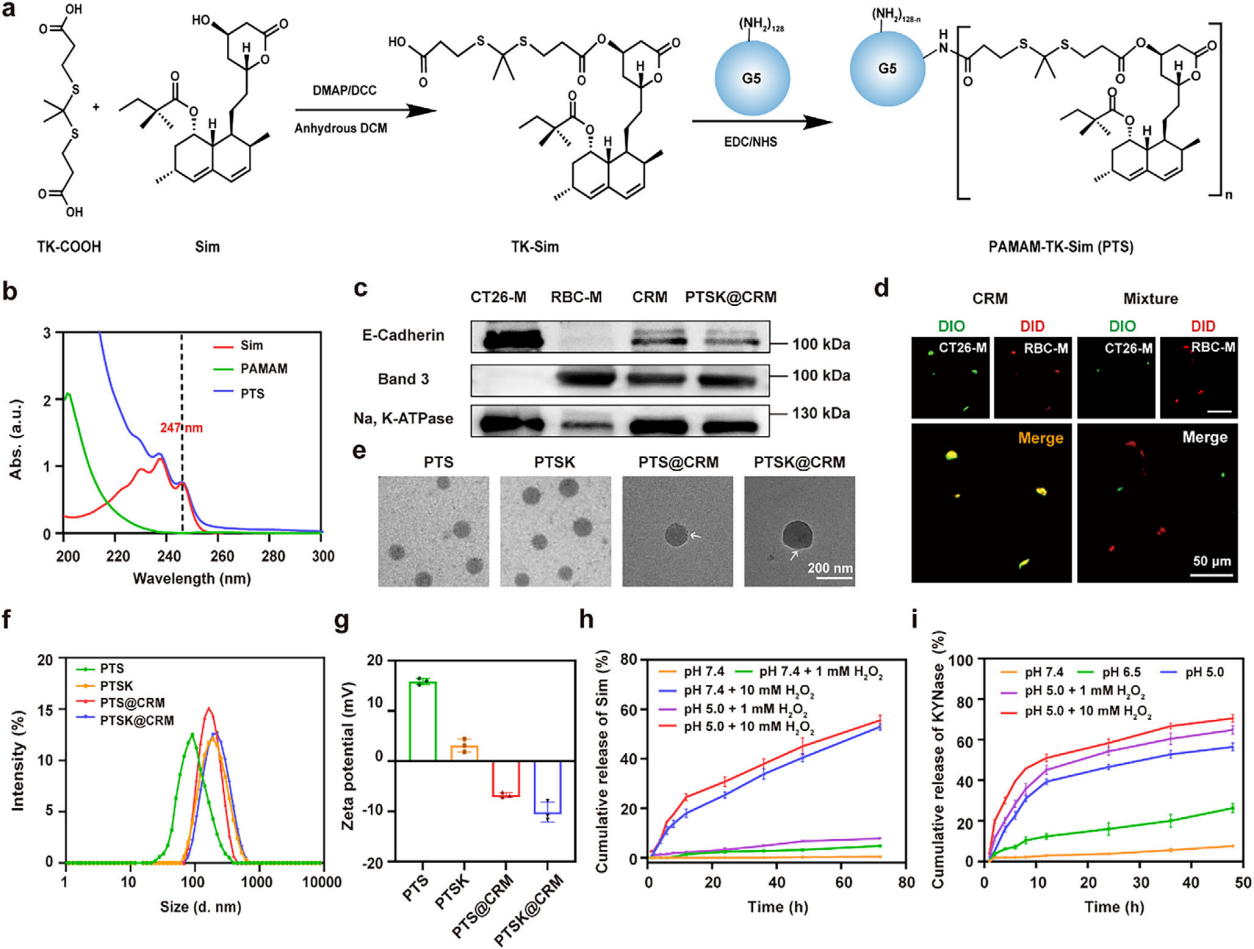
Preparation and characterization of PTSK@CRM. a) The synthetic route of PAMAM‐TK‐Sim (PTS). b) The UV–vis spectra of Sim, PAMAM, and PTS. c) Western blotting analysis of characteristic markers in CT26‐M, RBC‐M, CRM, and PTSK@CRM. d) Membrane fusion of hybrid membranes CRM was detected by a fluorescence microscope. CT26‐M and RBC‐M were labeled with DiO (green) and DiD (red), respectively, scale bar = 50 µm. e) TEM images of PTS, PTSK, PTS@CRM and PTSK@CRM, scale bar = 200 nm. White arrows represent the structure of the camouflaged membrane. f) Hydrodynamic diameter distribution and g) Zeta potential of PTS, PTSK, PTS@CRM, and PTSK@CRM. h) The cumulative release of Sim from the PTS at different buffers. i) The cumulative release of KYNase from PTSK at different buffers. Data were presented as the mean value ± standard deviation (SD, n = 3).

First, TK‐Sim was synthesized through the esterification reaction of 3,3′‐(Propane‐2,2‐diylbis(sulfanediyl)) dipropionic acid (TK‐COOH) and Sim (**Figure** **2**a), and there was an obvious characteristic proton peak (**peak c** (‐C*H*_3_)) in the ^1^H NMR spectra (Figure S3a, Supporting Information). In the ESI‐Q‐TOF mass spectra, the ions ([TK‐Sim ‐ H]^−^, [TK‐Sim], [TK‐Sim + H]^+^ at *m/z* 651.6, 652.7, and 653.7) were also observed, indicating the successful synthesis of TK‐Sim (Figure S3b, Supporting Information). Next, the PAMAM was covalently modified with TK‐Sim to prepare PAMAM‐TK‐Sim (PTS), and the characteristic peaks (peaks a and b) could be observed in ^1^H NMR spectra (Figure S3c, Supporting Information). In addition, there was an obvious absorbance peak of Sim at 247 nm in the UV–vis spectra of PTS, which further confirmed the successful preparation of PTS (Figure 2b). The encapsulation efficiency (EE) and drug loading content (DLC) of Sim were measured to be 72.8 ± 0.7% and 10.3 ± 0.5%, respectively (Table S1, Supporting Information). KYNase was expressed and purified according to the previous report,^[^ ^19^ ^]^ and the kinetic parameter *k*_cat_/*K*_m_ against Kyn was calculated to be 1.11 ± 0.22 × 10^4^ m ^−1 ^s^−1^ (Figure S4, Supporting Information). Afterward, KYNase was encapsulated with PTS at a mass ratio of 1:3.5 to obtain KYNase‐loaded PTS nanoparticles (denoted as PTSK). The EE and DLC of KYNase were determined to be 83.4 ± 0.8% and 21 ± 1.3%, respectively (Table S1, Supporting Information).

To achieve in vivo homologous targeting and long circulation of nanoparticles, the CT26 cell membrane (CM) and red blood cell membrane (RBC‐M, RM) were extracted and extruded to prepare the CT26‐RBC biomimetic hybrid membrane (CRM). The SDS‐PAGE analysis indicated that the CRM retained the characteristic proteins inherited from CM and RM (Figure S5, Supporting Information). In addition, the expression of E‐cadherin (the cell adhesion molecule, specific protein marker of CM), Band 3 (the transmembrane protein, specific protein marker of RM), and Na, K‐ATPase (internal reference) was detected by Western blotting (Figure 2c). Clearly, both E‐cadherin and Band 3 proteins were presented in CRM and PTSK@CRM samples. These results were consistent with previous reports, as the coating with a biomimetic hybrid membrane could retain the native membrane proteins.^[^ ^35^ ^]^ To further observe the membrane fusion, CM and RM were labeled with DIO and DID, respectively. Compared to the direct mixture, the hybrid membrane CRM through sonication and extrusion showed yellow fluorescence under a fluorescence microscope, as the CM and RM were under fusion (Figure 2d).

PTSK@CRM was also prepared from the mixture of PTSK and CRM through sonication and extrusion. As shown in Figure S5 (Supporting Information), both PTS@CRM and PTSK@CRM exhibited similar protein profiles of CRM, demonstrating that the characteristic protein markers have been transferred to the PTS@CRM and PTSK@CRM nanoparticles. Notably, there was an obvious enrichment of KYNase on PTSK@CRM in comparison to PTS@CRM, which further suggested the successful loading of KYNase. Transmission electron microscopy (TEM) images showed that PTS and PTSK could be self‐assembled into a uniform and monodisperse spherical structure (Figure 2e). Meanwhile, PTS@CRM and PTSK@CRM were coated with a thin translucent film as indicated by the arrow, demonstrating that the nanoparticles were successfully coated with the camouflaged membrane. The hydrodynamic diameters of PTSK and PTSK@CRM were measured to be 168.6 ± 7.6 and 181.3 ± 2 nm, respectively, which was consistent with TEM results (Figure 2f). The zeta potential values of PTSK and PTSK@CRM were 3.11 ± 1.1 and −10.2 ± 1.6 mV, respectively, as CRM was negatively charged (Figure 2g, Table S1, Supporting Information). The particle size and zeta potential of PTSK@CRM did not change significantly in PBS, 10% fetal bovine serum (FBS)‐containing or 35% human serum (HS)‐containing RPMI 1640 medium for 5 days, indicating its favorable stability under these conditions (Figure S6, Supporting Information). However, the hydrodynamic diameter of PTSK@CRM ovbiously changed under low pH and high H_2_O_2_ concentration, meaning that it was unstable under these conditions (Figure S7, Supporting Information).

To study the ROS‐sensitivity of PTS, the nanoparticles were incubated in PBS containing 1 mm or 10 mm H_2_O_2_ for 24 h. Nearly negligible drug release could be detected by high performance liquid chromatography (HPLC) in the absence of H_2_O_2_, and there was an improved release of Sim upon the addition of H_2_O_2_ (Figures S8,S9, Supporting Information). The cumulative release of Sim in pH 5 + 10 mm H_2_O_2_ reached 55.5 ± 1.6% within 72 h, while the value in pH 5 + 1 mm H_2_O_2_ was only 7.8 ± 0.3% (Figure 2h; Figure S10, Supporting Information). This ROS‐responsive Sim release was triggered by in the introduction of ROS‐cleavable linker, which was consistent with the previous report.^[^ ^36^ ^]^ Meanwhile, the cumulative release of KYNase in PTSK was measured by the BCA protein assay kit. As shown in Figure 2i, the release was significantly accelerated in the buffers with pH of 5 or pH 6.5 in comparison to pH of 7.4. The cumulative release of KYNase in PTSK was measured to be 26.3 ± 1.8% and 56.4 ± 1.6% after the incubation under pH of 6.5 and 5, respectively. As PTSK was prepared through the electrostatic and *π–π* stacking interactions between PAMAM and KYNase, PTSK could release KYNase under acid conditions owing to the intrinsic pH‐sensitivity of PAMAM.

### 2.3 In Vitro Homologous Targeting and Cellular Uptake Ability

**Figure 3:**
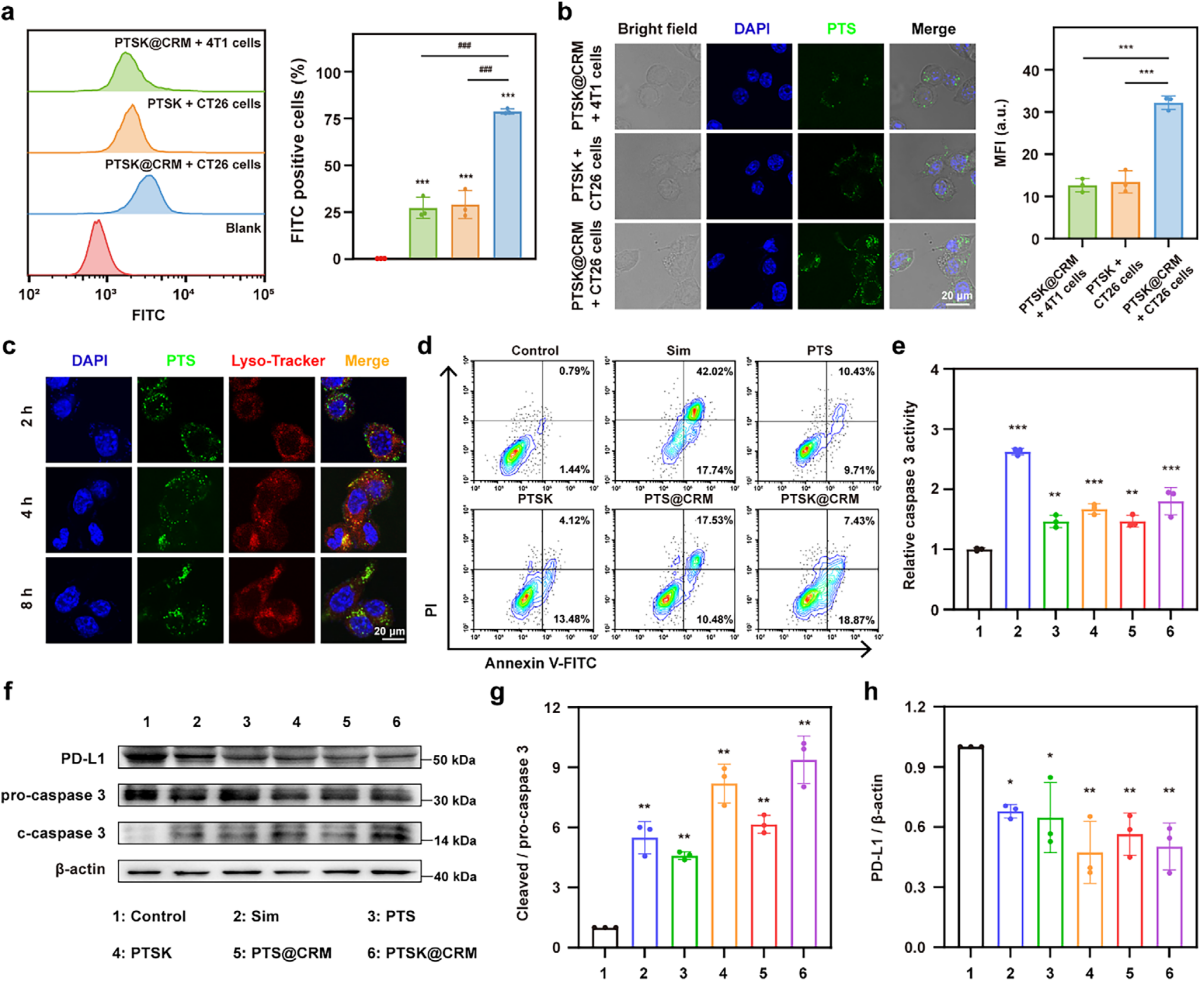
In vitro cellular uptake and anti‐tumor mechanism of PTSK@CRM. a) Flow cytometry and quantitative analysis of CT26 and 4T1 cells after the incubation with FITC‐PTSK or FITC‐PTSK@CRM for 4 h (100 µg mL^−1^ on basis of PTS, *n* = 3, ***P < 0.001 vs Control group, ^###^ *P* < 0.001 vs PTSK@CRM + CT26 group). b) CLSM images and mean fluorescence intensity (MFI) analysis of CT26 and 4T1 cells after the incubation with FITC‐PTSK or FITC‐PTSK@CRM for 4 h (100 µg mL^−1^ on the basis of PTS, green: FITC‐PTS, blue: DAPI‐nucleus, scale bar = 20 µm, *n* = 3). c) CLSM images for the lysosomal escape of PTSK@CRM. (green: FITC‐PTS, blue: DAPI‐nucleus, red: Lyso‐Tracker Red, scale bar = 20 µm). d) Representative flow cytometry plots of Annexin V‐FITC and PI double staining for CT26 cells after different treatments for 48 h (5 µg mL^−1^ on the basis of Sim). e) Relative activity of caspase 3 in CT26 cells after different treatments for 48 h (5 µg mL^−1^ on the basis of Sim, *n* = 3). f) Western blotting and semiquantitative analysis of g) pro‐caspase 3, cleaved‐caspase 3 (c‐caspase 3), and h) PD‐L1 protein after different treatments for 24 h (10 µg mL^−1^ on the basis of Sim, *n* = 3). Data were presented as mean value ± SD and analyzed via one‐way ANOVA analysis (*P < 0.05, **P < 0.01, ***P < 0.001).

To investigate the homologous targeting of PTSK@CRM, FITC‐labeled PTSK and PTSK@CRM were incubated with NIH/3T3, 4T1, and CT26 cells. Flow cytometric analysis demonstrated that CT26 cells exhibited a threefold higher percentage of FITC‐positive cells than 4T1 cells after incubation with PTSK@CRM for 4 h (**Figure** **3**a). The percentage of FITC‐positive NIH/3T3 cells was significantly lower than that of CT26 cells under the same incubation conditions (Figure S11a,b, Supporting Information). Meanwhile, the percentage of FITC‐positive cells in the PTSK@CRM + CT26 group has been significantly elevated in comparison to the PTSK + CT26 group. Similarly, confocal laser scanning microscopy (CLSM) analysis indicated that the strongest mean fluorescence intensity (MFI) could be detected in the PTSK@CRM + CT26 group (Figure 3b; Figure S11c, Supporting Information), suggesting the membrane‐mediated targeting specificity and self‐recognition affinity of PTSK@CRM to homologous CT26 cells. Further, it was concluded that the incorporation of RBC membrane did not affect the tumor‐targeting ability after assessing the targeting efficiency of PTSK coated with RBC membrane (PTSK@RM), PTSK coated with CT26 cell membrane (PTSK@CM), and PTSK@CRM in CT26 cells (Figure S12, Supporting Information).

To further determine the cellular uptake and endosomal escape of PTSK@CRM, PTSK@CRM (PTS labeled by FITC) was incubated with CT26 cells for 2, 4, or 8 h, in which lysosomes and nucleus were stained with Lyso‐Tracker (red) and DAPI (blue), respectively. As shown in Figure 3c, there was obviously green fluorescence in the cytoplasm, and the intensity was improved with prolonged incubation time. Interestingly, PTSK@CRM (green fluorescence) was co‐localized with lysosomes (red fluorescence) after the incubation for 4 h to yield yellow fluorescence (Figure S13, Supporting Information). With the extension of incubation time to 8 h, the intensity of yellow fluorescence significantly decreased, and the vivid green color signal was clearly observed, indicating that PTSK@CRM was able to escape from lysosomes. These CLSM images suggested that PTSK@CRM was first entrapped in lysosomes and then rapidly escaped from lysosomes. Similarly, the lysosomal escape ability of FITC‐KYNase, FITC‐PTSK, and FITC‐PTSK@CRM were compared in CT26 cells (Figure S14, Supporting Information). The FITC‐KYNase group showed minimal cellular internalization, while PTSK and PTSK@CRM exhibited lysosomal co‐localization at 4 h and lysosomal escape ability after 8 h. Considering the “proton sponge” hypothesis of PAMAM,^[^ ^37^ ^]^ the polymer's buffering capacity might mediate the infiltrative swelling and rupture of lysosomes. This mechanism facilitated the endosomal escape of PTSK@CRM, thereby achieving favorable therapeutic efficacy. Taken together, PTSK@CRM could be internalized by homologous tumor cells and subsequently released from the acidic lysosomes.

### 2.4 In Vitro Anti‐Tumor Studies of PTSK@CRM

Inspired by the excellent cellular uptake of PTSK@CRM, it's in vitro anti‐tumor ability was investigated. First, the cytotoxicity of PTSK@CRM and other formulations were measured on CT26 cells by MTT assay. As shown in Figure S15 (Supporting Information), free Sim inhibited the cell viability in a dose‐dependent manner, and the half maximal inhibitory concentration (IC_50_) was 3.2 ± 0.2 µg mL^−1^. However, the cytotoxicity of PTS was lower than that of free Sim, due to the ROS‐responsive release profile (Table S1, Supporting Information). Moreover, the cytotoxicity of PTSK was slightly lower than PTS. The possible reason was that the negative charge of KYNase neutralized a portion of the positive charge of PTS. The IC_50_ of PTSK@CRM was lower than that of PTSK (8 ± 0.6 µg mL^−1^ vs 10.2 ± 0.5 µg mL^−1^), illustrating the cytotoxicity of PTSK@CRM was higher than PTSK due to the self‐recognition affinity of the biomimetic membrane.

To confirm whether PTSK@CRM could execute the killing effect through the Sim‐mediated apoptotic mechanism, the Annexin V‐FITC/PI apoptosis detection kit was employed to evaluate the apoptotic ratio after different treatments. As illustrated in Figure 3d and Figure S16 (Supporting Information), the apoptosis ratio induced by Sim was 61.8 ± 2.4%, while those of the PTS and PTSK groups decreased to 21 ± 3.2% and 18.2 ± 1.5%, respectively. The apoptosis ratio of the PTSK@CRM group was slightly higher than that of PTSK (23.1 ± 3.5% vs 18.2 ± 1.5%), which was attributed to the membrane‐mediated targeting specificity. Additionally, to gain further insights into the crucial apoptosis‐related protein, the activity and expression of cysteinyl aspartate‐specific proteinase 3 (caspase 3) were examined. The relative activity of caspase 3 in the PTSK@CRM group was significantly elevated compared with the control group (Figure 3e). Similarly, western blotting results indicated the cleaved‐caspase 3/pro‐caspase 3 ratio of the PTSK@CRM group was 9.4‐times higher than the control group (Figure 3f,g; Figure S17, Supporting Information). Based on the above results, the treatment group containing KYNase showed a slightly higher proportion of apoptotic cells at the early stage, indicating that KYNase may have an impact on the apoptosis of tumor cells.^[^ ^14^ ^]^ These results implied that PTSK@CRM could inhibit cell proliferation through cell apoptosis.

Ni W et al. once demonstrated that Sim could reduce the expression of PD‐L1 by suppressing the lncRNA SNHG29‐mediated YAP signaling axis in tumor cells.^[^ ^28^ ^]^ As shown in Figure 3f,h, the expression of PD‐L1 was all significantly decreased after different treatments. Notably, the expression level of PD‐L1 in the PTSK@CRM group was 49.8% less than that in the control group. Thus, PTSK@CRM showed the potential to reverse the immunotherapy tolerance microenvironment, especially in combination with αPD‐1.

### 2.5 In Vitro Metabolic Reprogramming Activity of PTSK@CRM

**Figure 4:**
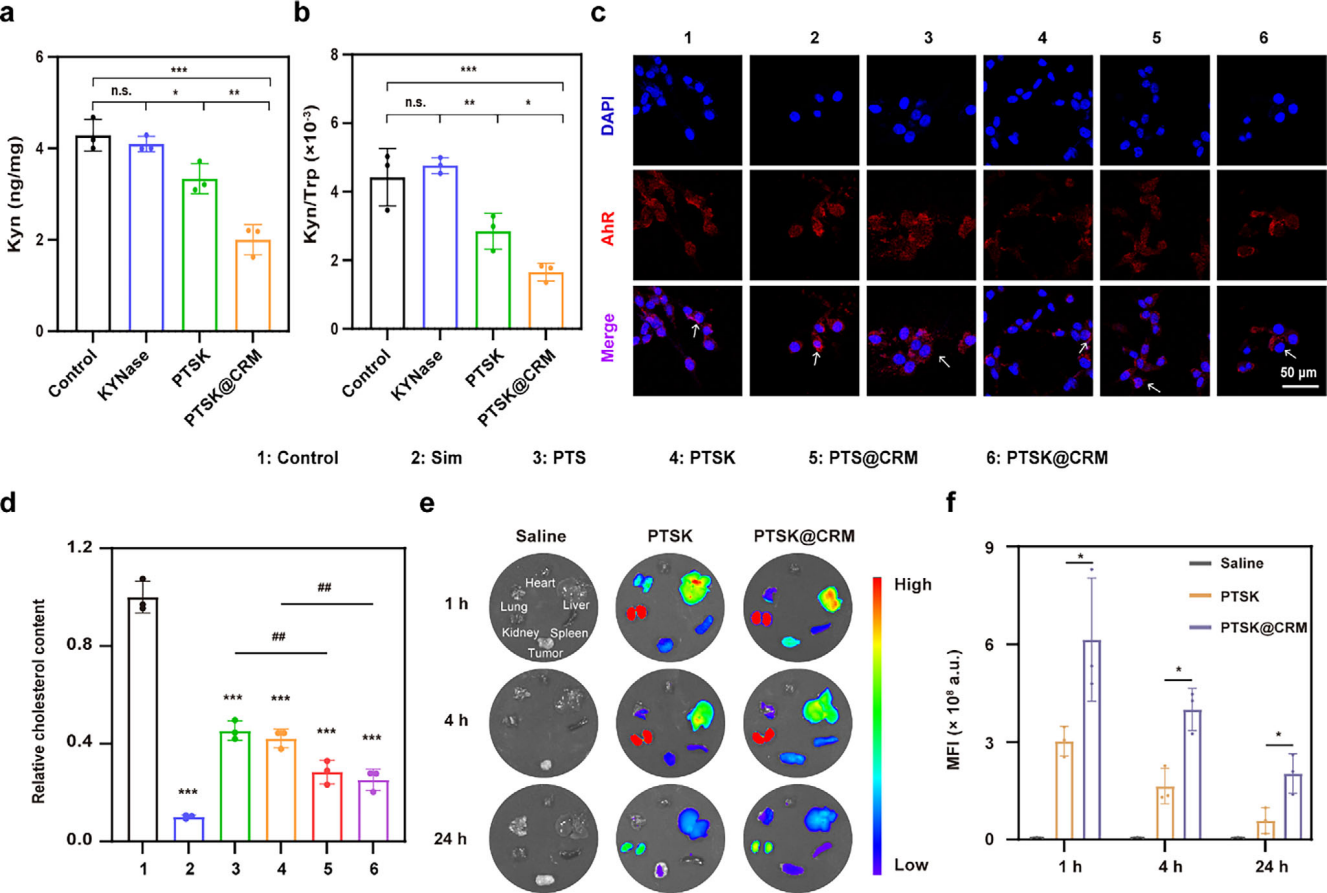
Metabolic reprogramming activity and biodistribution of PTSK@CRM. Quantitative analysis of a) Kyn content and b) Kyn/Trp ratio of CT26 cells after the incubation with KYNase, PTSK, and PTSK@CRM for 24 h (*n *= 3, one‐way ANOVA, *P < 0.05, **P < 0.01, ***P < 0.001, n.s., no significance). c) CLSM images of AhR in CT26 cells after different treatments (red: AhR, blue: DAPI‐nucleus, scale bar = 50 µm). d) Quantitative analysis of cholesterol content in CT26 cells after the incubation with different formulations for 24 h (*n* = 3, one‐way ANOVA, ^##^ *P* < 0.01, ***P < 0.001 vs Control group). e) Biodistribution images and f) MFI analysis of Cy5‐PTSK or Cy5‐PTSK@CRM at tumor tissues and major organs measured by IVIS at 1, 4, or 24 h post intravenous injection (*n* = 3, unpaired and two‐tailed *t*‐test, *P < 0.05). Data were presented as mean value ± SD.

The excellent in vitro anti‐tumor efficacy of PTSK@CRM encouraged us to investigate the metabolic reprogramming activity in CT26 cells. First, the metabolic profiles of Kyn and Trp were analyzed after the treatment with PTSK@CRM using LC‐MS. As shown in **Figures** **4**a,b and S18 (Supporting Information), there were no significant differences on the Kyn content and Kyn/Trp ratio between control and free KYNase groups. The Kyn/Trp ratio of the KYNase group was significantly lower than that of the PTSK group, because PTSK facilitated the cytosolic delivery of large‐molecule KYNase. Besides, the Kyn/Trp ratio of the PTSK@CRM group was of the lowest level, indicating that the hybrid membrane could effectively increase the cellular uptake of PTSK. Compared with the control group, the Kyn content decreased by 61.4% after the treatment with PTSK@CRM, suggesting that PTSK@CRM effectively alleviated the accumulation of Kyn. In addition, Kyn was an endogenous ligand for AhR which could induce the nuclear translocation of AhR.^[^ ^38^ ^]^ The changes of transcription factor AhR were evaluated after different treatments by CLSM (Figure 4c; Figure S19, Supporting Information). The nuclear translocated AhR fluorescence of the PTSK group was weaker than that of the PTS one. Among all the treatment groups, the CT26 cells showed the weakest nuclear translocated fluorescence in the PTSK@CRM group. All these findings suggested that the encapsulated KYNase could reduce the Kyn content and inhibit the AhR activation in tumor cells.

Previous studies have shown that Sim was capable of inhibiting the HMGCR activity and reducing the cholesterol synthesis.^[^ ^27^ ^]^ Thus, the regulation of cholesterol metabolism by PTSK@CRM was assessed by the Amplex Red Cholesterol Assay kit. As shown in Figure 4d, Sim could significantly decrease the cholesterol level in CT26 cells, and similar results were observed in other groups due to the controlled release of Sim. Remarkably, the cholesterol content of the PTS@CRM group was 17% lower than that of the PTS group, which was caused by the high endocytic efficacy of the PTS@CRM. The PTSK@CRM group exhibited the most excellent performance with a 74.7% decrease in relative cholesterol content. The inhibition of cholesterol synthesis was reported to suppress tumor growth and reinvigorate anti‐tumor immunity. Taken together, these results indicate that the PTSK@CRM group could reprogram the metabolism of Kyn and cholesterol, which paved a promising way to reverse the ITME.

### 2.6 Hemolysis and Biodistribution of PTSK@CRM

Hemolysis of therapeutic nanoparticles on RBCs was widely used to evaluate the in vitro biosafety. As shown in Figure S20 (Supporting Information), both PTSK and PTSK@CRM remained non‐hemolytic (Hemolysis ratio (%) < 5%) even at high concentrations, showing favorable biocompatibility for the in vivo application. Then in vivo biodistribution of PTSK and PTSK@CRM was evaluated on CT26 tumor‐bearing BALB/c mice. PTS and Cy 5 NHS ester were reacted at a molar ratio of 1:3 to prepare the Cy5‐labeled PTS (Cy5‐PTS). Similarly, Cy5‐PTS was used to encapsulate KYNase to construct Cy5‐PTSK, which was coated with CRM to obtain Cy5‐labeled PTSK@CRM (Cy5‐PTSK@CRM). Next, Cy5‐PTSK and Cy5‐PTSK@CRM were intravenously (*i.v*.) administered into the mice, and tumors and major organs (heart, liver, spleen, lungs, kidneys) were harvested for IVIS imaging at 1, 4, or 24 h post‐injection (Figure 4e,f). The MFI of tumors in the PTSK@CRM group at 1, 4, or 24 h post‐injection displayed 2‐, 2.4‐, and 3.5‐fold stronger than the PTSK group, respectively. The results revealed that PTSK@CRM was significantly accumulated in CT26 tumors due to the homologous targeting and prolonged circulation after the CRM coating. Besides, significant fluorescence signals were observed in the liver and kidneys following the intravenous administration of PTSK@CRM, indicating these organs were involved in the metabolic clearance and urinary excretion.^[^ ^39^ ^]^ Despite there was obvious accumulation in the liver and kidneys, no notable toxicity was detected in these organs. This favorable safety profile was attributed to the protective hybrid membrane coating, which mitigated the intrinsic toxicity of PAMAM and promoted the biocompatible clearance. In summary, the fluorescence imaging quantitatively revealed that PTSK@CRM exhibited tumor targeting, progressive accumulation, sustained retention, and drug elimination.

### 2.7 In Vivo Pharmacokinetic Analysis of PTSK@CRM

The in vivo pharmacokinetic analysis of PTSK@CRM was performed to investigate the drug metabolism profile. First, Cy5‐KYNase, Cy5‐PTSK, and Cy5‐PTSK@CRM were intravenously administered into healthy Sprague–Dawley (SD) rats to assess the pharmacokinetics of KYNase (Figure S21, Table S2, Supporting Information). The results indicated that the PTSK@CRM group exhibited a significantly prolonged elimination half‐life (t_1/2_) of KYNase compared to the free KYNase and PTSK groups. Additionally, the increased area under the curve (AUC) and mean residence time were observed in the PTSK@CRM group, suggesting that the hybrid membrane could effectively protect enzyme molecules from rapid clearance. Thus, the camouflaged hybrid membrane improved the pharmacokinetic performance and highlighted its potential to be used in enzyme‐based therapies.

Further, PTSK and PTSK@CRM were intravenously injected to SD rats to investigate the in vivo pharmacokinetics of simvastatin. As shown in Figure S21 and Table S3 (Supporting Information), the t_1/2_ values of simvastatin were 2.29 ± 0.64 and 6.77 ± 0.81 h for the PTSK and PTSK@CRM groups. Meanwhile, the AUC of the PTSK@CRM group was twofold higher than that of the PTSK group. Overall, the RBC hitchhiking strategy significantly extended the circulation time and prolonged the duration of drug action, thereby enhancing the in vivo therapeutic efficacy.

### 2.8 In Vivo Anti‐Tumor Efficacy of PTSK@CRM Combined with αPD‐1

**Figure 5:**
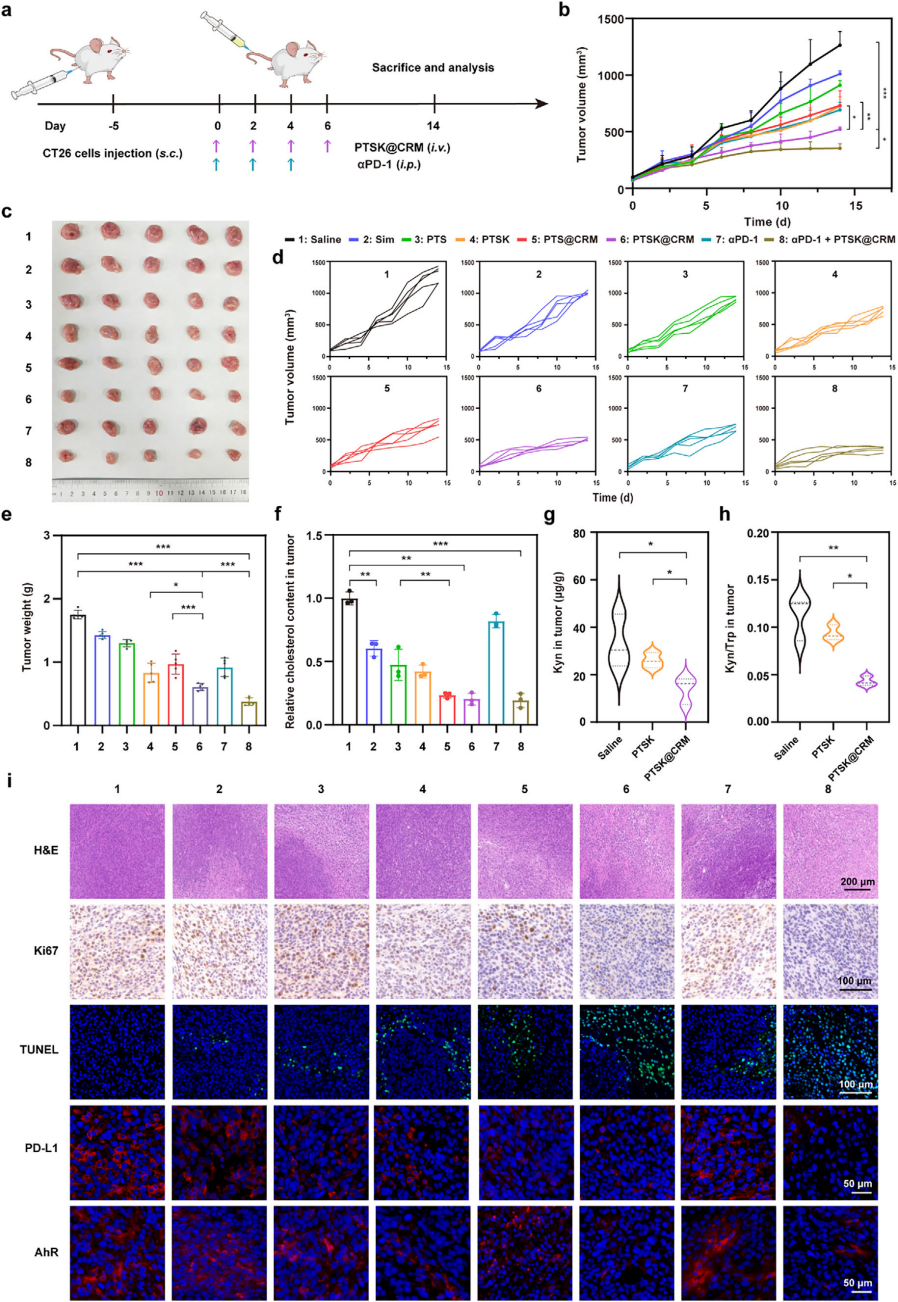
In vivo anti‐tumor efficacy of PTSK@CRM + αPD‐1 on CT26 tumor‐bearing BALB/c mice. a) Schematic depiction of experimental procedures. b) Tumor volume curves of CT26 subcutaneous tumors after different treatments (*n* = 5). c) Photographs of tumors excised on Day 14 after different treatments (*n* = 5). d) Individual tumor volume growth curves of CT26 subcutaneous tumors‐bearing mice subjected to different treatments (*n *= 5). e) The mass of excised tumors on Day 14 after different treatments (*n *= 5). f) Relative cholesterol content in CT26 tumors with different treatments on Day 14 (*n* = 3). g) The content of Kyn and h) Kyn/Trp ratio in CT26 tumor tissues of Saline, PTSK, PTSK@CRM groups on Day 14 (*n* = 3). i) Representative pathological staining (H&E staining, Ki67 IHC, TUNEL, PD‐L1 IF, and AhR IF) of tumors post‐treatment. Data were presented as mean value ± SD. Statistical significance was calculated via one‐way ANOVA analysis (*P < 0.05, **P < 0.01, ***P < 0.001).

Having demonstrated that PTSK@CRM could be effectively accumulated in the tumor after the intraveneous administration, the ability of PTSK@CRM to inhibit the tumor growth was evaluated in subcutaneous CT26 tumor‐bearing BALB/c mice. In addition, the combined treatment of αPD‐1 and PTSK@CRM was studied, as PTSK@CRM significantly reduced the PD‐L1 expression of CT26 cells (Figure 3f). When the tumor volume reached ≈100 mm^3^, the mice were randomly divided into 8 groups (*n* = 5): 1) Saline, 2) Sim, 3) PTS, 4) PTSK, 5) PTS@CRM, 6) PTSK@CRM, 7) αPD‐1, 8) PTSK@CRM + αPD‐1 (10 mg kg^−1^ on Sim basis; 20 mg kg^−1^ on KYNase basis; 10 mg kg^−1^ on αPD‐1 basis). The PTSK@CRM was intraveneously injected on Day 0, 2, 4, and 6, while αPD‐1 was intraperitoneally (*i.p*.) administrated on Day 0, 2, and 4 (**Figure** **5**a). Afterward, the tumor volume was monitored every other day, and the harvested tumors were photographed and analyzed on Day 14. Compared to the Saline group, PTSK significantly inhibited the tumor growth by 52.9% (Figure 5b–d). In comparison to PTSK, PTSK@CRM could suppress the tumor growth more significantly. The tumor weight of PTSK@CRM and PTSK was measured to be 0.61 ± 0.05 g and 0.83 ± 0.14 g on Day 14, respectively, verifying the camouflaged hybrid membrane facilitated the drug accumulation within the tumor to enhance the anti‐tumor efficacy (Figure 5e). Further, the tumor volume of the PTSK@CRM group was 28.7% lower than that of the PTS@CRM group. Although KYNase had negligible inhibition ability on the proliferation of CT26 cells, it could enhance the anti‐tumor ability of PTS@CRM. These results indicated that KYNase could specifically catalyze the conversion of Kyn to achieve anti‐tumor efficacy. Additionally, PTSK@CRM + αPD‐1 displayed the most dramatic tumor‐suppression effect, and the tumor weight was 0.38 ± 0.06 g. The underlying mechanism might be that PTSK@CRM could reduce the PD‐L1 expression of tumor cells and increase the infiltration of immune cells. Meanwhile, αPD‐1 blocked the “Don't eat me” signal between T cells and tumor cells, which further activated the T cells to improve immune tolerance and enhance therapeutic efficacy.

Next, metabolic reprogramming in tumors was evaluated after different treatments on Day 14. First, the cholesterol levels in the tumor were examined to assess the regulation of lipid metabolism. As shown in Figure 5f, free Sim could significantly reduce the relative cholesterol content in tumors, which was consistent with the previous report.^[^ ^27^ ^]^ Moreover, the PTS@CRM group exhibited a 23.9% stronger inhibitory effect on cholesterol synthesis than the PTS group. The homologous targeting of biomimetic membrane CRM contributed to the accumulation of nanoparticles in tumors to enhance the inhibitory of cholesterol synthesis. Second, Kyn metabolism, including Trp content, Kyn content, and Kyn/Trp ratio within tumors, was evaluated after the treatment with saline, PTSK, and PTSK@CRM. Both PTSK and PTSK@CRM were unable to reduce the Trp content in tumors (Figure S22, Supporting Information), while Kyn content was significantly lower than the Saline group (Figure 5g). The Kyn/Trp ratio of the PTSK@CRM group was also significantly lower than that of the PTSK group (0.041 ± 0.004 vs 0.093 ± 0.006), as CRM remarkably enhanced the bioavailability of KYNase (Figure 5h). In conclusion, PTSK@CRM effectively reprogrammed the cholesterol and Kyn metabolism in tumors, which provided a basis for reversing the ITME and enhancing the anti‐tumor efficacy.

To explore the mechanism underlying the superior anti‐tumor efficacy mediated by PTSK@CRM, hematoxylin and eosin (H&E) staining, immunohistochemistry (IHC), and immunofluorescence (IF) of tumor tissues were conducted (Figure 5i; Figure S23, Supporting Information). H&E staining results of the PTSK@CRM + αPD‐1 group displayed the most extensive necrosis area, and the tumor growth was also markedly inhibited based on the Ki67 staining. Similarly, the TUNEL assay confirmed the substantial presence of apoptotic cells in the PTSK@CRM and PTSK@CRM + αPD‐1 groups, validating their excellent therapeutic efficacy. Subsequently, the expression of PD‐L1 and AhR in tumors was investigated by IF. The red fluorescence of PD‐L1 was obviously suppressed in the PTS@CRM, PTSK@CRM, and PTSK@CRM + αPD‐1 groups. The reduction of PD‐L1 expression on cell surfaces disrupted the interaction between tumor cells and cytotoxic T lymphocytes (CTLs), thereby further activating the adoptive immune responses. Moreover, consistent with the in vitro analysis of AhR expression, there was strong red fluorescence in the saline‐treated tumor, indicating that high Kyn content activated both the AhR expression and the nuclear translocation. Notably, the AhR fluorescence signals were diminished in both cellular compartments and the nucleus after the PTSK@CRM treatment. The possible reason was that Kyn was hydrolyzed by KYNase to reduce the activation of the downstream Kyn‐AhR pathway. Collectively, PTSK@CRM successfully integrated the Kyn pathway with cholesterol metabolism, which is beneficial to enhance the anti‐tumor immunity.

Meanwhile, the in vivo biosafety of PTSK@CRM was evaluated for the clinical potential as anti‐tumor agents. No significant changes of body weight were observed for all the treatments (Figure S24, Supporting Information). H&E staining of major organs (heart, liver, spleen, lungs, and kidneys) revealed no notable signs of inflammatory infiltration at 0, 1, 4, and 24 h after the administration of PTSK@CRM (Figure S25, Supporting Information). Similarly, there were no obvious changes for all these groups on Day 14 (Figure S26, Supporting Information). Besides, there were no significant abnormalities detected in biochemical indicators for renal and liver function, including alanine transaminase (ALT), aspartate aminotransferase (AST), creatinine (CRE), blood urea nitrogen (BUN), and alkaline phosphatase (ALP), at 1, 4, and 24 h after the PTSK@CRM administration (Figure S27, Supporting Information). Consistent with H&E images, serum biochemical findings showed no significant variation on Day 14 after different treatments (Figure S28, Supporting Information). Immunogenicity is one of the key factors influencing the in vivo function of protein‐based therapeutics. To evaluate the immunogenicity of PTSK@CRM, the serum levels of IgM and IgG antibodies in healthy BALB/c mice were measured after the intravenous administration PTSK@CRM every other day for 4 times. Clearly, there were almost no changes in the levels of antibodies after multiple injections of PTSK@CRM in comparison to the control group (Figure S29, Supporting Information). All these results demonstrated that PTSK@CRM were well‐tolerated and exhibited high biosafety.

### 2.9 In Vivo Anti‐Tumor Immunity of PTSK@CRM Combined with αPD‐1

**Figure 6:**
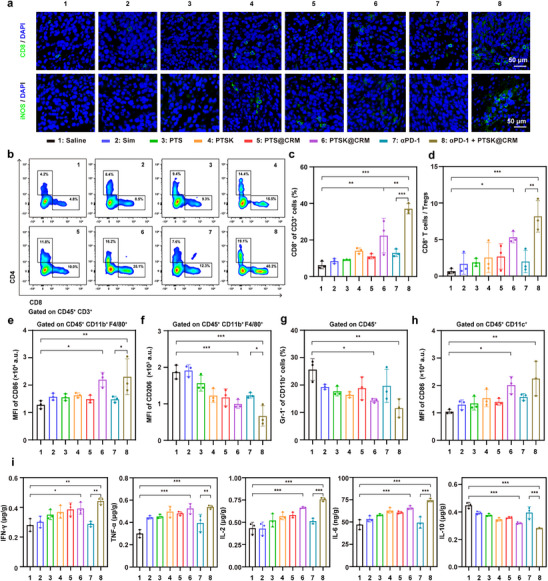
In vivo activation of immune responses on the CT26‐tumor‐bearing BALB/c mice model. a) IF staining images for CD8 and iNOS in CT26 tumors after different treatments. Blue represented the nucleus, and green color represented CD8 or iNOS. scale bar = 50 µm. b) Representative flow cytometric plots of CD4^+^ and CD8^+^ T cells and c) the quantitative analysis of the percentage of CD8^+^ T cells in CT26 tumors after different treatments. d) Quantitative analysis of the percentage ratio of CD8^+^ T cells/Tregs in CT26 tumors after different treatments. Quantitative analysis of e) M1‐like and f) M2‐like macrophages in CT26 tumors after different treatments through median fluorescence indensity (MFI) of CD86 and CD206, respectively. Quantitative analysis of g) MDSCs and h) mDCs in CT26 tumors after different treatments. i) ELISA analysis for the levels of IFN‐γ, TNF‐α, IL‐2, IL‐6, and IL‐10 in CT26 tumor tissues after different treatments. Data were presented as mean ± SD (*n* = 3). Statistical significance was calculated via one‐way ANOVA analysis (*P < 0.05, ** *P* < 0.01, *** *P* < 0.001).

Metabolic reprogramming within the TME has been demonstrated to remodel the landscape of immune cells and reverse the immune microenvironment from “cold” to “hot”.^[^ ^13^ ^]^ Therefore, immune cells and cytokines in tumors were analyzed on Day 14 to elucidate the implications of PTSK@CRM‐mediated metabolism rebuilding on immune responses. The infiltration of CTLs (CD8^+^ cytotoxic T cells), Th cells (CD4^+^ helper T cells), and TAMs (iNOS^+^ M1 and CD206^+^ M2 phenotype) was evaluated by IF (**Figure** **6**a; Figure S30, Supporting Information). Compared with the Saline group, more presence of CTLs, Th cells, and M1‐like macrophages could be clearly observed in the PTSK@CRM‐treated tumors, with the most remarkable number of these cells in the PTSK@CRM + αPD‐1 group. Meanwhile, compared to either the PTSK@CRM or αPD‐1 group, the PTSK@CRM + αPD‐1 group led to a decreased ratio of M2‐like macrophages (Figure S30, Supporting Information). Subsequently, the proportions of immune cells (CD3^+^CD4^+^ T cells, CD3^+^CD8^+^ T cells, CD3^+^CD4^+^CD25^+^Foxp3^+^ Tregs, CD11b^+^F4/80^+^CD86^+^ M1‐like macrophages, CD11b^+^F4/80^+^CD206^+^ M2‐like macrophages, CD11b^+^Gr‐1^+^ MDSCs, and CD11c^+^CD80^+^CD86^+^ mature dendritic cells, mDCs) in tumors were analyzed using flow cytometry (Figure S31, Supporting Information). Remarkably, CD8^+^ T cells of the PTSK@CRM group were significantly higher than the Saline group, and the PTSK@CRM + αPD‐1 group further enhanced the number of CD8^+^ T cells (Figure 6b,c). Additionally, compared to the αPD‐1 group, PTSK@CRM + αPD‐1 led to an increase in CD8^+^ and CD4^+^ T cells, with frequencies reaching 37.1 ± 2.3% and 18 ± 3%, respectively (Figure 6c; Figure S32, Supporting Information). As shown in Figure S33 (Supporting Information), the percentage of Foxp3^+^CD25^+^ cells in the PTSK@CRM + αPD‐1 group was significantly lower than the Saline group, indicating the reduction of Treg cells by the combined administration. Besides, the ratio of CD8^+^ T cells/Tregs was analyzed to evaluate the activity of T cells, which exerted cytotoxic effects in TME. As shown in Figure 6d, the CD8^+^ T cells/Tregs ratio of the PTSK@CRM + αPD‐1 group was 12.2‐fold and 4.1‐fold higher than that of the Saline and αPD‐1 groups, respectively. Previous studies have demonstrated that reprogramming both cholesterol and Kyn metabolism greatly promoted the repolarization of macrophages from M2 to M1 phenotype.^[^ ^15^, ^31^ ^]^ As shown in Figure 6e and Figure S34a (Supporting Information), PTSK@CRM and PTSK@CRM + αPD‐1 groups significantly increased the population of M1 macrophages compared with Saline group. Meanwhile, the proportion of M2 macrophages in the PTSK@CRM + αPD‐1 group was 0.4 and 0.5 times than that of the Saline and αPD‐1 groups, respectively (Figure 6f; Figure S34b, Supporting Information). As a cluster of immunosuppressive cells, MDSCs induced an increase in Treg cells and inhibited the function and proliferation of CTLs, thereby enhancing the negative regulatory effects on immune response.^[^ ^40^ ^]^ The percentage of MDSCs in the PTSK@CRM and the PTSK@CRM + αPD‐1 groups decreased by 44% and 54.8% in comparison to the Saline group, respectively (Figure 6g; Figure S35, Supporting Information). Additionally, the necrosis of tumor cells would release tumor‐associated antigens to stimulate DC maturation, increase antigen presentation, and promote T lymphocyte differentiation. As illustrated in Figure 6h and Figure S36 (Supporting Information), the proportion of mDCs in the PTSK@CRM + αPD‐1 group was significantly higher than that of the αPD‐1 group. In summary, PTSK@CRM promoted the infiltration of CTLs, Th cells, and mDCs, reduced the infiltration of MDSCs, enhanced the polarization from M2 to M1‐like macrophages, and decreased the differentiation from T cells to Tregs. The combined administration of PTSK@CRM + αPD‐1 markedly amplified the therapeutic efficacy and inherent immunoadjuvant property of PTSK@CRM.

For the cellular responses in spleens (Figures S37–S40, Supporting Information) and tumor‐draining lymph nodes (TDLNs) (Figures S41–S43, Supporting Information), the proportions of CD8^+^ T cells, CD4^+^ T cells, and mDCs were analyzed by flow cytometry. CD8^+^ T cells, CD4^+^ T cells, and mDCs have been significantly elevated in the PTSK@CRM group, which were consistent with the data in tumors. Moreover, the PTSK@CRM + αPD‐1 group showed a greater performance in the improvement of these immune cells than the PTSK@CRM and αPD‐1 groups. In addition, the infiltration level of immune cells within TME mirrored similar trends with bioinformatic analysis of clinical patient data presented in Figure S2c,d (Supporting Information), in which the levels of Kyn and cholesterol were closely correlated to the immune cell responses.

Finally, pro‐inflammatory cytokines (TNF‐α, IFN‐γ, IL‐2, IL‐6) and anti‐inflammatory cytokines (IL‐10) in tumor and serum were quantified on Day 14 by enzyme‐linked immunosorbent assays (ELISA) (Figure 6i; Figure S44, Supporting Information). Notably, IFN‐γ and IL‐2 were markedly upregulated in the PTSK@CRM group compared with the Saline group, indicating the activation of CD8^+^ T cells. The increased DC maturation alongside the elevated level of pro‐inflammatory cytokines contributed to the sustained activation of T cells, thereby enhancing the anti‐tumor efficacy. Moreover, IL‐6 and TNF‐α, key cytokines secreted by M1‐like macrophages, were sharply improved in the PTSK@CRM group. Similarly, higher levels of secreted pro‐inflammatory cytokines have been detected in the PTSK@CRM + αPD‐1 group. Conversely, IL‐10, which inhibited the maturation and secretion of antigen‐presenting cells, was significantly inhibited in the PTSK@CRM + αPD‐1 group. Taken together, the elevated secretion of proinflammatory cytokines contributed to the killing of tumor cells, which was consistent with the changes in the tumor growth and tissue staining (H&E, Ki‐67, and TUNEL). These findings underscored effective tumor suppression and robust activation of the immune response. Meanwhile, PTSK@CRM has shown the possibility to achieve favorable long‐term therapeutic efficacy due to the elevation of CD8^+^ T cells, M1‐like macrophages, and matured DCs at the endpoint. The strategy that combines PTSK@CRM with anti‐PD‐1 has great potential to mediate the immunomodulation and improve the therapeutic outcome.

### 2.10 In Vivo Anti‐Metastatic Efficacy of PTSK@CRM in CT26 Lung Metastasis Mice Model

**Figure 7:**
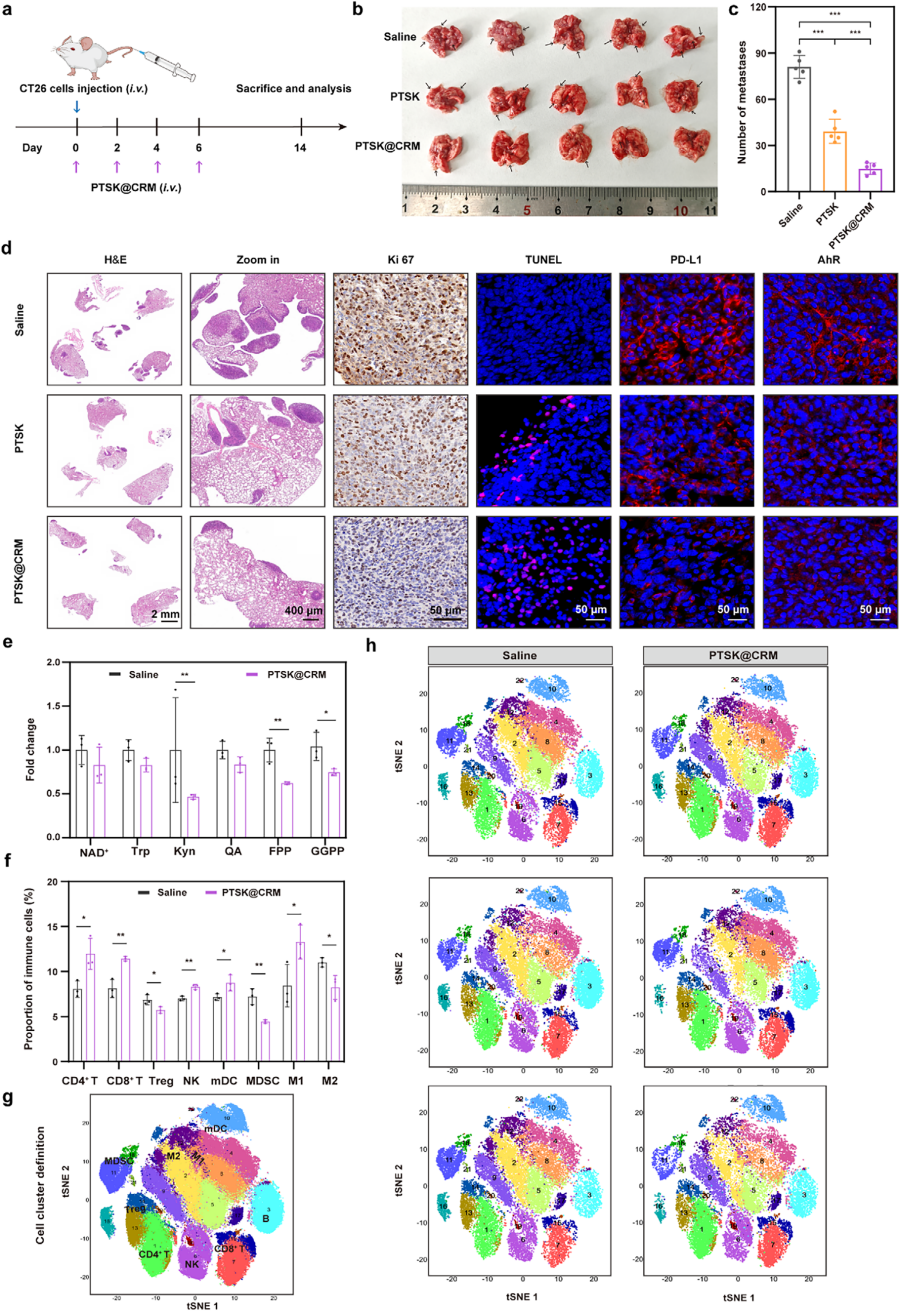
In vivo anti‐metastatic efficacy of PTSK@CRM in the CT26 lung metastasis mouse model. a) Schematic depiction of experiment procedures. b) Appearance, c) the number of metastases, and d) representative pathological staining (H&E staining, Ki67 IHC, TUNEL, PD‐L1 IF, and AhR IF) of lungs on Day 14 after the treatment with PTSK or PTSK@CRM (*n* = 5, one‐way ANOVA). The black arrow indicated the representative of lung metastases. e) The content of relevant metabolites in CT26 lung metastases on Day 14 (*n* = 3, unpaired and two‐tailed *t‐*test). f) The proportion of specific immune cells in lung metastases detected by CyTOF on Day 14 (*n* = 3, unpaired and two‐tailed *t*‐test). g) t‐SNE plots of the main immune cell subsets in lung metastases. h) t‐SNE analysis of lung metastases in Saline and PTSK@CRM groups by CyTOF (*n* = 3). Data were presented as mean value ± SD (*P < 0.05, **P < 0.01, ***P < 0.001).

Clinically, CRC demonstrates high aggressiveness with a marked propensity for distant metastasis.^[^ ^41^ ^]^ Thus, the CT26 lung metastasis mouse model was employed to check whether PTSK@CRM possessed complex metastatic diseases (**Figure** **7**a). The metastatic model was established via the intravenous inoculation of 2.5 × 10⁵ CT26 cells into female BALB/c mice. The mice were randomly divided into three treatment groups (*n* = 5): 1) Saline, 2) PTSK, and 3) PTSK@CRM. 100 µL of saline, PTSK, or PTSK@CRM were intravenously injected on Days 0, 2, 4, and 6. On Day 14, the lungs were collected for macroscopic evaluation and further analysis. Clearly, a large number of lung metastases could be observed in the Saline group during the metastasis period of 14 days, and the number of lung metastasis foci was reduced in the PTSK and PTSK@CRM treatment groups (Figure 7b). Quantitative assessment of pulmonary metastasis showed that the number of metastatic nodules was 39.2 ± 7 and 14.8 ± 3.3 in the PTSK and PTSK@CRM groups, respectively, much lower than the Saline group (81 ± 6.7), as shown in Figure 7c. These results indicated that the anti‐metastatic efficacy of PTSK@CRM was better than PTSK, which was probably attributed to the coating of a biomimetic hybrid membrane to enhance the tumor‐specific targeting ability and extend the systemic circulation time. All the treatments were well‐tolerated, as evidenced by the stable body weight in these groups (Figure S45, Supporting Information).

Histopathological evaluation confirmed a substantial reduction in the metastatic foci of lungs in the PTSK@CRM group in comparison to the Saline and PTSK groups (Figure 7d). Consistent with this observation, Ki67 and TUNEL analysis demonstrated that PTSK@CRM significantly suppressed the proliferation of tumor cells and enhanced the apoptosis within metastatic nodules (Figure 7d; Figure S46, Supporting Information). Quantitative analysis revealed a ≈60% downregulation of PD‐L1 expression, indicating the effective attenuation of this critical immune checkpoint. Further, remarkable suppression of AhR activation could be observed, accompanied by an over 50% reduction in Kyn level (Figure 7e). This metabolic reprogramming could extend to the mevalonate pathway, in which the levels of key intermediates farnesyl pyrophosphate (FPP) and geranylgeranyl pyrophosphate (GGPP) decreased in the PTSK@CRM group (Figure 7e). The Rab5 geranylgeranylation, a post‐translational modification dependent on GGPP from the mevalonate pathway, facilitated the translocation of antigenic cargo to lysosomes. Sim and PTSK@CRM significantly reduced Rab5‐lysosome colocalization, indicating suppression of Rab5 geranylgeranylation and antigen degradation (S47, Supporting Information). The coordinated regulation of the Kyn‐AhR pathway and mevalonate metabolism demonstrated that PTSK@CRM reshaped ITME to overcome the immunosuppressive barriers.

To comprehensively characterize the immune landscape of lung metastases, high‐dimensional immune profiling was performed by mass cytometry (cytometry by time of flight, CyTOF). t‐Distributed stochastic neighbor embedding (t‐SNE) plots revealed that there were significant phenotypic alterations of tumor‐infiltrating immune cells following the PTSK@CRM treatment (Figure 7f–h; Figures S48,S49, Supporting Information). Compared to the Saline group, PTSK@CRM exhibited a remarkable improvement of immunostimulatory populations, including CD4⁺ T cells, CD8⁺ T cells, NK cells, mDCs, and M1 macrophages. Besides, marked reductions could be observed in immunosuppressive cell subsets, particularly for Tregs, MDSCs, and M2 macrophages. This immunophenotypic shift was aligned with the findings in the CT26 subcutaneous tumors (Figure 6), demonstrating the microenvironmental reprogramming across distinct tumor models.

Metabolic profiling was conducted to evaluate the potential off‐target effects of PTSK@CRM through the quantification of NAD⁺, oxysterols, and key intermediates of the mevalonate and Kyn pathways in serum and liver tissues from the CT26 lung metastasis mice model. No statistically significant alterations were observed in the systemic levels of NAD⁺, Trp, Kyn, quinolinic acid (QA), FPP, and GGPP between control and PTSK@CRM groups (Figure S50, Supporting Information), demonstrating the preserved systemic metabolic homeostasis. The minimal off‐target effects confirmed the excellent biocompatibility of PTSK@CRM. Thus, PTSK@CRM could achieve precise metabolic intervention at tumor sites while maintaining the global metabolic balance, highlighting its exceptional targeting specificity and favorable safety profile.

In summary, PTSK@CRM demonstrated potent anti‐metastatic activity through immune‐metabolic reprogramming of TME. It could effectively suppress the immunosuppressive pathways, enhance the antitumor immunity, and achieve targeted therapeutic efficacy without compromising systemic metabolic balance.

### 2.11 In Vivo Anti‐Tumor Efficacy of PTSK@CRM in Subcutaneous MC38 Tumor Model

Further, the MC38 murine colon adenocarcinoma model was used to investigate the broader applicability of PTSK@CRM. The MC38 cells were subcutaneously implanted into the right flank of C57BL/6 mice, and these mice were randomly divided into three groups after 7 days: Saline, PTSK, and PTSK@CRM (Figure S51a, Supporting Information). The tumor volume was monitored throughout the treatment period, and the histopathological analysis was performed on the excised tumors. In comparison to the control group, the PTSK and PTSK@CRM groups could significantly suppress the tumor growth by 60.7% and 82.7%, respectively (Figure S51b–d, Supporting Information). Meanwhile, the PTSK@CRM group showed a stronger inhibitory effect than the non‐coated PTSK. These results were consistent with the data on the CT26 tumor model, which was probably caused by the coating of the hybrid membrane to promote the blood circulation time and enhance the tumor‐targeting capacity through specific adhesion proteins and integrins on the membrane of MC38 cells.^[^ ^42^ ^]^ There were no significant differences in the body weight between these three groups (Figure S51e, Supporting Information). Histopathological evaluation revealed that the PTSK@CRM treatment significantly increased the number of TUNEL‐positive cells and markedly reduced the number of Ki67‐positive cells compared to the Saline group, indicating the enhanced apoptosis and suppressed proliferation of tumor cells. Moreover, PTSK@CRM effectively downregulated the PD‐L1 expression and inhibited the AhR activation in TME (Figures S51f,S52, Supporting Information). Collectively, these data from the MC38 murine colon adenocarcinoma model were consistent with the robust antitumor ability observed in the CT26 lung metastasis mice model, thereby strengthening the general applicability of PTSK@CRM.

Further validation was conducted by cytokine recall assay in both MC38 subcutaneous and CT26 lung metastasis mouse models. Peripheral blood mononuclear cells (PBMCs) isolated from the tumor‐bearing mice were re‐stimulated with tumor lysate, and cytokine levels in the culture supernatants were quantified. IL‐2, TNF‐α, and IFN‐γ were secreted by T cells, which could reflect the activation of T cells. Significantly elevated concentrations of IL‐2, TNF‐α, and IFN‐γ could be detected in the PTSK@CRM group compared to the Saline and PTSK groups (Figures S53,S54, Supporting Information), indicating the enhanced antigen‐specific T cell responses. These results and CyTOF data highlighted the role of the hybrid membrane coating in improving the targeting and sustaining immune activation.

The anti‐tumor efficacy across three kinds of tumor models underscored the ability of PTSK@CRM to induce robust immunity through the integrated immune‐metabolic modulation. By suppressing the Kyn and mevalonate pathways, PTSK@CRM alleviated the immunosuppression and promoted the effector T cell function. These results established a compelling basis for the translational development of PTSK@CRM in advanced colorectal cancer.

## 3 Conclusion

In summary, biomimetic and pH/ROS dual‐responsive nanoparticles loaded with Sim and KYNase were successfully constructed to regulate the metabolic reprogramming of tumors and enhance the immunotherapeutic efficacy. The IDO1 and HMGCR were identified to be overexpressed in COAD tissues at mRNA and protein levels through bioinformatic analysis, leading to the accumulation of Kyn and cholesterol in TME. The PTSK@CRM nanoparticles were stable, uniform, and monodisperse spherical structures, and could release Sim and KYNase in the buffers with low pH and high H_2_O_2_. The excellent tumor‐targeting ability of PTSK@CRM was beneficial to promote cell apoptosis and inhibit cell proliferation. Moreover, PTSK@CRM reduced the levels of cholesterol and Kyn and decreased the activation of the Kyn‐AhR pathway, thereby regulating the tumor metabolism. After the intravenous injection of PTSK@CRM on CT26 subcutaneous tumor‐bearing and lung metastasis mice, they augmented the infiltration of immune cells, facilitated the differentiation of T cells, promoted the polarization toward M1‐like macrophages, enhanced the maturation of DCs, and increased the secretion of pro‐inflammatory cytokines. Further, PTSK@CRM + αPD‐1 significantly inhibited the tumor growth, reversed the ITME, and reshaped the immune landscape. Moreover, the excellent anti‐tumor efficacy of PTSK@CRM was observed in the MC38 murine colon adenocarcinoma model. In conclusion, the immune‐metabolic therapy based on PTSK@CRM could reverse the cholesterol/Kyn‐induced ITME to enhance the anti‐tumor immunity of αPD‐1.

## 4 Experimental Section

### Materials

Polyamidoamine (PAMAM, G5.0, 5 wt.% in methanol) was purchased from Macklin Biochemical (Shanghai, China). TK‐COOH was purchased from Adamas (Shanghai, China). Sim, pyridoxal phosphate (PLP), 4‐dimethylaminopyridine (DMAP), 1,3‐dicyclohexylcarbodiimide (DCC), 1‐(3‐dimethylaminopropyl)‐3‐ethylcarbodiimide hydrochloride (EDC), imidazole, fluorescein isothiocyanate (FITC), and *N*‐hydroxy succinimide (NHS) were obtained from Aladdin (Shanghai, China). Ni^2+^‐Sepharose 6FF and Ficoll plus 1.083 were purchased from Solarbio (Beijing, China). Kyn was provided by Alab Chemical Technology (Shanghai, China). Cell membrane protein and cytoplasmic protein extraction kits, phenylmethanesulfonyl fluoride (PMSF), Lyso‐tracker red, 4′,6‐diamidino‐2‐phenylindole (DAPI), 3,3′‐dioctadecyloxacarbocyanine perchlorate (DIO), 1,1′‐dioctadecyl‐3,3,3′,3′‐tetramethylindodicarbocyanine, 4‐chlorobenzenesulfonate salt (DID), and 3‐(4,5)‐dimethylthiahiazo(‐z‐yl)‐3,5‐di‐phenytetrazoliumromide (MTT) were purchased from Beyotime (Shanghai, China). The BCA protein assay kit was acquired from SparkJade (Qingdao, China). Methanol (HPLC grade), dimethyl sulfoxide (DMSO), Amplex Red cholesterol assay kit, anti‐PD‐L1 antibody (ab213480), anti‐Caspase‐3 antibody (ab32351), anti‐Band 3 antibody (PA5‐80030), and anti‐E‐Cadherin antibody (ab231303) were provided by Thermo Fisher Scientific (Waltham, MA, USA). Anti‐Na, K‐ATPase antibody (#3010) and anti‐Rab5 antibody (#3547) were obtained from Cell Signaling Technology (Danvers, MA, USA). The Annexin V‐FITC/PI apoptosis detection kit was purchased from Bestbio (Nanjing, China). Caspase‐Glo 3/7 activity assay kit was provided by Promega (Madison, WI, USA). IFN‐γ was purchased from Peprotech (Cranbury, NJ, USA). Cy5 NHS ester (Cy5‐SE) was purchased from Med Chem Express (Shanghai, China). Anti‐PD‐1 antibody (αPD‐1, RMP1‐14) was provided by Bio X Cell (NH, USA). Anti‐AhR antibody (67785‐1) and anti‐Ki‐67 antibody (27309‐1) were purchased from Proteintech (Wuhan, China). Antibodies against FITC‐CD45, BV421‐CD25, BV421‐CD80, BV605‐CD8, BV605‐F4/80, BV650‐CD3, BV650‐Gr‐1, PE‐CD49b, PE‐CD11c, PE‐Cy7‐CD86, APC‐CD4, APC‐CD206, APC‐Cy7‐CD11b, and CD16/32 were provided by Biolegend (San Diego, CA, USA). Anti‐Percp‐Cy5.5‐Foxp3, Live/Dead AQUA, and Foxp3/transcription factor staining buffer set were purchased from eBioscience (MA, USA). ELISA kits for detecting IFN‐γ, TNF‐α, IL‐2, IL‐6, and IL‐10 were purchased from Anoric Biotechnology (Tianjin, China). ELISA kits for IgM and IgG were purchased from MultiSciences Biotechnology (Zhejiang, China). Human serum (HS, JS16089) was provided by Jushi Biotechnology (Henan, China). Cell‐ID Cisplatin, Cell‐ID 20‐Plex Pd Barcoding Kit, and MaxPar X8 Polymer Kit were obtained from Standard BioTools (Shanghai, China). LunaStain Cell Staining Buffer, Luna Fix Cell Fix Buffer, and Ir‐DNA Intercalator Reagent were purchased from Polaris (Shanghai, China).

### Expression and Pathway Analysis of *KYNU* and *HMGCR* in COAD Tissues

RNA‐sequencing profiles of *IDO1*, *KYNU*, and *HMGCR* in COAD tissue and normal colon tissue were acquired from the TCGA database and analyzed using the GEPIA platform.^[^ ^43^ ^]^ The IHC staining images of *KYNU* (or *HMGCR*) expression in COAD tissue were obtained from the HPA database. To assess the correlation of *KYNU* (or *HMGCR*) expression level and pathway enrichment, KEGG analysis was performed through the Linkedomics database.^[^ ^44^ ^]^ Spearman correlation analysis was performed by the GEPIA database between the *KYNU* (or *HMGCR*) mRNA level and the level of 10 gene signatures of effector T cells and helper T cells. The correlation between the *KYNU* (or *HMGCR)* expression level and the infiltration of immune cells was analyzed through the TIMER 2.0 database.^[^ ^45^ ^]^

### Synthesis of PAMAM‐TK‐Sim

TK‐COOH (504.8 mg), DCC (618.9 mg), DMAP (24.4 mg), and Sim (837.5 mg) were dissolved in anhydrous CH_2_Cl_2_ (30 mL) and reacted at 0 °C for 1.5 h under continuous stirring. Subsequently, an excess of deionized water was added to terminate the reaction. Ethyl acetate was added to extract the crude products, following the rinsing with deionized water three times. After drying with anhydrous Na_2_SO_4_, TK‐Sim was isolated via column chromatography (ethyl acetate/methanol, v/v, 3/1). The structure of TK‐Sim was characterized by ^1^H NMR spectroscopy (Bruker AV 300 MHz system, Bruker Physik AG, Germany) in CDCl_3_. In addition, TK‐Sim was analyzed by electrospray ionization‐quadrupole‐time of fly mass spectrometry (ESI‐Q‐TOF MS, Agilent, China).

TK‐Sim (272 mg), EDC (798 mg), and NHS (240 mg) were dissolved in DMSO (5 mL) and reacted at room temperature for 4 h. Then, PAMAM (G5.0, 1 g) dissolved in methanol was added to the above solution, and the mixture was stirred at room temperature overnight. The solution was dialyzed (MWCO: 3500 Da) for 72 h against deionized water and lyophilized to obtain PAMAM‐TK‐Sim (PTS). The structure of PTS was characterized by ^1^H NMR spectroscopy in CD_3_OD.

### Expression and Purification of KYNase

The expression and purification of KYNase were conducted as previously reported.^[^ ^19^ ^]^ Briefly, the KYNase variant amplicon with N‐terminal 6 × histidine was inserted into the pMAL‐c2x plasmid, and the recombinant plasmid was transformed into *E. coli*. BL21(DE3). The transformants were inoculated into 5 mL of LB medium supplemented with ampicillin (50 µg mL^−1^) and cultured at 37 °C overnight. Then the culture was transferred to TB medium containing 50 µg mL^−1^ ampicillin at a 1:100 ratio. After the incubation at 37 °C until the OD_600_ value reached 0.8, IPTG (final concentration: 0.5 mm) was added to induce the expression of KYNase at 18 °C overnight. Through the centrifugation (4 °C, 4000 rpm, 20 min), the collected bacteria sediment (1 g) was resuspended in 15 mL of lysis buffer (100 mm sodium phosphate, pH 8, 1 mm PLP, 300 mm NaCl, 25 mm imidazole, 0.1% Tween‐20) and lysed by sonication (Scientz, China). The lysate was then centrifugated at 8000 rpm for 30 min, and the supernatant was applied to a pre‐equilibrated Ni‐NTA column (5 mL). KYNase was eluted with elution buffer (100 mm sodium phosphate, pH 8, 1 mm PLP, 300 mm NaCl, 250 mm imidazole) and subsequently dialyzed against the buffer solution (100 mm sodium phosphate, pH 8, 1 mm PLP, 300 mm NaCl) at 4 °C for 48 h. Finally, the protein solution was concentrated with Amicon Ultra centrifugal filters (MWCO: 30 kDa) and stored at −80 °C for further use.

The purity of KYNase was determined by SDS‐PAGE analysis. Michaelis–Menten kinetic parameters of KYNase against Kyn were calculated at 37 °C with Kyn concentration ranging from 0 to 0.8 mm and KYNase of 1 µm. The absorbance at 365 nm was measured to quantify the Kyn degradation using a UV–vis 2700i spectrophotometer (Shimadzu, Japan).

### Cell Lines and Animals

NIH/3T3 cells (murine embryo fibroblasts), CT26 cells (murine colorectal cells), 4T1 cells (murine breast cancer cells), and MC38 cells (murine colorectal cells) were purchased from the American Type Culture Collection (ATCC). CT26 and 4T1 cells were cultured in RPMI 1640 medium supplemented with 10% (v/v) FBS and 1% penicillin and streptomycin with 5% CO_2_ at 37 °C. NIH/3T3 and MC38 cells were cultured in DMEM harboring 10% (v/v) FBS under the same conditions. Female BALB/c mice (6–8 weeks old) and C57BL/6 mice (6–8 weeks old) were purchased from Beijing Vital River Laboratory Animal Technology Co., Ltd. (Beijing, China). Female SD rats (6–8 weeks old) were purchased from Liaoning Changsheng Biotechnology Co., Ltd. (Liaoning, China). All animal experiments were complied with the Laboratory Animal Management Regulations of Jilin University and approved by the Institution Animal Ethics Committee of Jilin University (approval NO.: 2023YNPZSY1101, 2024YNPZSY0303, 2025YNPZSY0809).

### Preparation and Characterization of CT26‐RBC (CRM) Hybrid Membrane

Fresh whole blood was collected from BALB/c mice, centrifuged (4 °C, 3000 rpm, 10 min) in heparin‐pretreated tubes, and washed twice with precooling PBS to obtain RBCs. Then, RBCs and 0.25 × PBS were mixed at a volume ratio of 1:30, and the sample was incubated at 4 °C for hemolysis (4 h). The hemoglobin was removed by centrifugation (4 °C, 14 000 rpm, 20 min) to obtain the precipitates of RM. The collected RM was repeatedly washed with 0.25 × PBS until it became achromatous and then preserved at −80 °C for further use.

The CT26 cell membrane was extracted in accordance with the instructions of the cell membrane protein and cytoplasmic protein extraction kit (Beyotime, China). Briefly, 20–50 million cells were scraped off and collected after centrifugation at 1000 rpm for 5 min. The collected cells were washed with PBS and resuspended in membrane protein extraction reagent A (1 mL) containing PMSF (1 mm). The mixture was incubated in an ice bath (15 min), ground, and centrifuged for 10 min (4 °C, 3500 rpm) to collect the supernatant. Finally, the supernatant was centrifuged (4 °C, 14 000 rpm, 30 min) to obtain the CM.

The concentration of CM and RM membrane protein was quantified by BCA protein assay kit. The CM and RM were mixed at the protein mass ratio of 1:1 and sonicated in an ice bath for 10 min. To achieve the membrane fusion, the mixture solution was successively extruded through polycarbonate membranes (1 µm, 400, and 200 nm) for 11 times by Avanti Mini‐Extruder (Merck, Germany), and the attained CT26‐RBC hybrid membrane (CRM) was stored at −80 °C for further use.

SDS‐PAGE and Western blotting were employed to characterize the specific proteins of CRM, including Band 3 (from RM), E‐Cadherin (from CM), and Na, K‐ATPase (internal reference). To examine the membrane fusion, CM and RM were labeled with DiO (green) and DiD (red), respectively. The labeled cell membranes were sonicated and extruded as described above to form CRM. The membrane fusion was monitored by a fluorescence microscope (Olympus, Japan).

### Preparation and Characterization of PTSK@CRM Nanoparticles

First, PTS and KYNase were mixed at a mass ratio of 3.5:1 and sonicated in an ice bath for 30 min to prepare KYNase‐loaded nanoparticles (denoted by PTSK). Then, the CRM sna PTSK solutions were mixed together, sonicated, and extruded to obtain hybrid membrane CRM‐camouflaged PTSK nanoparticles (denoted by PTSK@CRM). Finally, PTSK@CRM was centrifugated (4 °C, 10 000 rpm, 5 min) to remove excess membrane, and resuspended in deionized water or PBS for future use.

The particle size and zeta potential of different nanoparticles were measured in deionized water by Malvern NanoZS90 Zetasizer (Malvern, UK) and analyzed by Malvern Zetasizer software 7.11. To assess the stability of nanoparticles, the hydrodynamic diameter and zeta potential of PTSK@CRM were measured through the incubation in PBS, 10% FBS‐containing, or 35% HS‐containing RPMI 1640 medium for 5 days. The hydrodynamic diameter of PTSK@CRM was also tested in different buffers (a) pH 7.4; b) pH 7.4 + 1 mm H_2_O_2_; c) pH 7.4 + 10 mm H_2_O_2_; d) pH 5 + 1 mm H_2_O_2_; e) pH 5 + 10 mm H_2_O_2_). The morphology of PTSK@CRM in deionized water was detected by TEM (JEM‐2100, FJEOL, Japan).

### In Vitro Drug Loading and Release

To calculate the EE and DLC, the content of Sim in the PTS solution (1 mL, 1 mg mL^−1^) was measured with a UV–vis spectrometer at 247 nm. Afterward, PTS (4.6 mg) and KYNase (1.3 mL, 1 mg mL^−1^) were mixed, sonicated, and centrifuged (4 °C, 10 000 rpm, 5 min). The supernatant was collected and detected by the BCA protein assay kit. The EE and DLC values were calculated as follows:

(1) $$EE\;\left(\%\right)=\frac{\mathrm{Weight}\;\mathrm{of}\;\mathrm{the}\;\mathrm{drug}\;\mathrm{loaded}\;\mathrm{in}\;\mathrm{nanoparticles}}{\mathrm{Weight}\;\mathrm{of}\;\mathrm{the}\;\mathrm{feeding}\;\mathrm{drug}}\;\times \;100\%$$

(2) $$DLC\;\left(\%\right)=\frac{\mathrm{Weight}\;\mathrm{of}\;\mathrm{the}\;\mathrm{drug}\;\mathrm{loaded}\;\mathrm{in}\;\mathrm{nanoparticles}}{\mathrm{Total}\;\mathrm{weight}\;\mathrm{of}\;\mathrm{nanoparticles}}\;\times \;100\%$$

Next, the in vitro release of Sim from PTS was measured. Briefly, PTS (2 mg) was dissolved in 2 mL of different PBS buffers (a. pH 7.4; b. pH 7.4 + 1 mm H_2_O_2_; c. pH 7.4 + 10 mm H_2_O_2_; d. pH 5 + 1 mm H_2_O_2_; e. pH 5 + 10 mm H_2_O_2_). After the stirring at 37 °C (220 rpm), 0.1 mL of released buffer was collected at predetermined timepoints, lyophilized, and resuspended in methanol followed by the filtration through 0.22‐µm filters. The concentration of Sim in filtrate was detected at 238 nm by LC‐20A HPLC (Shimadzu, Japan) equipped with a reverse‐phase C18 column, in which a mobile phase of methanol and water (60/40, v/v, 0.1% TFA) was used at a flow rate of 1 mL min^−1^.

To monitor the release behavior of KYNase, PTSK (1 mg on a KYNase basis) was dissolved in 1 mL of different PBS buffers (a. pH 7.4; b. pH 6.5; c. pH 5; d. pH 5 + 1 mm H_2_O_2_; e. pH 5 + 10 mm H_2_O_2_). After the centrifugation (4 °C, 10 000 rpm, 5 min) to collect the supernatant at predetermined timepoints, the concentration of KYNase in the supernatant was measured by the BCA protein assay kit.

### In Vitro Homotypic Targeting and Cellular Uptake

First, PTS and FITC were mixed at a molar ratio of 1:4, and the mixture was stirred overnight. FITC‐labeled PTS (FITC‐PTS) was obtained after dialysis and lyophilization. FITC‐PTS was used to encapsulate KYNase to prepare FITC‐PTSK, and FITC‐PTSK was coated with CRM to obtain FITC‐labeled PTSK@CRM (FITC‐PTSK@CRM). Afterward, CT26, NIH/3T3, or 4T1 cells were seeded in 6‐well plates (5 × 10^5^ cells per well) and cultured overnight, and 1 mL of fresh medium containing FITC‐PTSK or FITC‐PTSK@CRM (10 µg mL^−1^ on Sim basis) was added into each well. After the incubation for 4 h, the cells were washed with PBS three times, collected, and analyzed by the CytoFLEX flow cytometry system (Beckman Coulter, USA).

Further, CT26, NIH/3T3, or 4T1 cells were seeded onto glass coverslips placed in 6‐well plates (5 × 10^5^ cells per well) and cultured overnight. Then fresh medium containing FITC‐PTSK or FITC‐PTSK@CRM (10 µg mL^−1^ on Sim basis) was added, and the cells were incubated for another 4 h. The nucleus was stained with DAPI after washing the cells with PBS, and the samples were detected by FV4000 CLSM (Olympus, Japan). Similarly, CT26 cells were seeded onto glass coverslips placed in 6‐well plates and cultured overnight. Fresh medium (1 mL) containing FITC‐PTSK@CRM (10 µg mL^−1^ on Sim basis) was added into each well, and the cells were cultured for 2, 4, or 8 h. After washing with PBS, the cells were stained with Lyso‐Tracker Red (75 nm, 1 mL) for 30 min. Finally, the nucleus was stained with DAPI, and the samples were visualized under CLSM.

Similarly, KYNase was labeled with FITC at a molar ratio of 1:4 and stirred overnight to obtain FITC‐KYNase, which was then used to prepare FITC‐PTSK and FITC‐PTSK@CRM. To conclusively demonstrate the lysosomal escape ability, the lysosomal escape experiments of FITC‐KYNase, FITC‐PTSK, and FITC‐PTSK@CRM were conducted in CT26 cells.

### In Vitro Anti‐Tumor Ability—Cell Viability Assay

CT26 cells (8 × 10^3^ cells well^−1^, 100 µL well^−1^) were seeded in 96‐well plates and cultured at 37 °C for 12 h. Then, 100 µL of fresh medium with different concentrations of Sim, PTS, PTSK, PTS@CRM, PAMAM, or PTSK@CRM was added into the wells. After the incubation for 48 h, MTT solution (20 µL per well, 5 mg mL^−1^) was added, and the plates were kept at 37 °C for 4 h. The medium was removed, and 150 µL DMSO was added to dissolve the formazan crystals. The absorbance at 490 nm was measured by a microplate reader (BioTek Synergy LX, USA), and IC_50_ was analyzed through nonlinear regression by GraphPad Prism 9.5. The cell viability (%) was calculated through the following formula:

(3) $$Cellviability\left(\%\right)=\;\;\frac{A_{e\;}-A_{b}}{A_{c}-A_{b}}\times 100\%$$

where *A*_e_, *A*_c_, and *A*_b_ represented the absorbance of the experimental well, control well, and blank well, respectively.

### In Vitro Anti‐Tumor Ability—Apoptosis Assay

CT26 cells were inoculated and cultured in 6‐well plates, and then 1 mL of fresh medium containing Sim, PTS, PTSK, PTS@CRM, or PTSK@CRM (5 µg mL^−1^ on Sim basis) was added into each well. After the incubation for 48 h, the cells were collected through centrifugation (4 °C, 2500 rpm, 5 min), and stained with Annexin V‐FITC and PI according to the manufacturer's instructions. The ratio of apoptotic cells was measured by the CytoFLEX flow cytometry system (Beckman Coulter, USA).

### In Vitro Anti‐Tumor Ability—Caspase 3 Assay

CT26 cells were inoculated and cultured as described above. The collected cells were lysed using RIPA buffer for Western blotting assay to evaluate the expression of Caspase 3. Additionally, the activity of Caspase 3 was analyzed using the Caspase‐Glo 3/7 activity assay kit.

### In Vitro Reprogramming Pathways

CT26 cells were seeded into 6‐well plates (3 × 10^5^ cells per well) and cultured overnight. Then, the cells were stimulated with IFN‐γ (100 ng mL^−1^) for 12 h, and 1 mL of fresh medium containing Sim, PTS, PTSK, PTS@CRM, or PTSK@CRM (10 µg mL^−1^ on Sim basis) was added into each well. After the incubation at 24 h, the cells were collected through centrifugation (4 °C, 3000 rpm, 10 min).

### Cholesterol Assay

The collected CT26 cells were homogenized in chloroform–methanol (2/1, v/v, 200 µL) and centrifuged (4 °C, 14 000 rpm, 10 min). The organic phase was collected, and chloroform and methanol were removed in vacuum. According to the instructions of the Amplex Red Cholesterol Assay Kit, the content of cholesterol was monitored by a microplate reader.

### Kyn and Trp Measurement

First, the collected CT26 cells were subjected to the extraction with methanol/acetonitrile (1/1, v/v, 400 µL), and the level of Kyn and Trp was detected by LC‐MS, Infinity UPLC, Agilent; QTRAP 6500, SCIEX, USA) after the centrifugation at 4 °C (14 000 rpm, 15 min).

Eventually, the expression of PD‐L1 in CT26 cells was evaluated after different treatments by Western blotting, and the IF of AhR and Rab5 was conducted using CLSM to analyze the translocation and expression of AhR and Rab5 in the treated CT26 cells.

### Hemolysis Assay

RBCs were collected and diluted with PBS to obtain the cell suspension (4%, v/v). The cell suspension and PTSK (or PTSK@CRM) at different concentrations (62.5, 125, 250, and 500 µg mL^−1^ on PTS basis) were mixed at the same volume, and the samples were incubated at 37 °C for 1 h. The mixture was centrifuged (4 °C, 3500 rpm, 10 min) to photograph, and the supernatant was added to a 96‐well plate (100 µL well^−1^) to measure the absorbance at 570 nm by a microplate reader. Triton X‐100 and saline was employed as the positive or negative control, respectively. The hemolysis rate was calculated by the following formula:

(4) $$Hemolysis\left(\%\right)=\frac{A_{e}-A_{nc}}{A_{pc}-A_{nc}}\times 100\%$$

where *A*_e_, *A*_nc_, and *A*_pc_ represented the absorbance of the experimental group, the negative control, and the positive control, respectively.

### In Vivo Biodistribution Analysis

The PTS and Cy5‐SE were reacted at a molar ratio of 1:3 overnight to prepare the Cy5‐PTS. Cy5‐PTS was then used to encapsulate KYNase to construct Cy5‐PTSK, and Cy5‐PTSK was coated with CRM to obtain Cy5‐PTSK@CRM. When the tumor volume reached ≈200 mm^3^, the CT26 tumor‐bearing BALB/c mice were randomly divided into three groups: Saline, Cy5‐PTSK, and Cy5‐PTSK@CRM (10 mg kg^−1^ on Sim basis, 100 µL). The saline, Cy5‐PTSK, or Cy5‐PTSK@CRM was intravenously (*i.v*.) injected via the tail vein, and the mice were sacrificed at predetermined time points (1, 4, and 24 h, *n *= 3 per time point). The biodistribution of nanoparticles in excised organs (heart, liver, spleen, lungs, kidneys) and tumors was visualized by IVIS Lumina XR Spectrum system (PerkinElmer, Hopkinton, MA).

### In Vivo Pharmacokinetic Analysis of PTSK@CRM

To investigate the pharmacokinetics of KYNase, Cy5‐KYNase, Cy5‐PTSK, and Cy5‐PTSK@CRM were injected into SD rats via intravenous administration (20 mg kg^−1^ on the basis of KYNase). At different time points (0.03, 0.08, 0.17, 0.25, 0.5, 1, 2, 4, 6, 8, and 12 h) after injection, 0.2 mL of blood samples were collected by the retro‐orbital puncture into pre‐anticoagulated tubes. Then the blood was centrifuged (3000 rpm, 10 min, 4 °C) to collect serum. The fluorescence intensity of samples was detected on a microplate reader (BioTek Synergy LX, USA), with excitation and emission wavelengths of 640 and 680 nm, respectively.

Afterward, to evaluate the pharmacokinetics of Sim, PTSK, and PTSK@CRM were injected into SD rats via intravenous administration (10 mg kg^−1^ on the basis of Sim). Blood samples (0.2 mL) were collected at different time points (0.03, 0.08, 0.17, 0.25, 0.5, 1, 2, 4, 6, 8, and 12 h) after injection, and then centrifuged as described above to collect the serum. H_2_O_2_ (0.1 m) was added into the serum samples, and the mixture was extracted by 0.5 mL of cyclohexane/chloroform (3.5:1, v/v). The upper layer was collected and dried using a nitrogen blow dryer (Ruicheng, Hangzhou, China), and then dissolved in 50 µL of acetonitrile. The concentration of Sim was detected at 238 nm by HPLC (a reverse‐phase C18 column, mobile phase: acetonitrile/water, 70/30, v/v). The concentration–time curve was plotted, and the pharmacokinetic parameters were analyzed using DAS2.0 software (Shanghai, China).

### In Vivo Evaluation of Antitumor Efficacy

The CT26 tumor‐bearing mice models were established by subcutaneously implanting 1 × 10^6^ CT26 cells into the right flank of female BALB/c mice. When the tumor volume reached ≈100 mm^3^, the CT26 tumor‐bearing BALB/c mice were randomly divided into eight groups (*n* = 5): 1) Saline, 2) Sim, 3) PTS, 4) PTSK, 5) PTS@CRM, 6) PTSK@CRM, 7) αPD‐1, 8) αPD‐1 + PTSK@CRM (10 mg kg^−1^ on Sim basis; 20 mg kg^−1^ on KYNase basis; 10 mg kg^−1^ on αPD‐1 basis). The nanoparticles (100 µL) were intravenously (*i.v*.) injected on Days 0, 2, 4, and 6. Sim and αPD‐1 were intraperitoneally (*i.p*.) injected on Days 0, 2, and 4. The tumor volume and body weight were measured every day. On Day 14, the mice were sacrificed, and tumors were collected, photographed, and weighed. The tumors were also fixed and cut into slices for H&E, Ki‐67 (IHC), TUNEL, PD‐L1 (IF), or AhR (IF) staining. The quantitative analysis of immunohistochemistry and immunofluorescence was performed using Image J (v1.5j8, National Institutes of Health, Bethesda, MD) with threshold‐based segmentation. The tumor volume (mm^3^) was calculated using the following equation:

(5) $$\mathrm{Tumor}\;\mathrm{volume}\;\left(mm^{3}\right)=\frac{\left(Length\times width^{2}\right)}{2}$$

The CT26 tumor metastasis models were conducted by the intravenously injection of 2.5 × 10^5^ CT26 cells into the tail vein of female BALB/c mice. The mice were randomly divided into three groups (*n* = 5): 1) Saline, 2) PTSK, and 3) PTSK@CRM. Hundred microliters of saline, PTSK or PTSK@CRM (10 mg kg^−1^ on Sim basis; 20 mg kg^−1^ on KYNase basis) were intravenously injected on Day 0, 2, 4, and 6. Body weight was measured every other day. On Day 14, the lungs were collected and photographed to observe the metastatic nodules.

The MC38 tumor‐bearing mice models were constructed by subcutaneously implanting 1 × 10^6^ MC38 cells into the right flank of female C57BL/6 mice. When the tumor volume reached ≈50 mm^3^, the MC38 tumor‐bearing mice were randomly divided into three groups (*n* = 4): 1) Saline, 2) PTSK, and 3) PTSK@CRM. 100 µL of saline, PTSK, or PTSK@CRM (10 mg kg^−1^ on Sim basis; 20 mg kg^−1^ on KYNase basis) were intravenously injected on Days 0, 2, 4, 6, and 8. The tumor volume and body weight were measured every other day. On Day 20, the mice were sacrificed, and tumors were collected, photographed, and weighed.

### In Vivo Metabolite Measurement—Metabolite Measurement

The tissues and serum were homogenized in methanol/water (80/20, v/v, 400 µL) and centrifuged (12 000 rpm, 10 min, 4 °C). The supernatants were subjected to LC‐MS/MS to monitor the levels of Trp and mevalonate metabolites.

In Vivo Metabolite Measurement—Cholesterol Assay

In brief, the tumor tissue (10 mg) and blood serum were homogenized in 200 µL of chloroform/methanol (2/1, v/v). After the centrifugation at 4 °C (14 000 rpm, 10 min), the organic phase was desiccated in a vacuum, and the content of cholesterol was measured according to the instructions of the Amplex Red Cholesterol Assay Kit as described above.

### In Vivo Analysis of Immune Cells and Cytokines—Immune Cell Analysis

On Day 14, the tumors of CT26 tumor‐bearing mice were digested, and the spleens and TDLNs were triturated. Then the mixture was filtrated through a 200‐mesh nylon filter and centrifuged (4 °C, 1800 rpm, 5 min). The cell pellets were resuspended in fluorescence activated cell sorting buffer (FACS) to obtain single‐cell suspensions. Afterward, the single cell suspensions were incubated with respective fluorescent antibodies for 30 min and washed with FACS buffer. The percentages of immune cells were assessed using flow cytometry (BD LSRFortessa, USA), including cytotoxic T cells (CD45^+^CD3^+^CD8^+^), helper T cells (CD45^+^CD3^+^CD4^+^), regulatory T cells (Tregs, CD45^+^CD3^+^CD4^+^CD25^+^Foxp3^+^), M1‐like macrophages (CD45^+^F4/80^+^CD11b^+^CD86^+^), M2‐like macrophages (CD45^+^F4/80^+^CD11b^+^CD206^+^), MDSCs (CD45^+^CD11b^+^Gr‐1^+^), and DCs (CD45^+^CD11c^+^CD80^+^CD86^+^). Moreover, IF staining (CD4, CD8, iNOS and CD206) of tumor tissues was conducted

### In Vivo Analysis of Immune Cells and Cytokines—Cytokines Measurement

The levels of IL‐2, IL‐6, IL‐10, TNF‐α, and IFN‐γ in tumor tissues and serum were detected by ELISA kits according to the manufacturers’ instructions.

### In Vivo Biosafety Evaluation

In order to evaluate in vivo biosafety, major organs (heart, liver, spleen, lungs, and kidneys) collected at 0, 1, 4, 24 h, and Day 14, were fixed with 4% paraformaldehyde, and the paraffin sections were subjected to H&E staining. Meanwhile, the serum samples were collected to detect the biochemical indicators, including ALT, AST, CRE, BUN, and ALP.

To evaluate the potential immunogenicity of PTSK@CRM, a repeated administration study was conducted in healthy female BALB/c mice (6–8 weeks old). The mice received intravenous injection of PTSK@CRM every other day for 4 times. Afterward, the blood samples were collected and centrifuged (3000 rpm, 10 min, 4 °C) to obtain the serum. Levels of IgM and IgG antibodies in the serum were measured using the corresponding ELISA kits.

### CyTOF

To comprehensively characterize the immune landscape within CT26 lung metastatic tumors, single‐cell suspensions were prepared from freshly harvested tissues and analyzed by CyTOF. Briefly, metastatic nodules from the lungs were collected and dissociated. The tumors were then digested at 37 °C for 30 min to obtain a single‐cell suspension, which was then Live/Dead stained with 2 µm cisplatin for 2 min before quenching with cell staining buffer (CSB). Cells were then fixed with Fix‐I Buffer for 15 min and washed with PBS three times. To minimize internal cross reaction, the cells were labeled using Cell‐ID 20‐Plex Pd Barcoding Kit. Antibodies were conjugated using MaxPar X8 Polymer Kits according to the manufacturer's instructions, and all metal‐conjugated antibodies were pre‐titrated to determine the optimal staining concentrations. Single‐cell suspension (1 × 10⁶ cells mL^−1^) was incubated with a surface antibody mixture in 50 µL CSB for 30 min at room temperature. Afterward, the cells were permeabilized with 80% methanol for 15 min on ice and subsequently stained with the intracellular antibodies for 30 min. After washing with CSB three times, the cells were incubated at 4 °C overnight with 0.125 µm iridium nucleic acid intercalator in fix and perm buffer. After the intercalator staining, the cells were washed with cold PBS three times and resuspended in deionized water containing 10% EQ four‐element calibration beads. Data were acquired on Starion M 1.0 mass cytometer (Polaris, Shanghai, China), and raw data were normalized to generate.fcs files for each sample. All.fcs files were subsequently uploaded to the Cytobank platform (https://premium.cytobank.org/cytobank/) for data processing, including the identification of cell populations via unsupervised clustering and t‐SNE.

### Cytokine Recall Assay

PBMCs were isolated from 1 mL of whole blood collected in anticoagulant tubes from CT26 lung metastasis mice. Briefly, the blood was diluted with an equal volume of PBS and carefully layered over a Ficoll Plus density gradient medium. Following the centrifugation at 400 *g* for 30 min at room temperature, the PBMC layer at the interface was aspirated. The harvested PBMCs were then washed with PBS twice and resuspended in 10% FBS‐harboring RPMI 1640 medium. The cells were subsequently seeded in 96‐well plates at a density of 2 × 10^4^ cells well^−1^ and re‐stimulated with CT26 or MC38 tumor cell lysate (20 µg mL^−1^). The cells were incubated in 10% FBS‐harboring RPMI 1640 medium for 6 or 24 h at 37 °C with 5% CO_2_, and then the culture supernatants were carefully collected. The concentrations of IL‐2, TNF‐α, and IFN‐γ in the supernatants were quantified using the corresponding ELISA kits.

### Statistical Analysis

Data were expressed as mean value ± standard deviation (SD). A Student's *t*‐test (unpaired, two‐tailed) was employed in the comparison of different data types between two groups. One‐way analysis of variance (ANOVA) with Tukey's post hoc test and two‐way ANOVA analysis with Bonferroni post hoc test were performed to determine the statistical significance of data between multiple groups. Spearman's correlation was employed to assess the correlation between two continuous variables, and *R* > 0.5 was considered correlated. The sample size for each study was provided in the Figure legends. The GraphPad Prism software (version 9.5) was used for all the statistical analysis. Values of *P* < 0.05 were considered statistically significant (n.s., no significance; * *P* < 0.05, ** *P* < 0.01, *** *P* < 0.001).

## Conflict of Interest

The authors declare no conflict of interest.

## Supporting information



Supporting Information

## Data Availability

The data that support the findings of this study are available from the corresponding author upon reasonable request.
