# Supplementary material for: Regulating Tumor Metabolic Reprogramming with Biomimetic Co‐Delivery of Simvastatin and Kynureninase for Immunotherapy
Source: Adv Sci (Weinh). 2025 Dec 23;13(12):e08107. doi: 10.1002/advs.202508107 (PMC12948188; doi:10.1002/advs.202508107)
Supplement: Supplementary file 1 — Supporting Information [file ADVS-13-e08107-s001.docx]

Supporting Information

**Regulating Tumor Metabolic Reprogramming with Biomimetic Co-delivery of Simvastatin and Kynureninase for Immunotherapy**

Jiaxin Yin ^a,b^, Shengcai Yang ^b^*, Zengguang Liu ^a,b^, Songchen Zhao ^a,b^, Siyu Sun ^b^, Ziling Liu ^a^* and Quanshun Li ^b,c^*

^a^Department of Cancer Center, The First Hospital of Jilin University, Changchun 130012, China

^b^Key Laboratory for Molecular Enzymology and Engineering of Ministry of Education, School of Life Sciences, Jilin University, Changchun 130012, China

^c^China-Singapore Belt and Road Joint Laboratory on Liver Disease Research, The First Hospital of Jilin University, Changchun 130012, China

E-mail: [yang_shengcai@jlu.edu.cn](mailto:yang_shengcai@jlu.edu.cn) (S. Yang); [ziling@jlu.edu.cn](mailto:ziling@jlu.edu.cn) (Z. Liu); [quanshun@jlu.edu.cn](mailto:quanshun@jlu.edu.cn) (Q. Li).


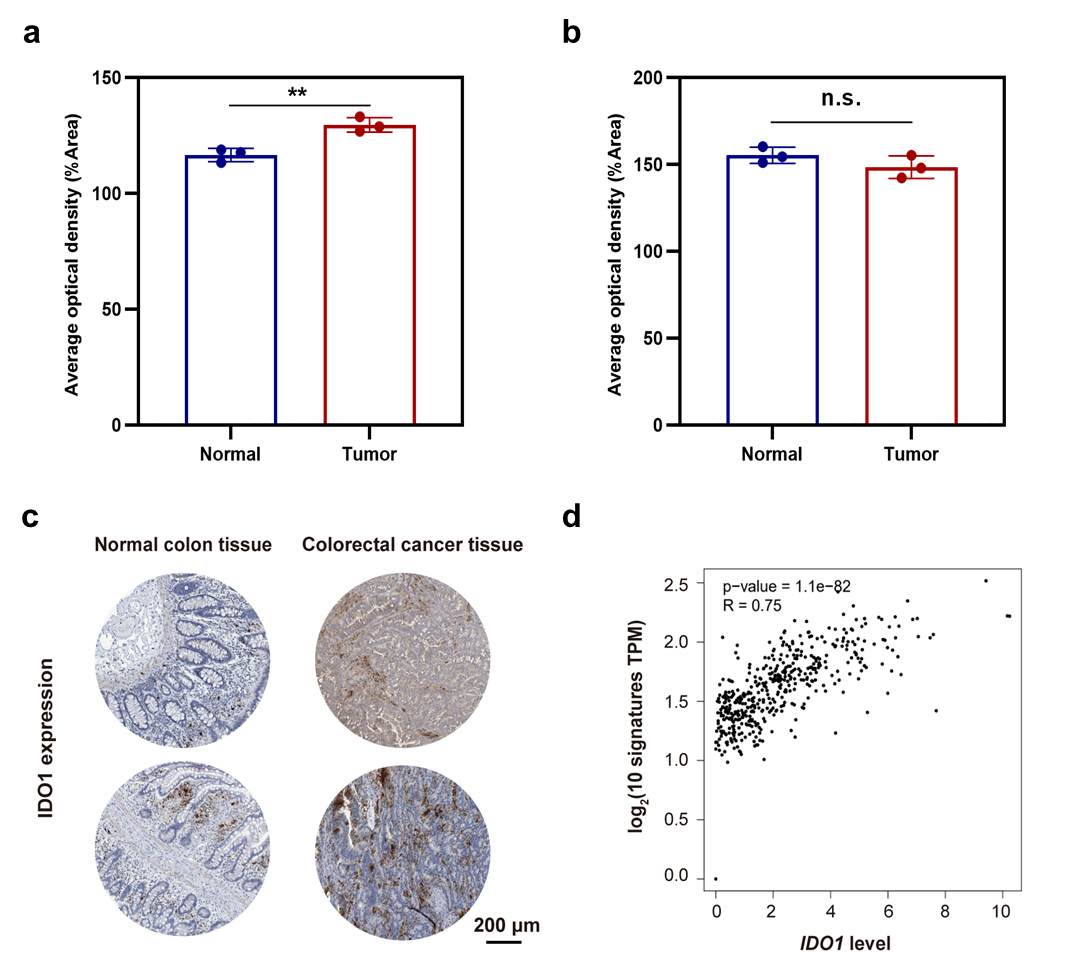


**Figure S1.** The average optical density of **a**) HMGCR and **b**) KYNU protein in IHC images between normal and tumor tissues. Data were presented as mean value ± SD (n = 3, unpaired and two-tailed t test, *******P* < 0.01, n.s., no significance). **c**) The protein expression of IDO1 in colorectal cancer and normal colon tissues from HPA database (scale bar = 200 μm). **d**) The spearman correlation analysis between the IDO1 mRNA level and the signature levels of immune cells (effector T cells and Th cells) in COAD tissues from GEPIA database.


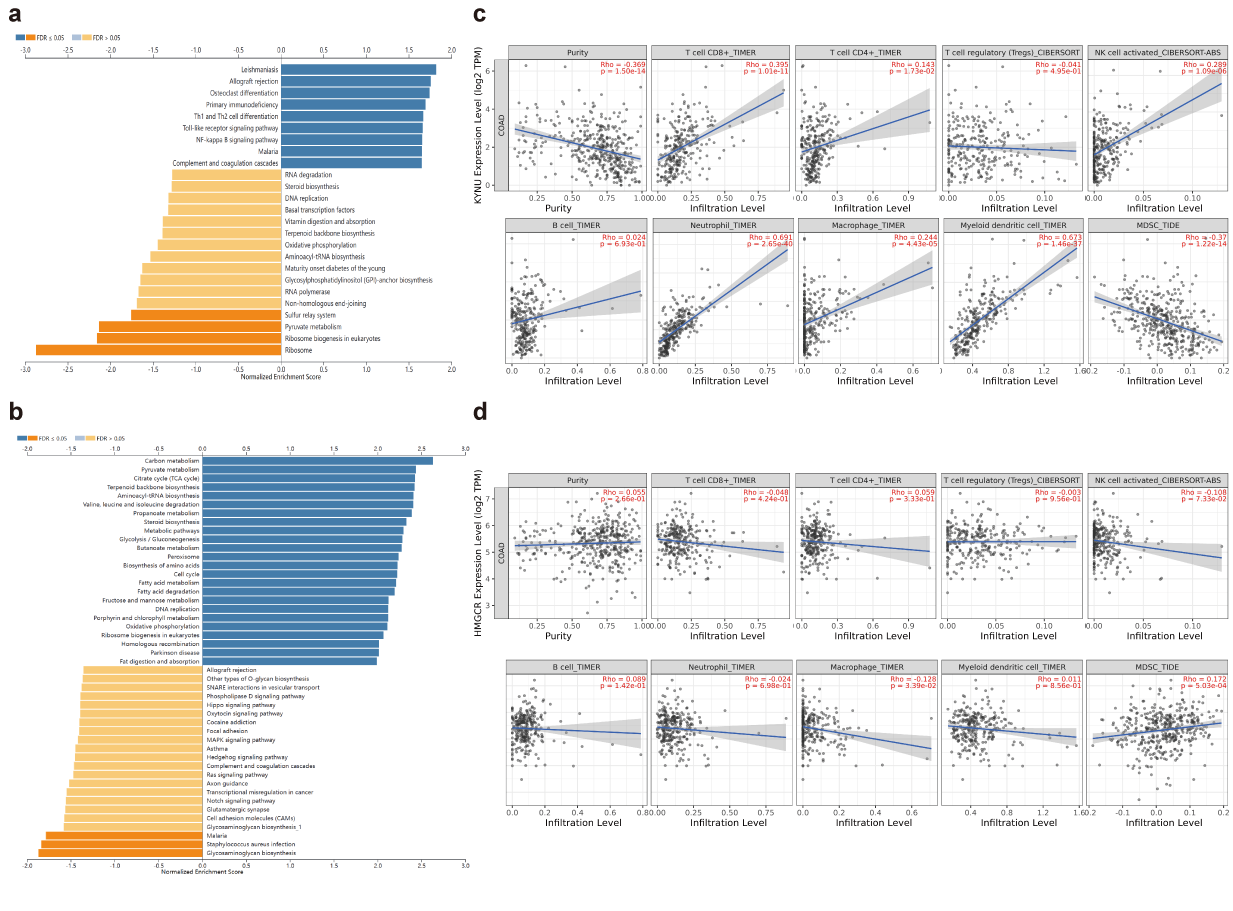


**Figure S2.** **a**) KEGG enrichment analysis of *KYNU* mRNA levels in COAD patients from Linkedomics database. **b**) The correlation between the infiltration of immune cells and the *KYNU* gene expression in COAD patients from TIMER database. **c**) KEGG enrichment analysis of *HMGCR* mRNA levels in COAD patients from Linkedomics database. **d**) The correlation between the infiltration of immune cells and the expression of *HMGCR* gene in COAD patients from TIMER database.


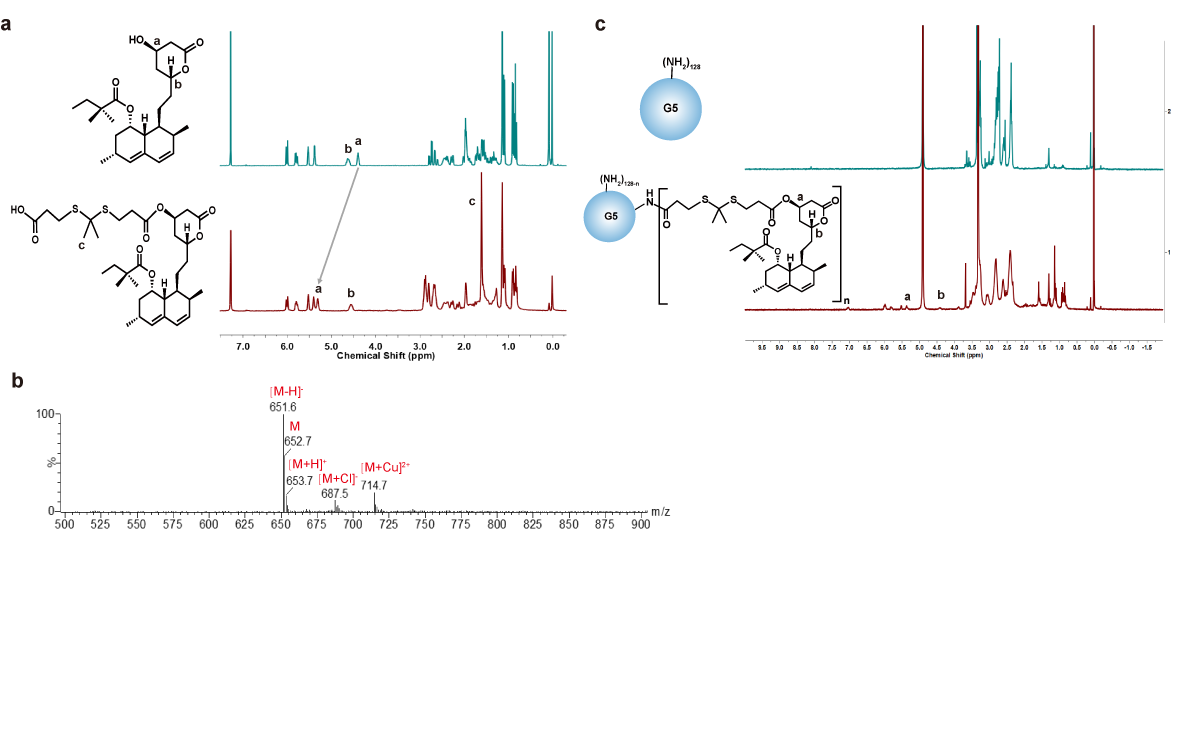


**Figure S3.** **a**) ^1^H NMR spectra of Sim and TK-Sim. **b**) The ESI-Q-TOF MS of TK-Sim. **c**) ^1^H NMR spectra of PAMAM and PTS.


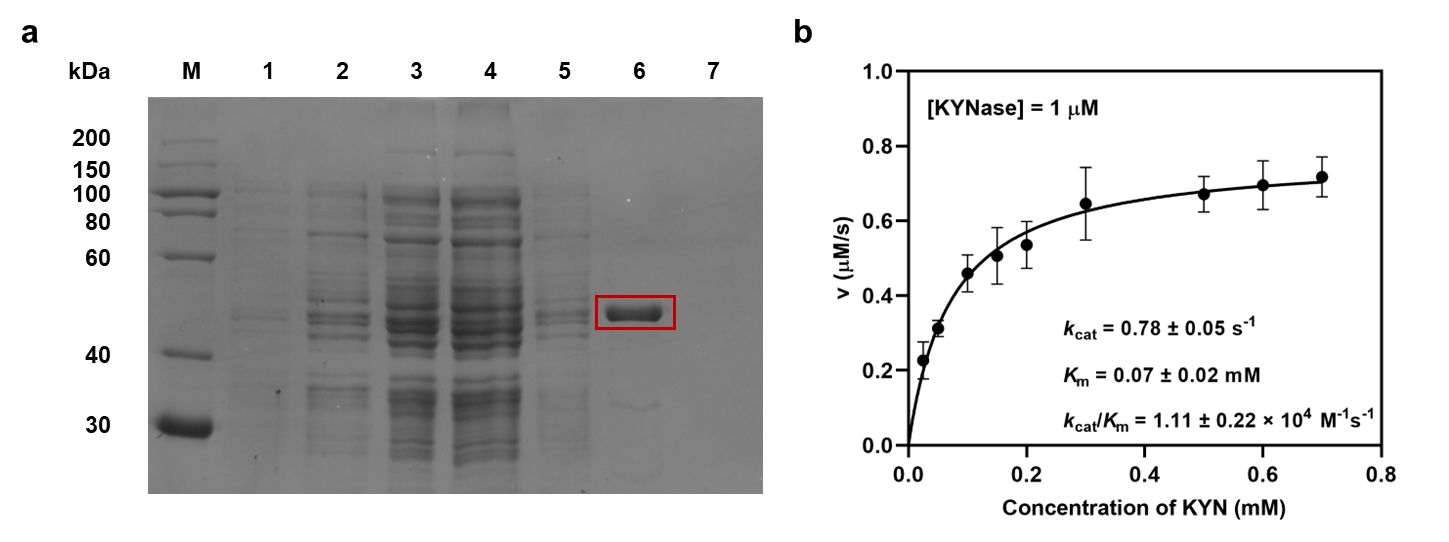


**Figure S4.** **a**) SDS-PAGE analysis of KYNase (lane M: marker; lane 1: the whole bacteria (uninduced with IPTG); lane 2: the whole bacteria (induced with IPTG); lane 3: the supernatant of bacteria lysate; lane 4: the effluent solution of loading sample; lane 5: the eluent with 50 mM imidazole; lane 6: the eluent with 250 mM imidazole; lane 7: the eluent with 500 mM imidazole). **b**) Michaelis-Menten kinetic analysis of KYNase using different concentration of Kyn as the substrates (n =3, nonlinear regression).


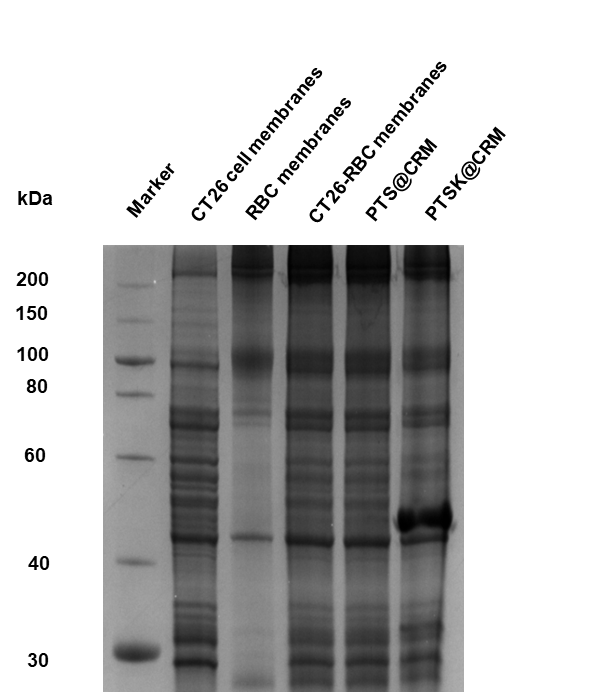


**Figure S5.** SDS-PAGE protein analysis of PTSK@CRM.


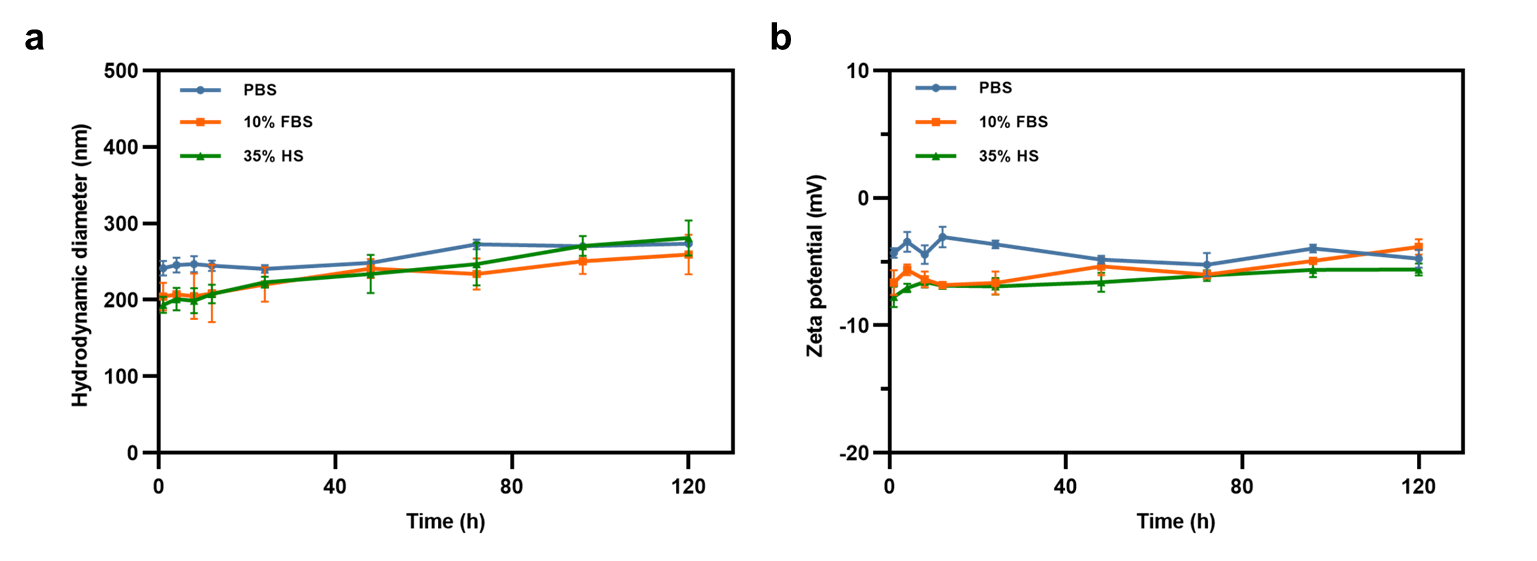


**Figure S6.** **a**) Hydrodynamic diameter and **b**) zeta potential of PTSK@CRM during the incubation in PBS, 10% FBS-containing or 35% HS-containing RPMI 1640 medium for 5 days. Data were presented as mean value ± SD (n =3).


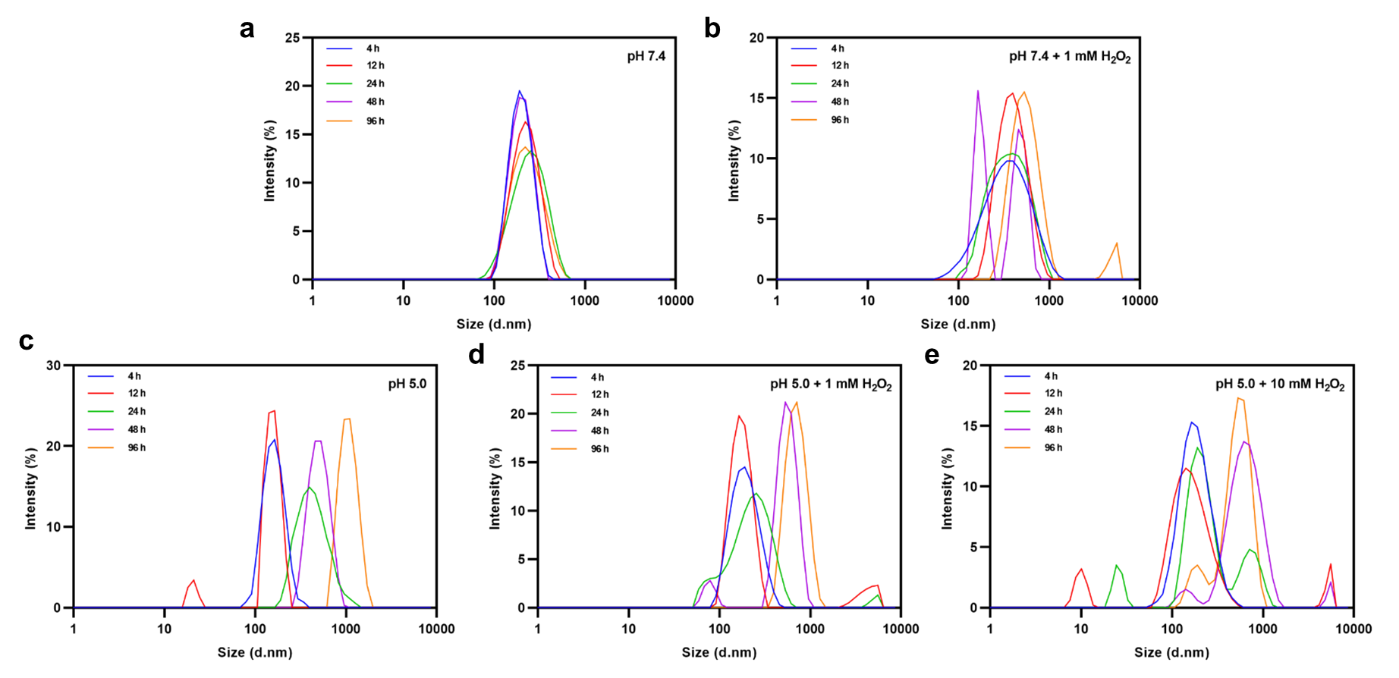


**Figure S7.** Hydrodynamic diameter values of PTSK@CRM in different buffers for various time. **a**) pH 7.4; **b**) pH 7.4 + 1 mM H_2_O_2_; **c**) pH 5.0; **d**) pH 5.0 + 1 mM H_2_O_2_; **e**) pH 5.0 + 10 mM H_2_O_2_.

**
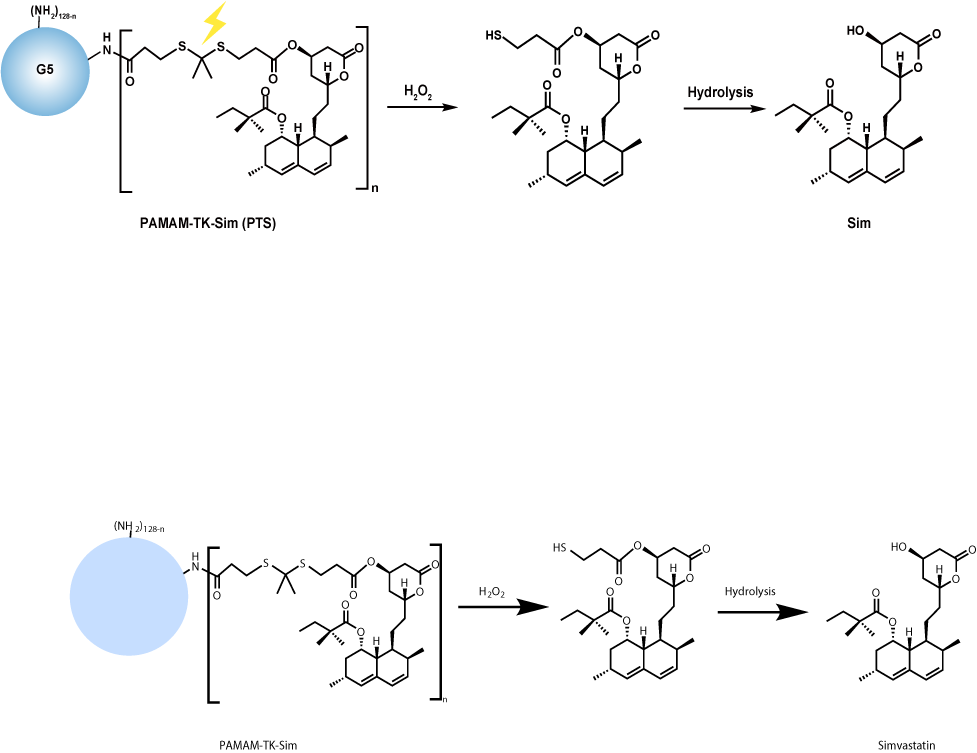
**

**Figure S8.** A schematic process of Sim release from PTS in H_2_O_2_ buffer.


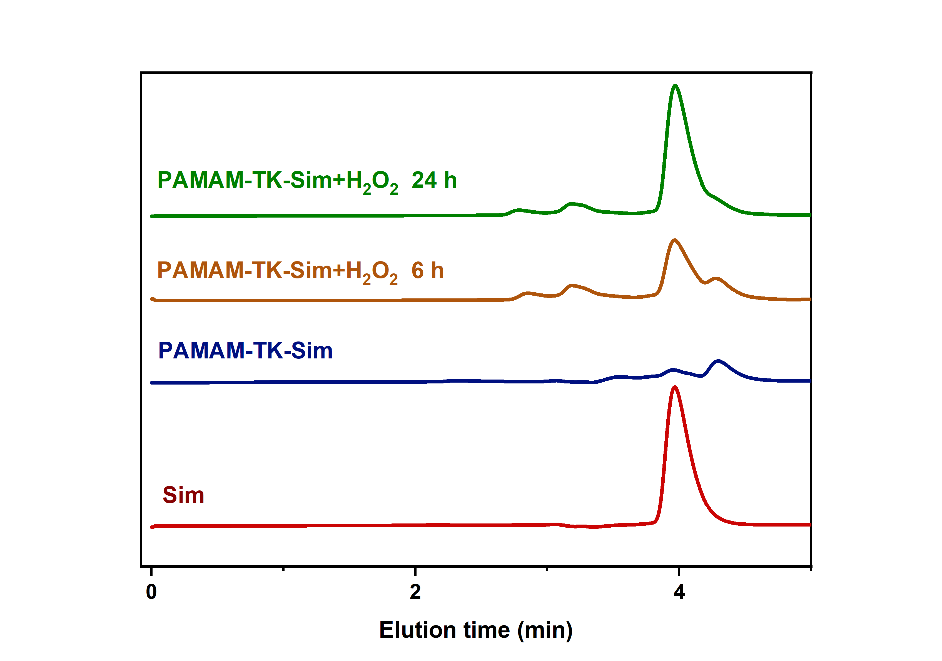


**Figure S9.** Qualitative analysis of Sim release in the presence or absence of H_2_O_2_ detected by HPLC at 238 nm.


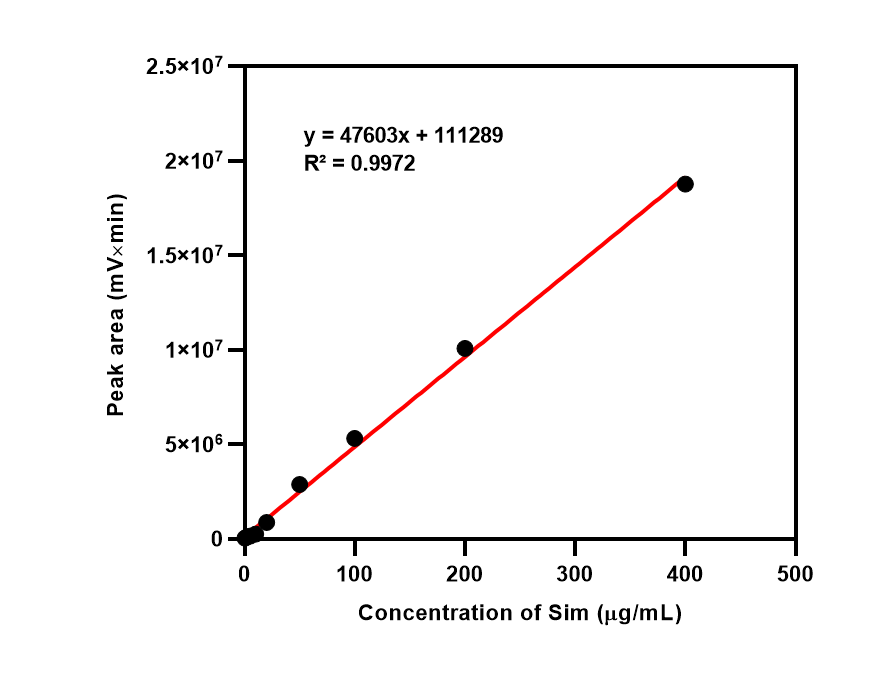


**Figure S10.** The standard curve of Sim detected by HPLC in the mobile phase (methanol/water, 6/4, v/v).


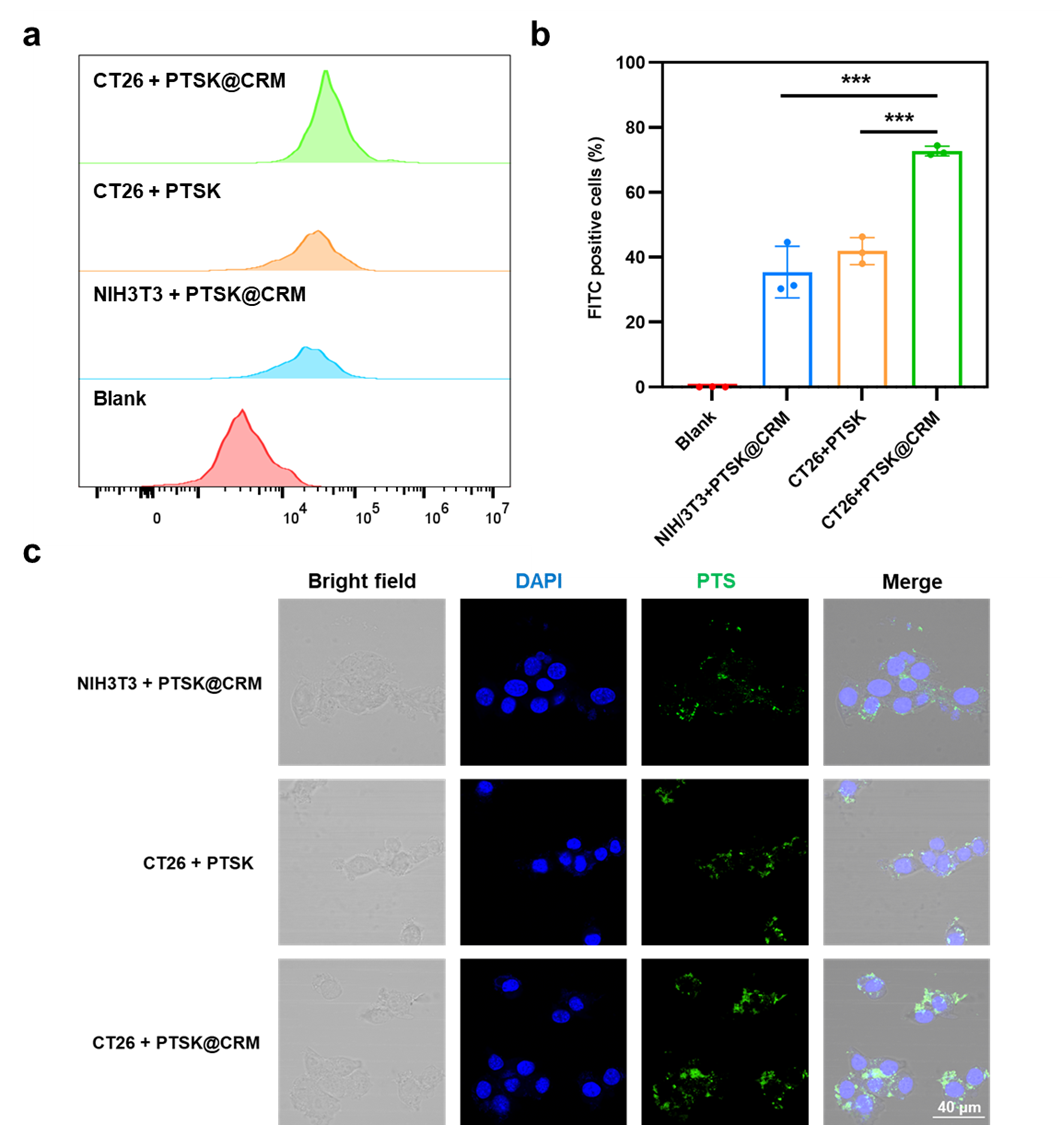


**Figure S11.** **a**) Flow cytometry and **b**) quantitative analysis of CT26 and NIH/3T3 cells after the incubation with FITC-PTSK or FITC-PTSK@CRM for 4 h (100 μg/mL on basis of PTS). Data were presented as mean value ± SD (n = 3, one-way ANOVA analysis, ********P* < 0.001). **c**) CLSM images of CT26 and NIH/3T3 cells after the incubation with FITC-PTSK or FITC-PTSK@CRM for 4 h (100 μg/mL on basis of PTS, green: FITC-PTS, blue: DAPI-nucleus, scale bar = 40 μm).


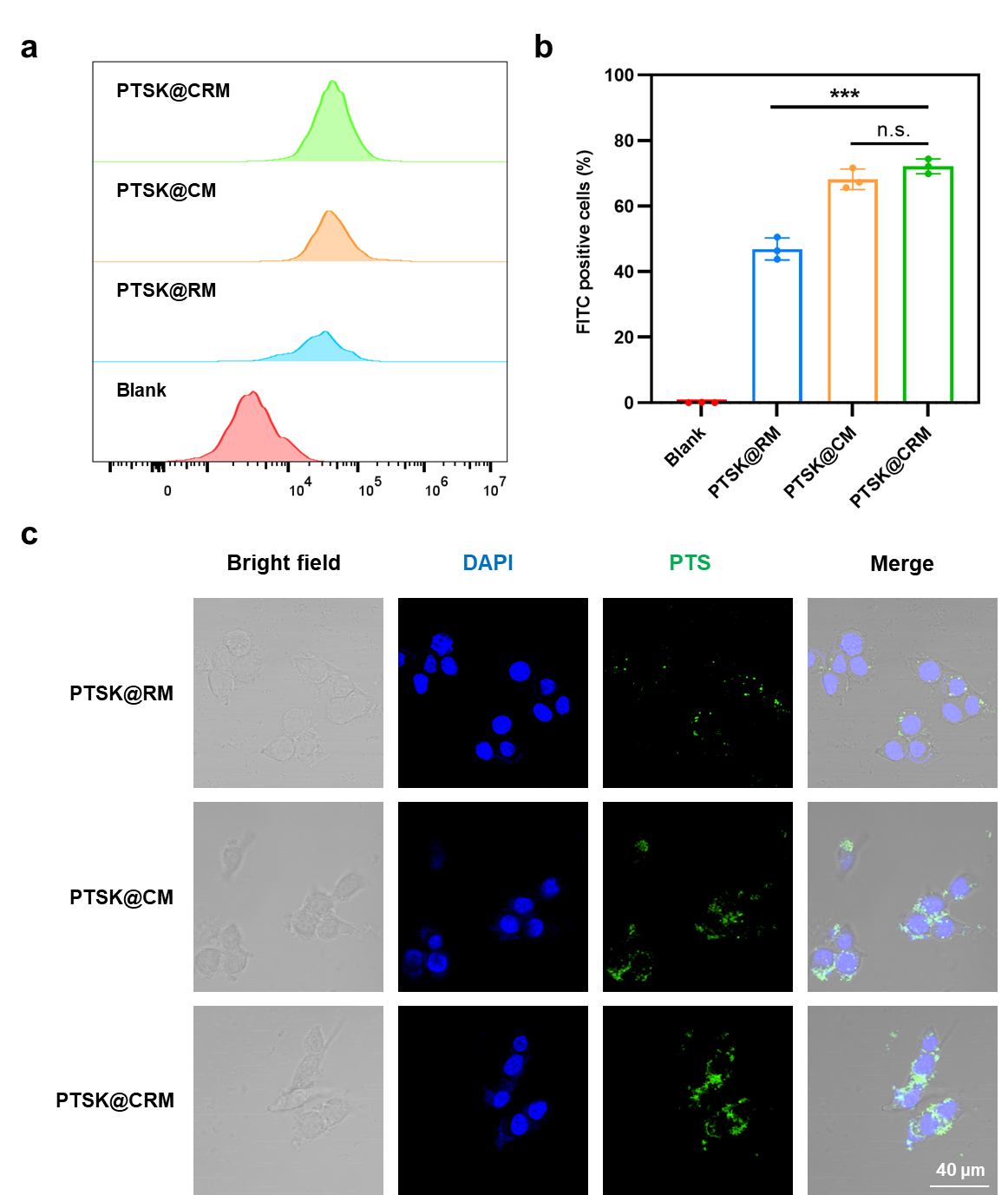


**Figure S12. a**) Flow cytometry and **b**) quantitative analysis of CT26 cells after the incubation with PTSK@CM, PTSK@RM and PTSK@CRM for 4 h (100 μg/mL on basis of PTS). Data were presented as mean value ± SD (n = 3, one-way ANOVA analysis, ********P* < 0.001, n. s., not significant). **c**) CLSM images of CT26 cells after the incubation with PTSK@CM, PTSK@RM and PTSK@CRM for 4 h (100 μg/mL on basis of PTS, green: FITC-PTS, blue: DAPI-nucleus, scale bar = 40 μm).

**
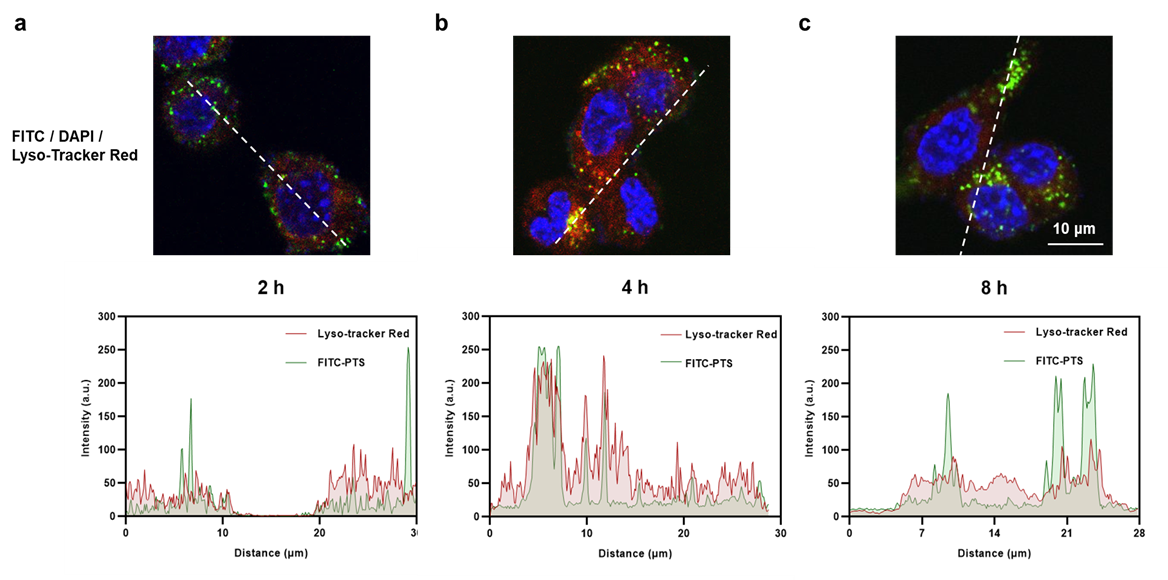
**

**Figure S13.** Intracellular biodistribution and fluorescence intensity of Lyso-Tracker Red and FITC in CT26 cells after the incubation with PTSK@CRM for **a)** 2, **b)** 4 and **c)** 12 h, respectively. (Green: FITC-PTS, blue: DAPI-nucleus, red: Lyso-Tracker Red, scale bar = 10 μm).


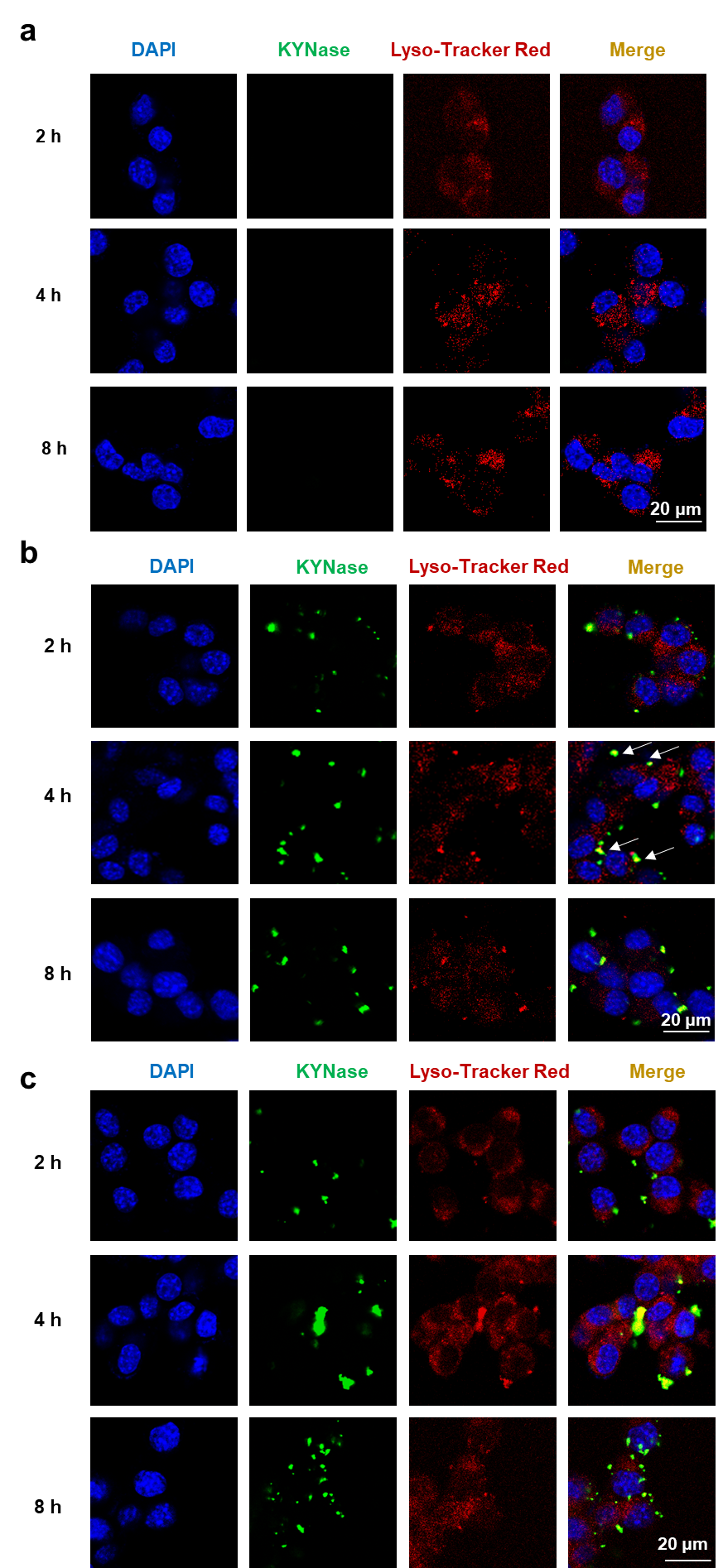


**Figure S14.** CLSM images for the lysosomal escape of **a**) free FITC-KYNase, **b**) FITC-PTSK and **c**) FITC-PTSK@CRM (Green: FITC-KYNase, blue: DAPI-nucleus, red: Lyso-Tracker Red, scale bar = 20 μm).


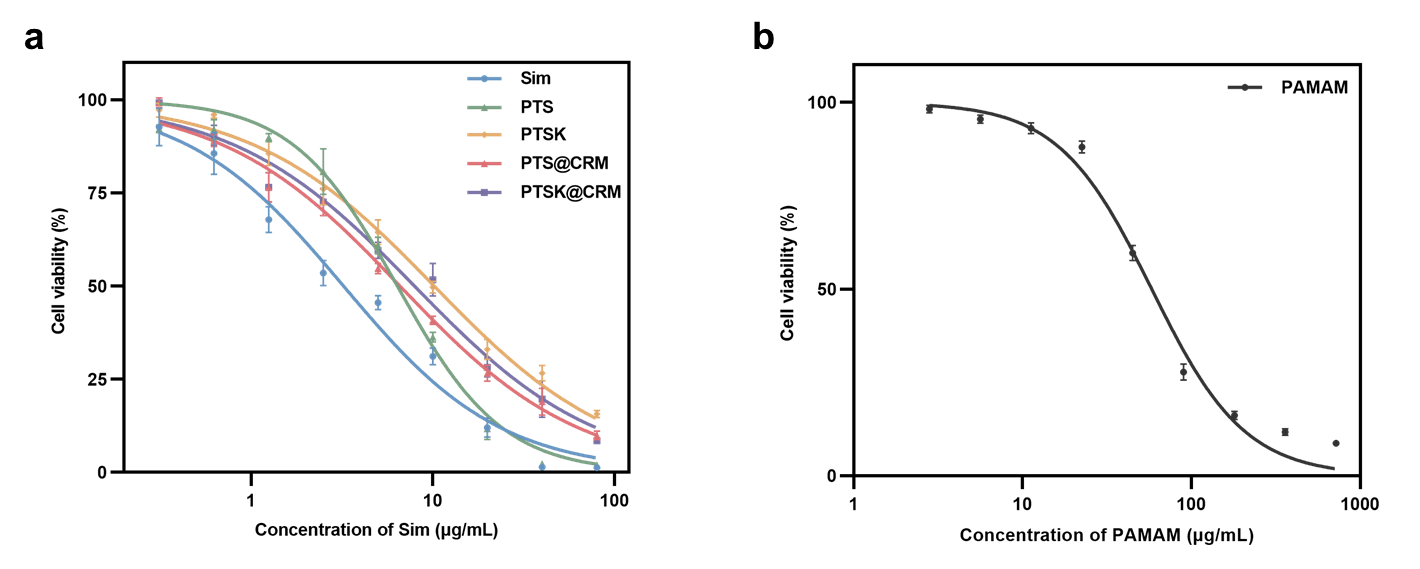


**Figure S15.** Cell viability of CT26 cells after the incubation with different concentrations of **a**) Sim, PTS, PTSK, PTS@CRM, PTSK@CRM or **b**) PAMAM. Data were presented as mean value ± SD (n =3, nonlinear regression) and were normalized to pre-process.


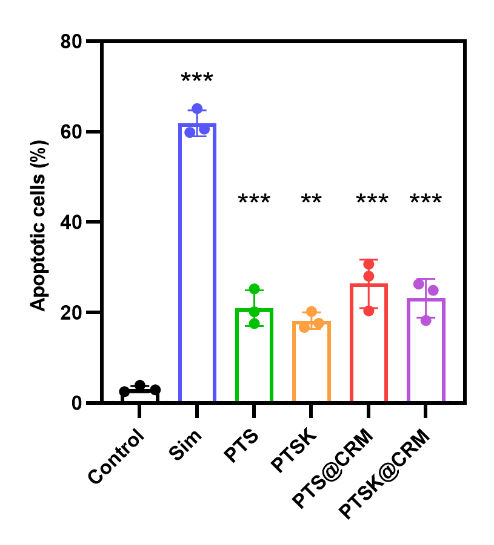


**Figure S16.** Quantitative analysis of apoptotic cells with different treatments for 48 h (n = 3). Annexin V-FITC^Low^PI^Low^: live cells; Annexin V-FITC^High^PI^Low^: early apoptotic cells; Annexin V-FITC^High^PI^High^: late apoptotic cells; Annexin V-FITC^Low^PI^High^: cell debris as well as dead cells. Data were presented as mean value ± SD (n = 3, *******P* < 0.01 *vs.* Control group, ********P* < 0.001 *vs.* Control group).

**
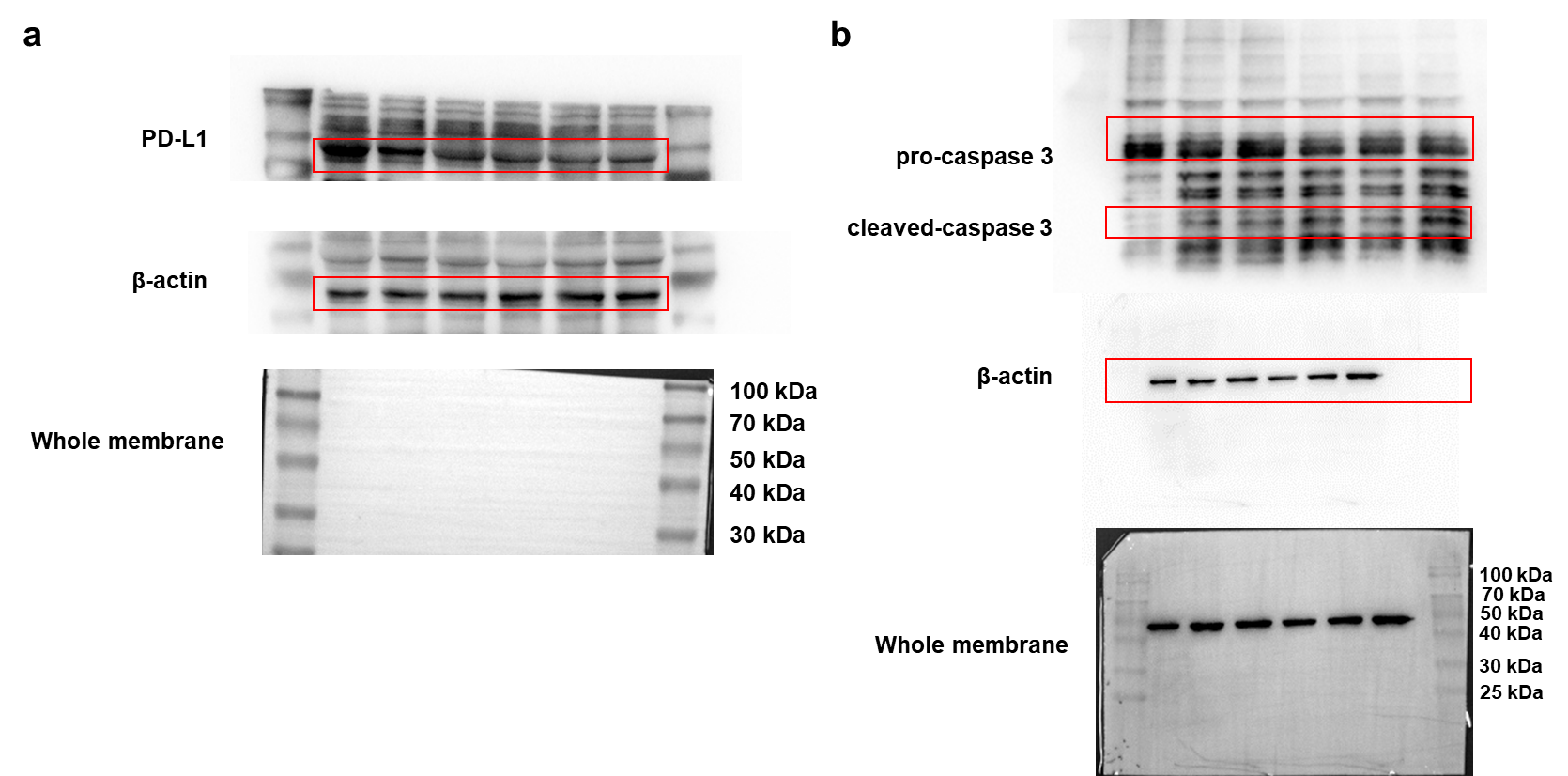
**

**Figure S17.** The original data for Western blotting analysis of **a**) PD-L1 and **b**) caspase 3.


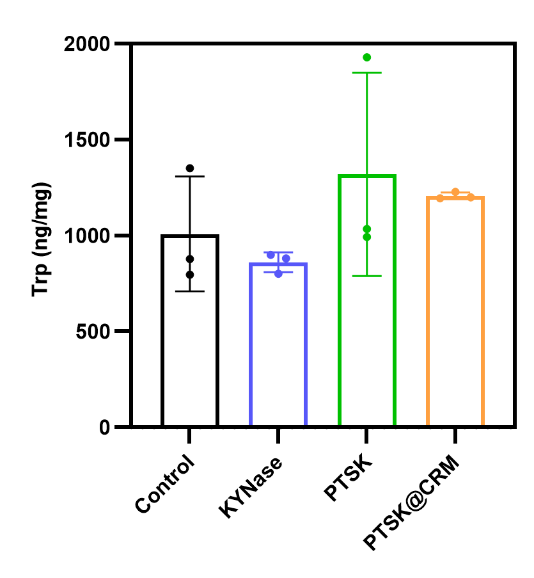


**Figure S18.** The Trp content of CT26 cells after the incubation with different formulations for 24 h. Data were presented as mean value ± SD (n = 3).

**
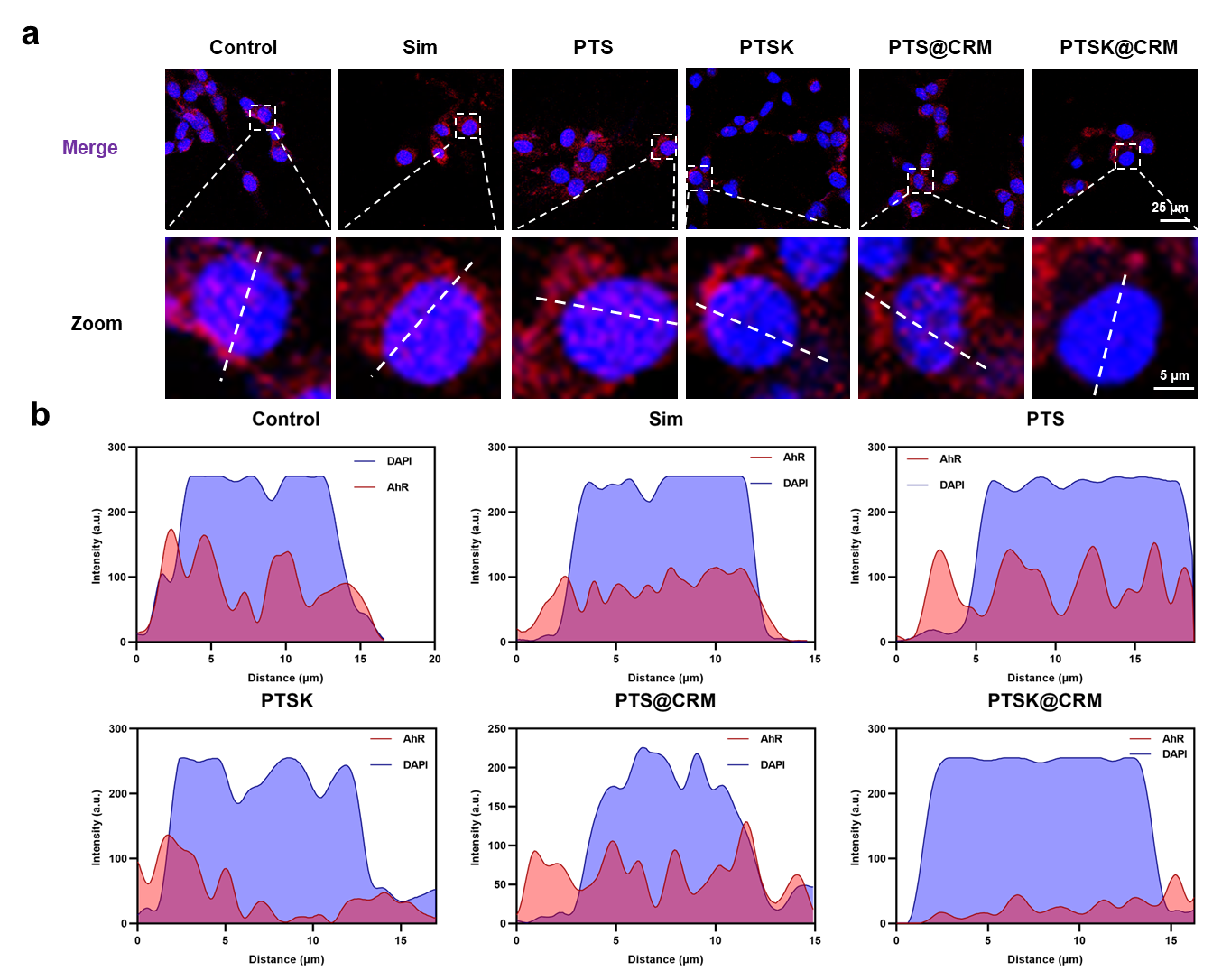
**

**Figure S19. a)** CLSM images and **b)** fluorescence intensity of CT26 cells after different treatments (red: AhR, blue: DAPI-nucleus).


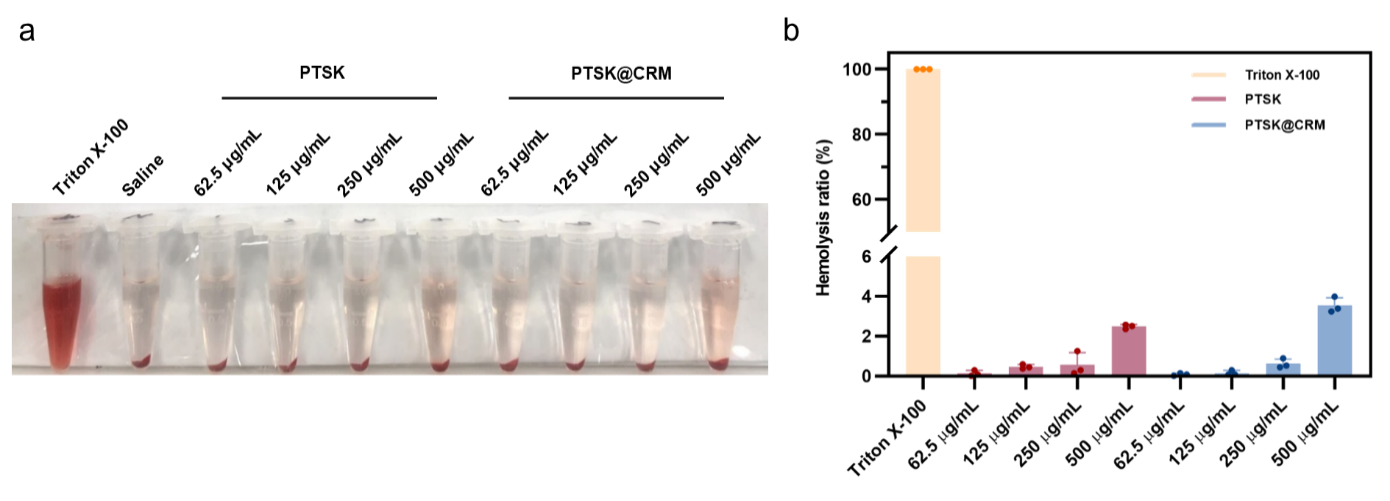


**Figure S20.** **a**) Representative photographs and **b**) relative hemolysis rates of RBCs treated with PTSK or PTSK@CRM. Triton X-100 and saline was used as positive and negative controls, respectively. Data were presented as mean value ± SD (n = 3).


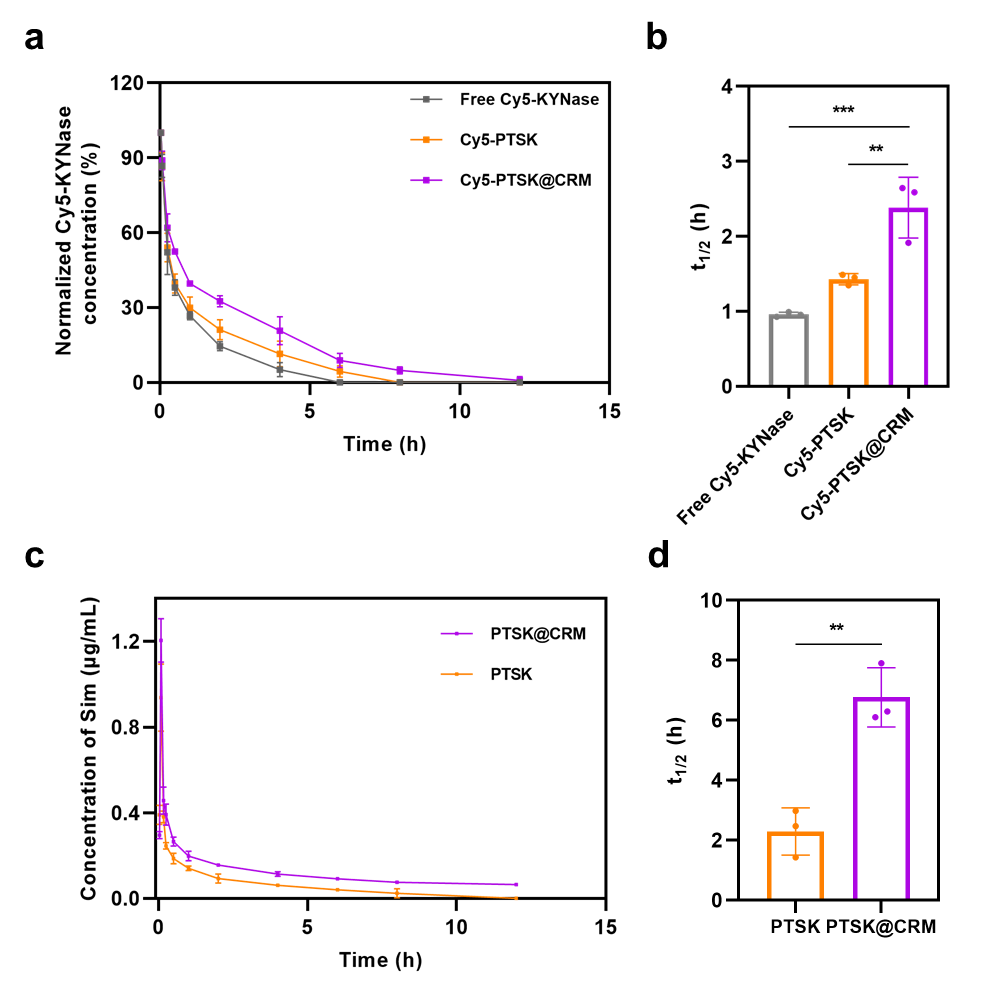


**Figure S21. a)** Plasma concentration-time curve and **b)** the elimination half-life (t_1/2_) of Cy5-KYNase after the intravenous injection of Cy5-KYNase, Cy5-PTSK and Cy5-PTSK@CRM. **c)** Plasma concentration-time curve and **d)** the elimination t_1/2_ of simvastatin after the intravenous injection of PTSK and PTSK@CRM. Data were presented as mean ± SD (n =3, one-way ANOVA, *******P* < 0.01, ********P* < 0.001).


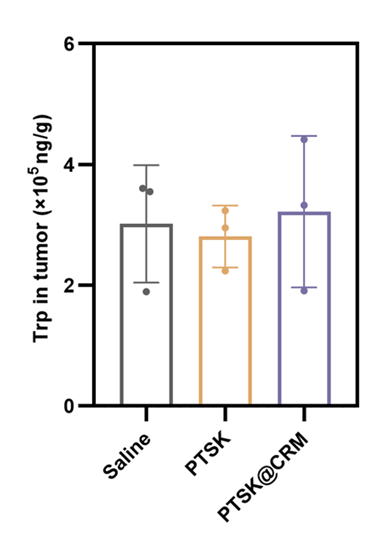


**Figure S22.** Trp content in CT26 tumor tissues of saline, PTSK and PTSK@CRM groups on Day 14. Data were presented as mean value ± SD (n = 3).


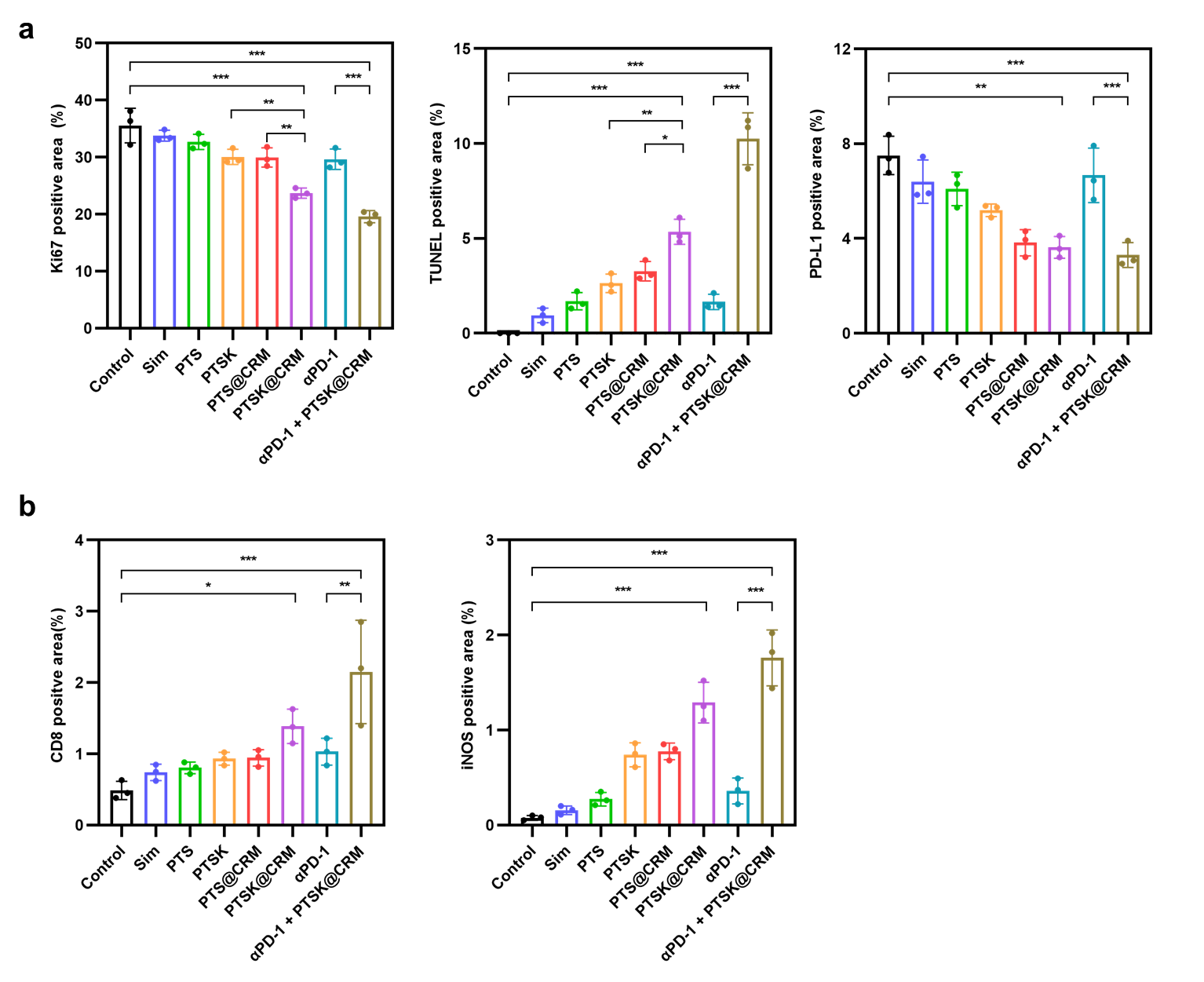


**Figure S23.** Quantitative analysis of pathological staining of **a)** Ki67 IHC, TUNEL and PD-L1 IF, and **b)** CD8 and iNOS IF of tumors. Data were presented as mean value ± SD (n =3). Statistical significance was calculated *via* one-way ANOVA analysis (******P* < 0.05, *******P* < 0.01, ********P* < 0.001).


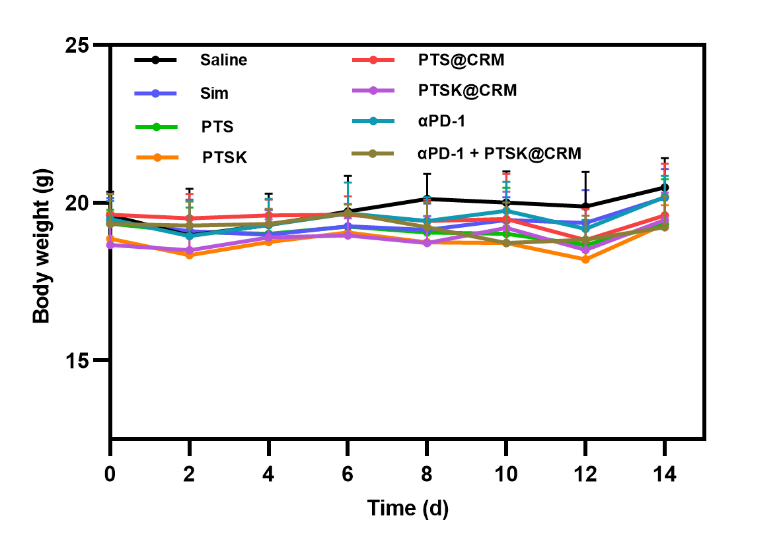


**Figure S24.** Body weight curves of CT26 subcutaneous tumors-bearing mice after different treatments. Data were presented as mean value ± SD (n = 5).


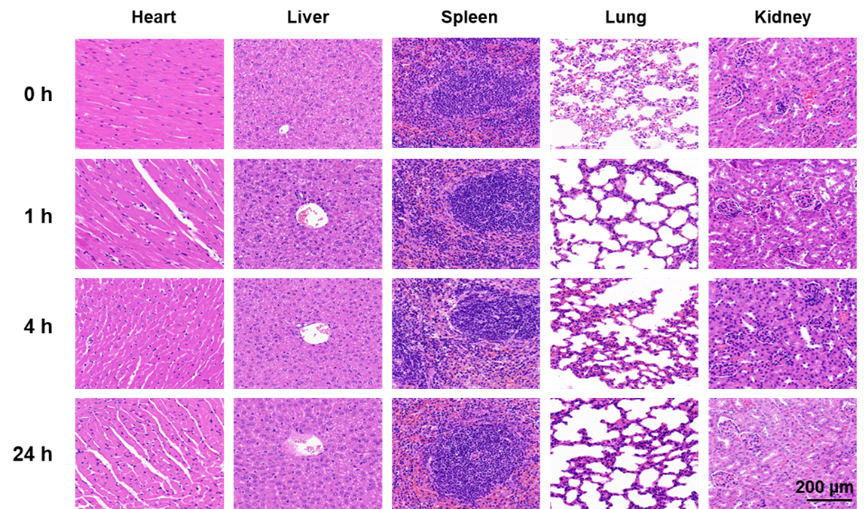


**Figure S25.** H&E staining of major organs (heart, liver, spleen, lungs, kidneys) from CT26 tumor-bearing mice receiving the PTSK@CRM treatment at 0 h, 1 h, 4 h and 24 h. scale bar = 200 μm.


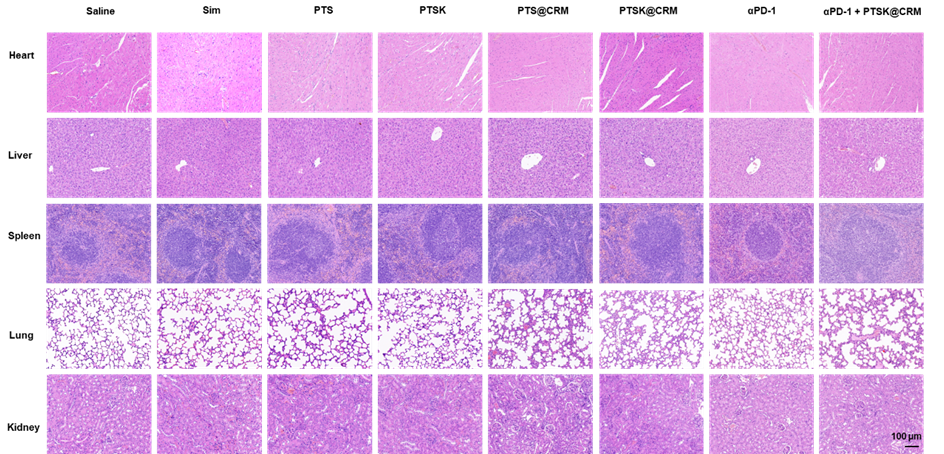


**Figure S26.** H&E staining of major organs (heart, liver, spleen, lungs, kidneys) harvested from the CT26 tumor-bearing mice after different treatments on Day 14. scale bar = 100 μm.


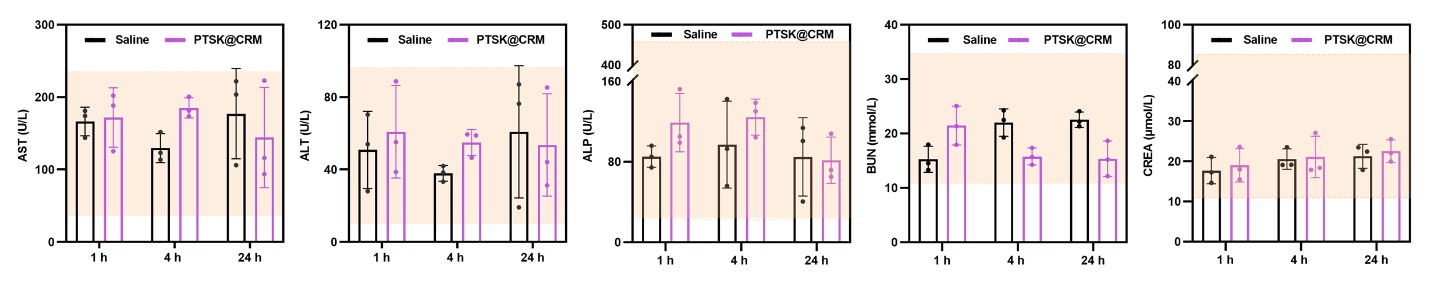


**Figure S27.** Serum biochemistry analysis of CT26 tumor-bearing mice after the treatment with PTSK@CRM at 1 h, 4 h or 24 h. Data were presented as mean value ± SD (n = 3).


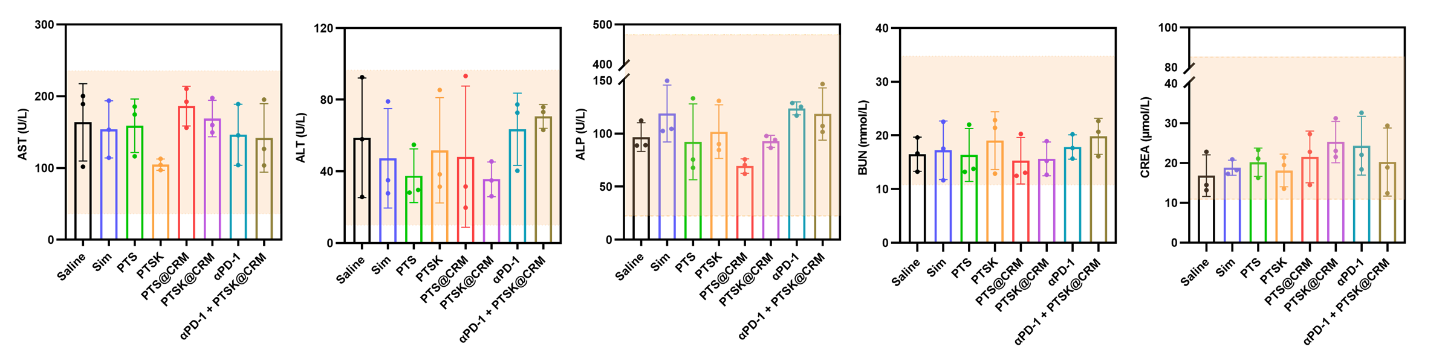


**Figure S28.** Serum biochemistry analysis of CT26 tumor-bearing mice after different treatments on Day 14. Data were presented as mean value ± SD (n = 3).


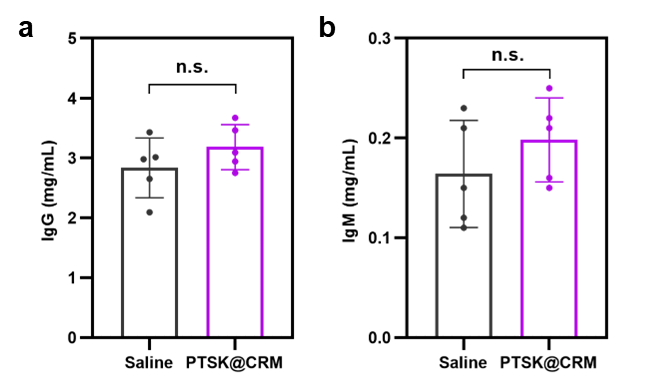


**Figure S29.** Concentration of a) IgG and b) IgM antibodies in the mice after the intravenous injection of PTSK@CRM for 4 times. Data were presented as mean value ± SD (n=5, unpaired and two-tailed t test, n.s. means no significance).


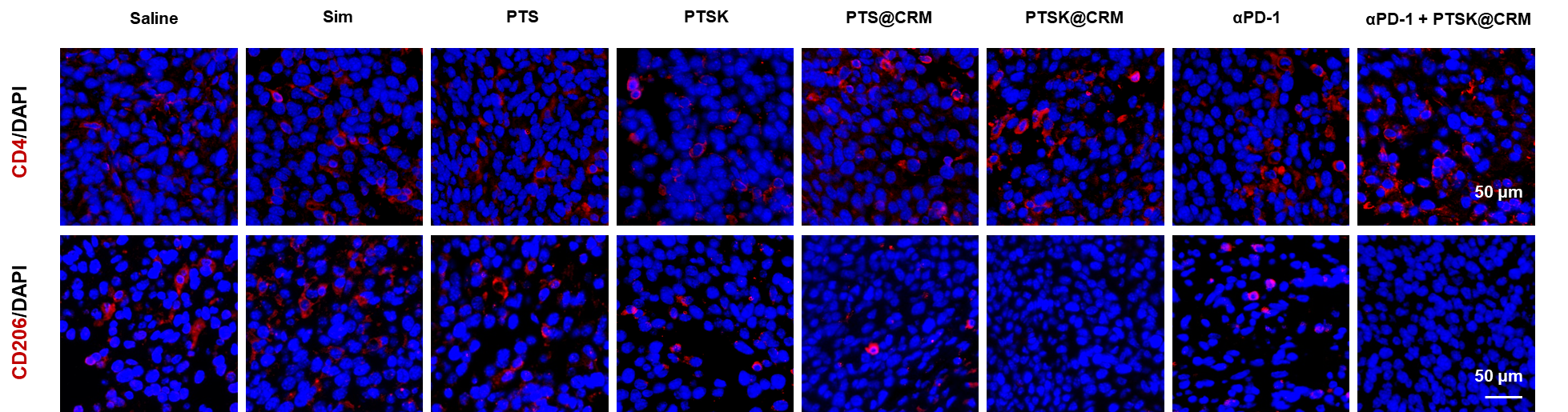


**Figure S30.** IF images for CD4 and CD206 staining in CT26 tumors after different treatments. Blue color represented the nucleus, and red color represented CD4 or CD206. scale bar = 50 μm.


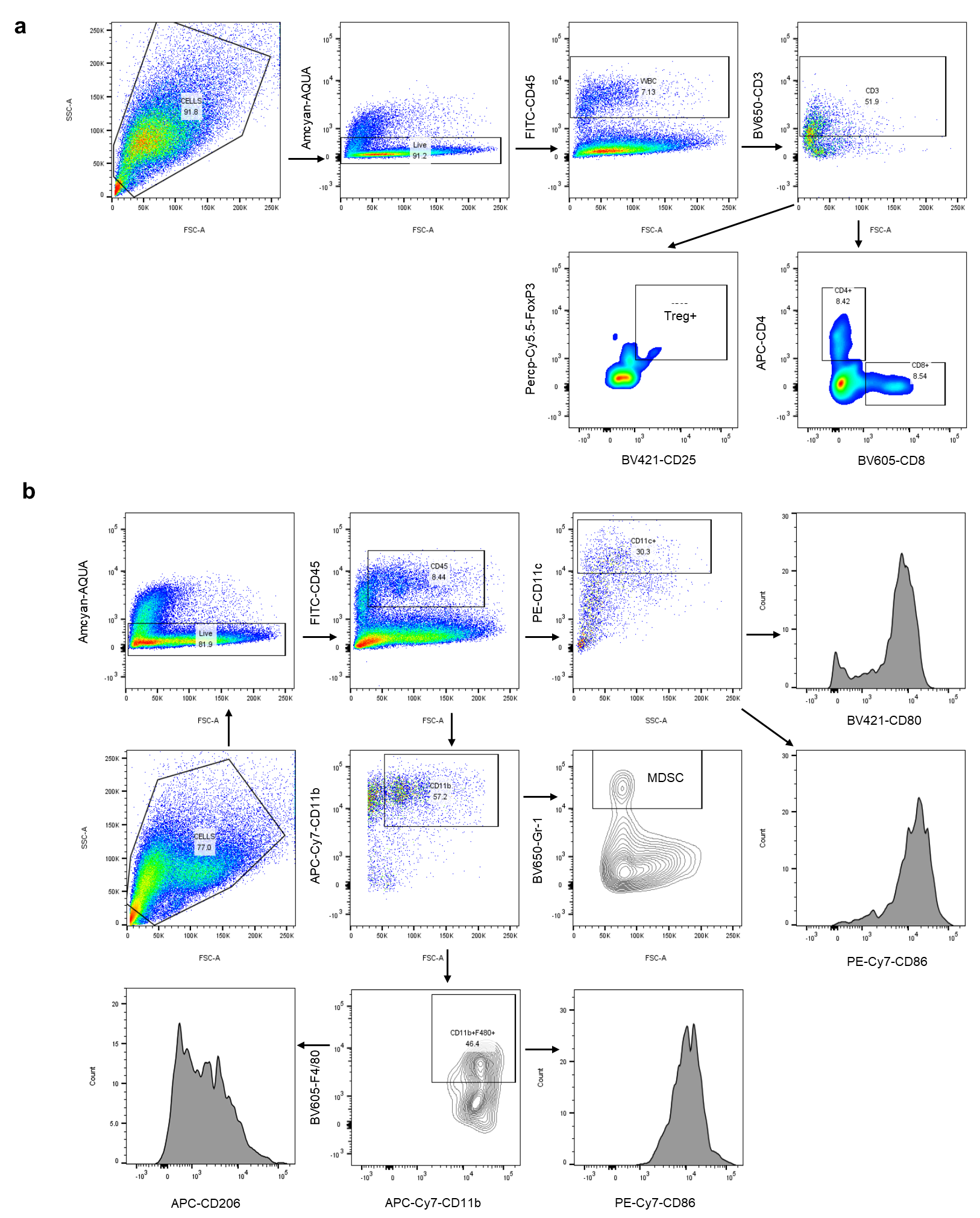


**Figure S31.** Gating strategy for **a**) CD8^+^ T cells, CD4^+^ T cells, Tregs and **b**) activated DCs, M1, M2-like macrophage and MDSCs in tumors.


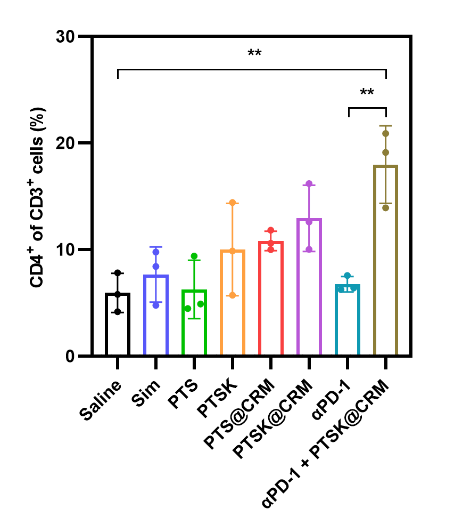


**Figure S32.** Quantitative analysis for the percentage of CD4^+^ T cells in CT26 tumors after different treatments. Data were presented as mean value ± SD (n = 3, one-way ANOVA analysis**, *****P* < 0.01).


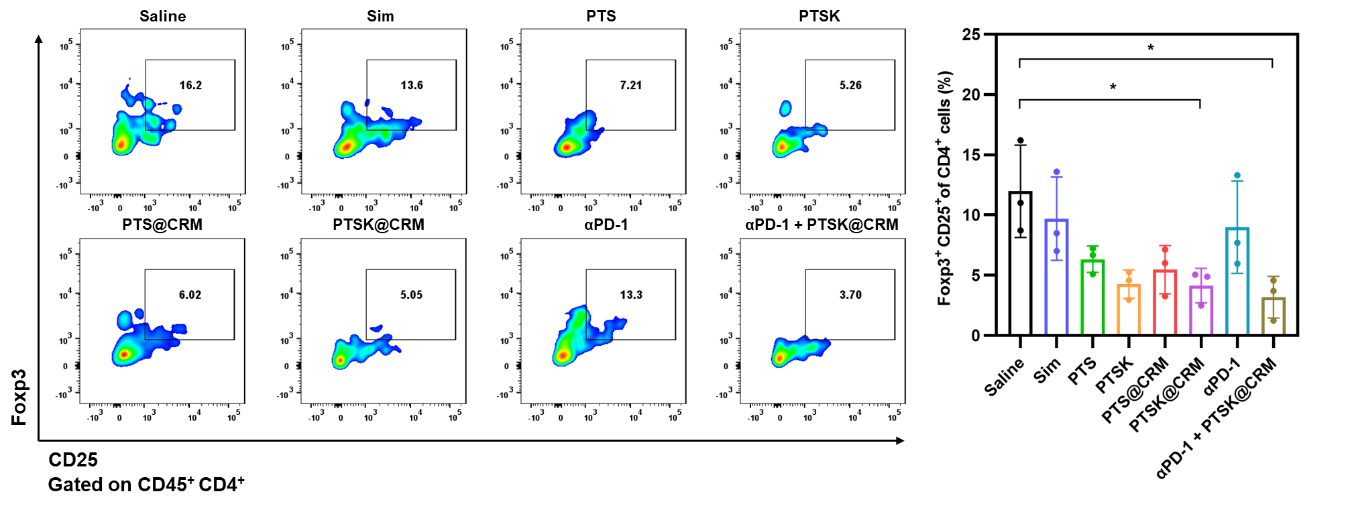


**Figure S33.** Representative flow cytometric plots and quantitative analysis of the percentage of Tregs in CT26 tumors after different treatments. Data were presented as mean value ± SD (n = 3, one-way ANOVA analysis, **P* < 0.05).


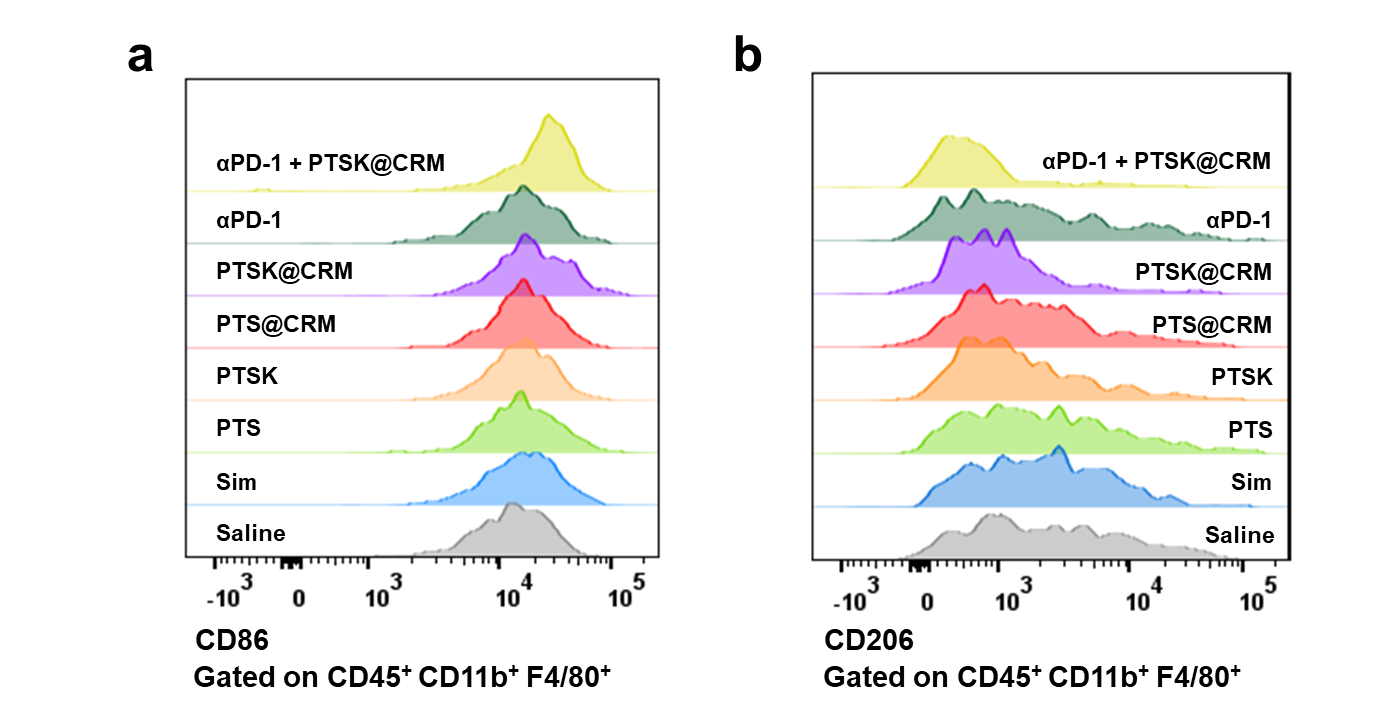


**Figure S34.** Representative flow cytometric histogram of **a**) M1-like and **b**) M2-like macrophage in CT26 tumors after different treatments.


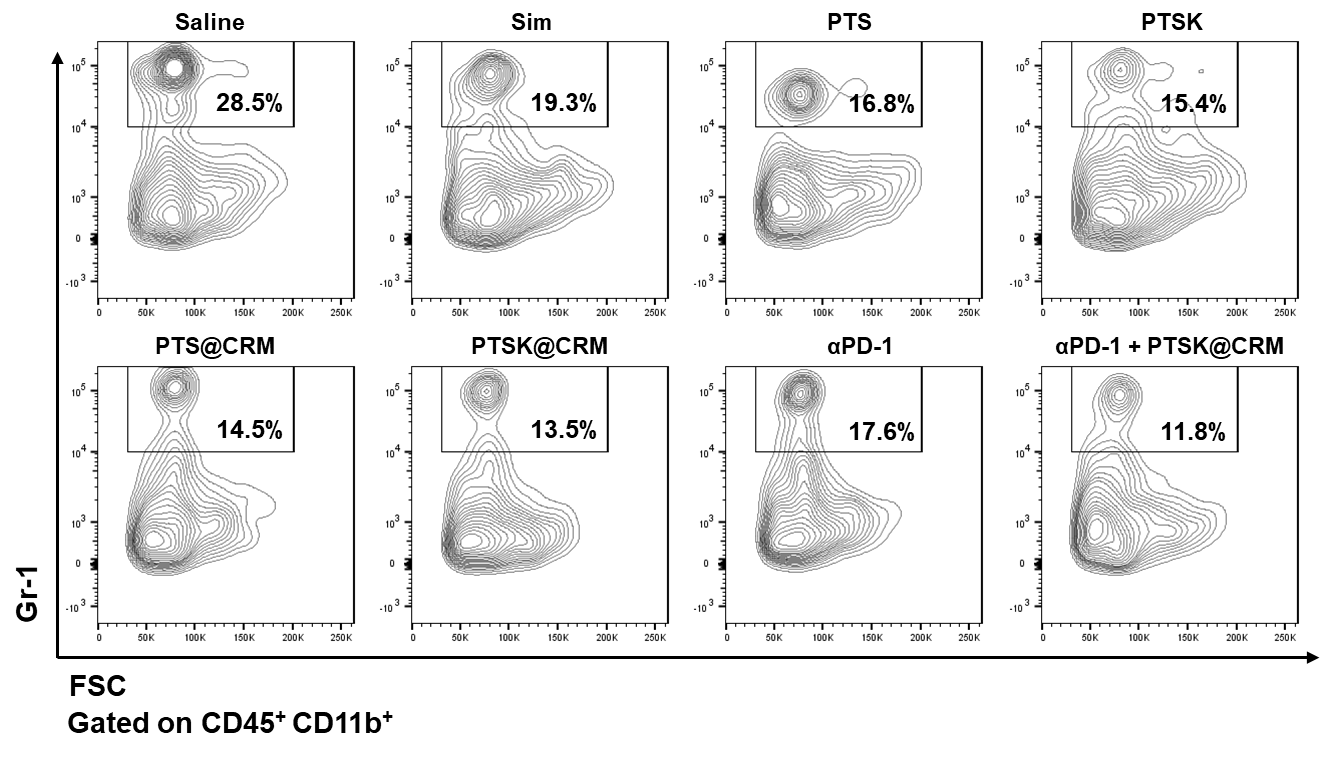


**Figure S35.** Representative flow cytometric plots for the percentage of MDSCs in CT26 tumors after different treatments.


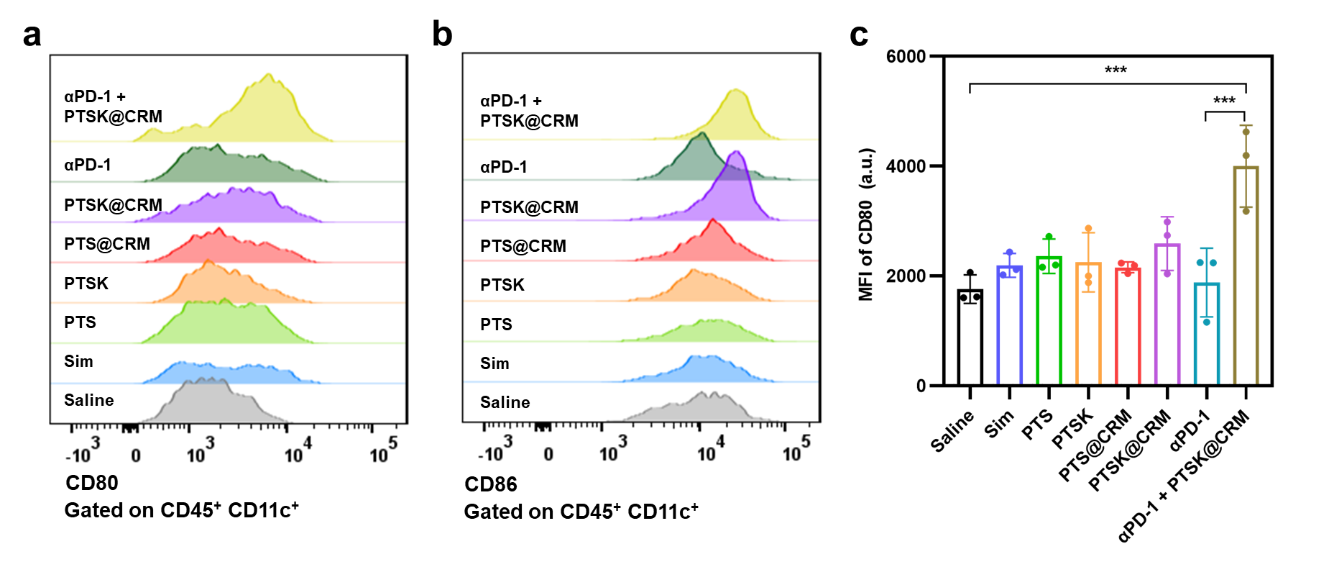


**Figure S36.** Representative flow cytometry histogram of **a**) CD80^+^ and **b**) CD86^+^ mDCs and **c**) the quantitative analysis of CD80^+^ mDCs in CT26 tumors after different treatments. Data were presented as mean value ± SD (n = 3, one-way ANOVA analysis, ****P* < 0.001).


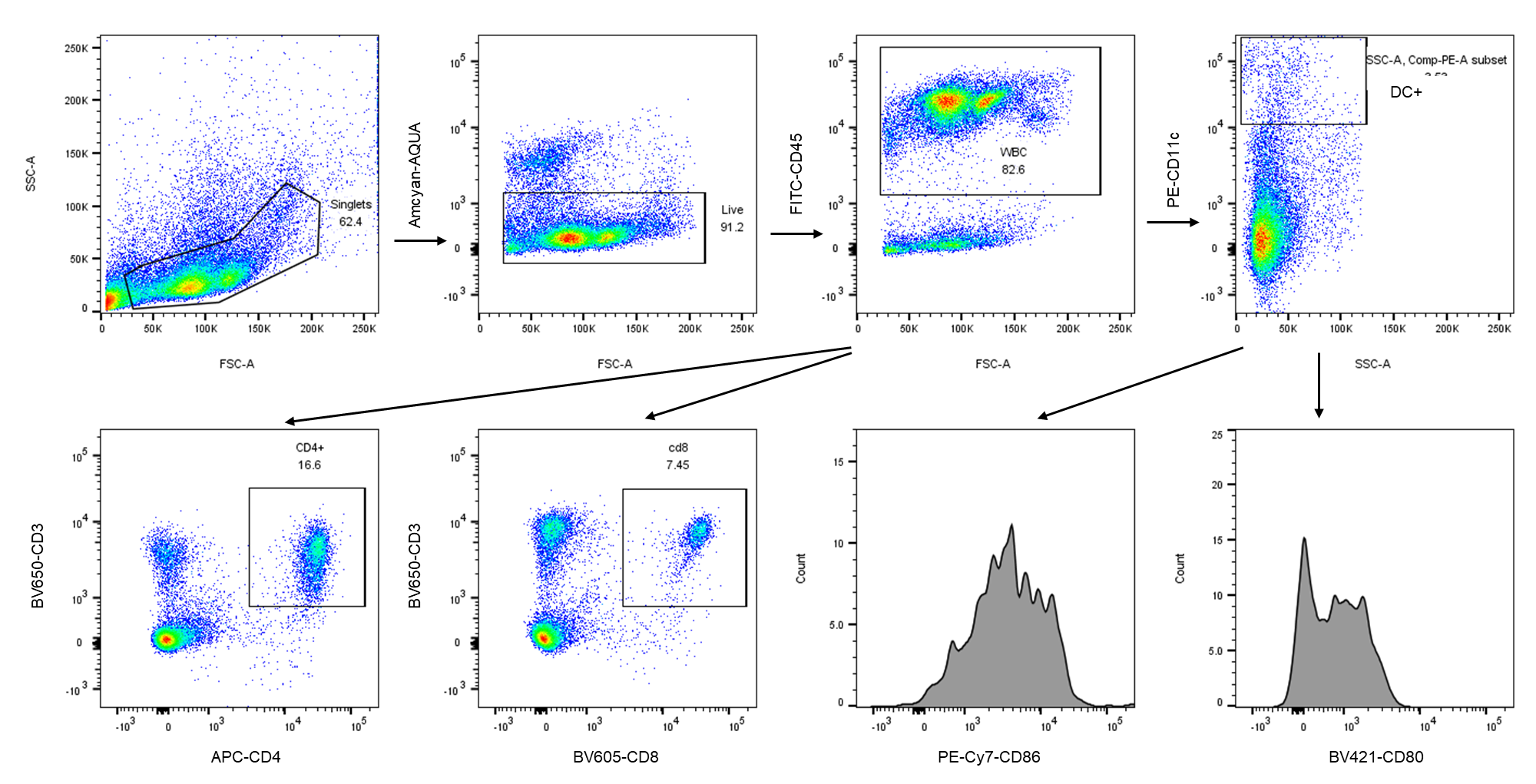


**Figure S37.** Gating strategy for CD8^+^ T cells, CD4^+^ T cells and activated DCs in TDLNs and spleens.


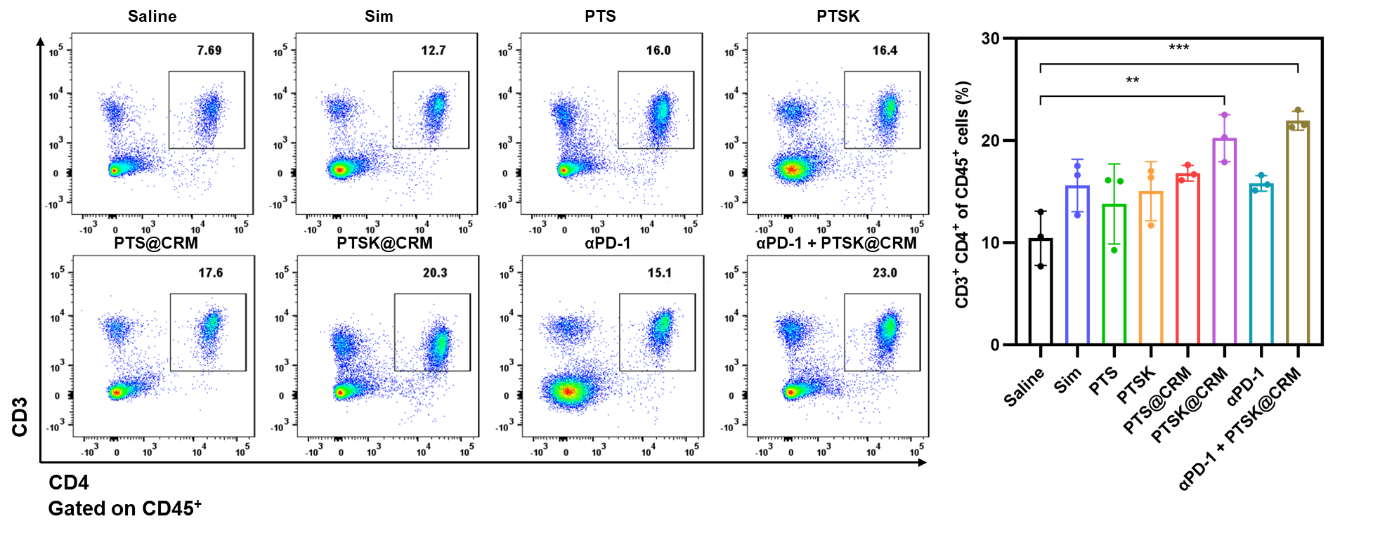


**Figure S38.** Representative flow cytometry plots and quantitative analysis of CD4^+^ T cells in spleens after different treatments. Data were presented as mean value ± SD (n = 3, one-way ANOVA analysis, ***P* < 0.01, ****P* < 0.001).


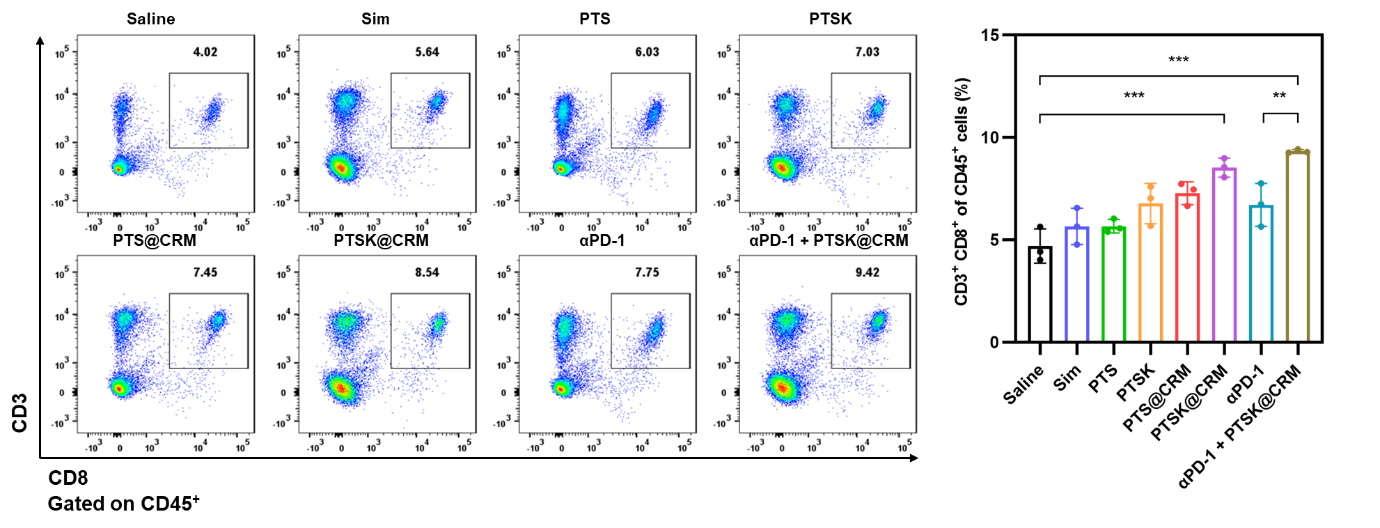


**Figure S39.** Representative flow cytometric plots and the quantitative analysis of CD8^+^ T cells in spleens after different treatments. Data were presented as mean value ± SD (n = 3, one-way ANOVA analysis, ***P* < 0.01, ****P* < 0.001).


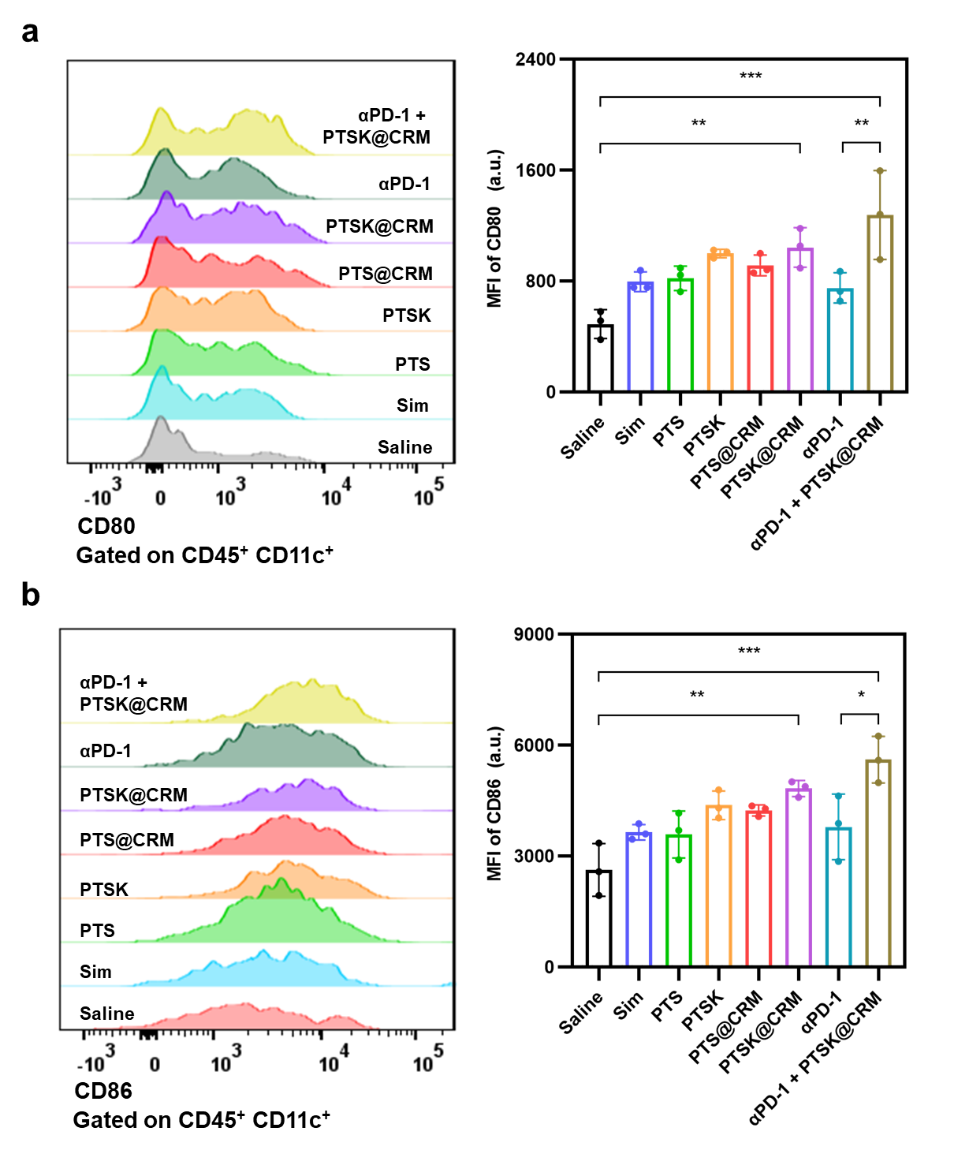


**Figure S40.** Representative flow cytometric histogram and the quantitative analysis of **a)** CD80^+^ and **b)** CD86^+^ mDCs in spleens after different treatments. Data were presented as mean value ± SD (n = 3, one-way ANOVA analysis, **P* < 0.05, ***P* < 0.01, ****P* < 0.001).


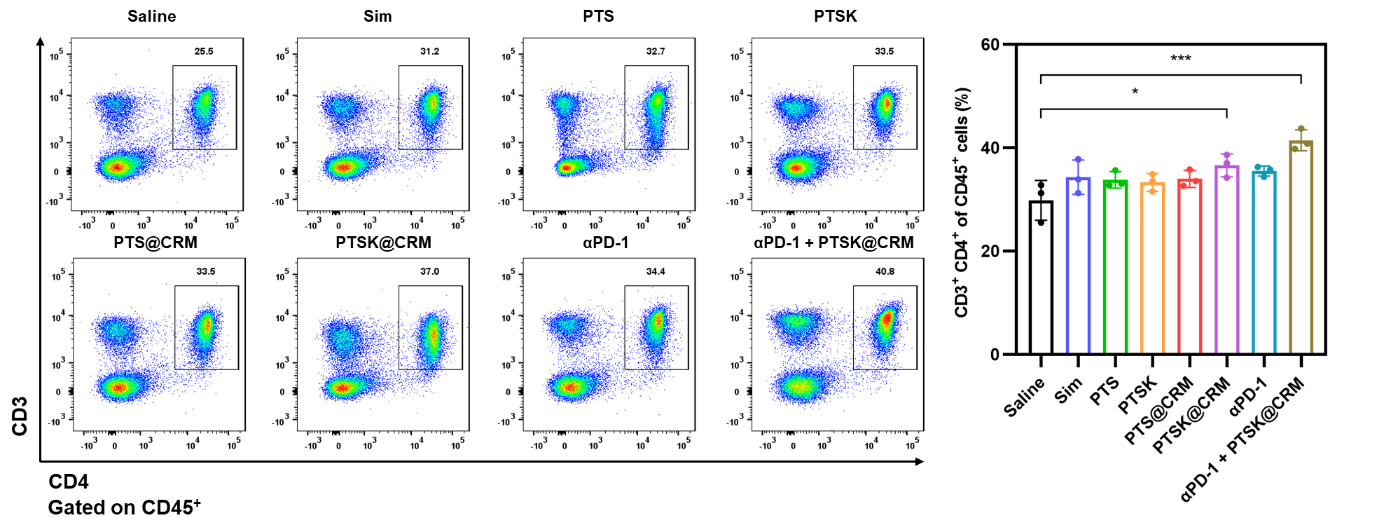


**Figure S41.** Representative flow cytometryic plots and the quantitative analysis of CD4^+^ T cells in TDLNs after different treatments. Data were presented as mean value ± SD (n = 3, one-way ANOVA analysis, **P* < 0.05, ****P* < 0.001).


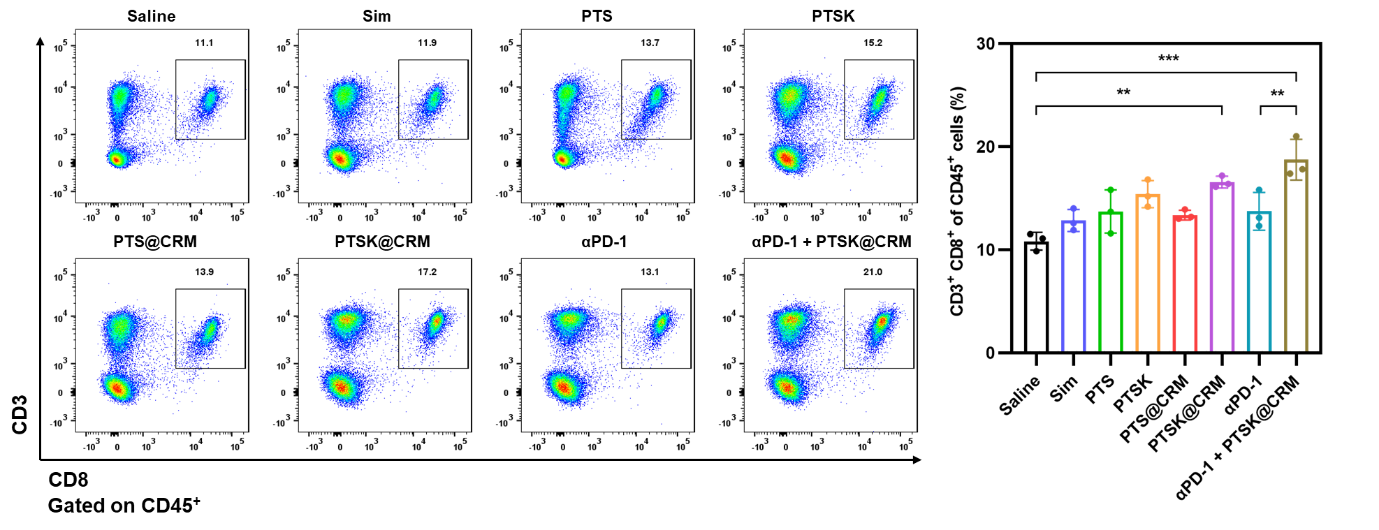


**Figure S42.** Representative flow cytometric plots and the quantitative analysis of CD8^+^ T cells in TDLNs after different treatments. Data were presented as mean value ± SD (n = 3, one-way ANOVA analysis, ***P* < 0.01, ****P* < 0.001).


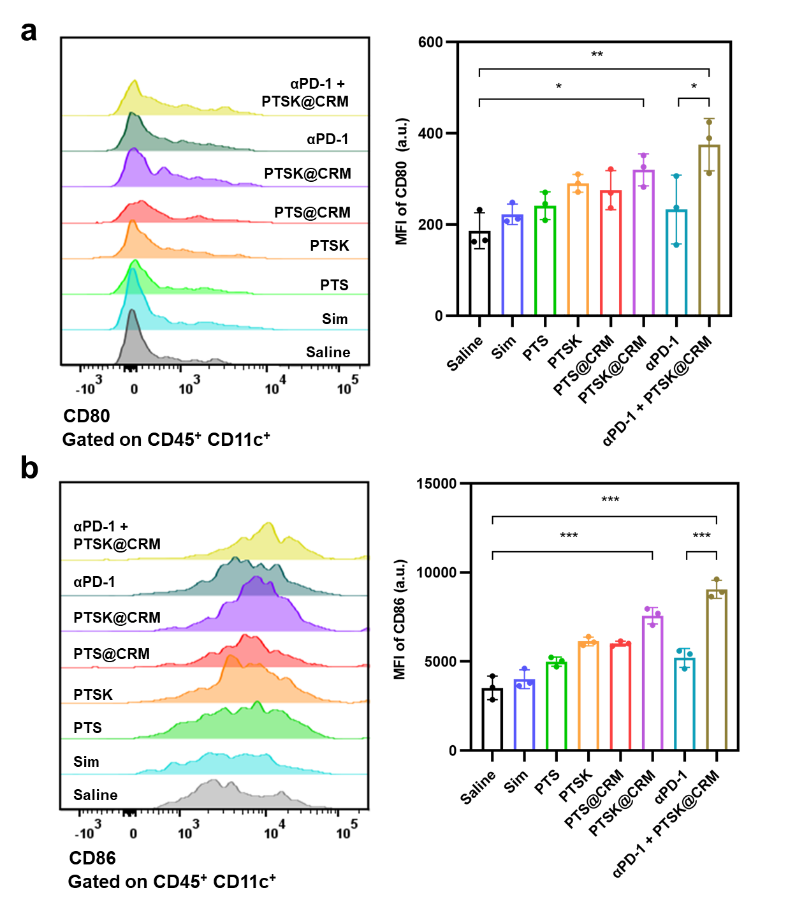


**Figure S43.** Representative flow cytometric histogram and the quantitative analysis of **a**) CD80^+^ and **b**) CD86^+^ mDCs in TDLNs after different treatments. Data were presented as mean value ± SD (n = 3, one-way ANOVA analysis, **P* < 0.05, ***P* < 0.01, ****P* < 0.001).


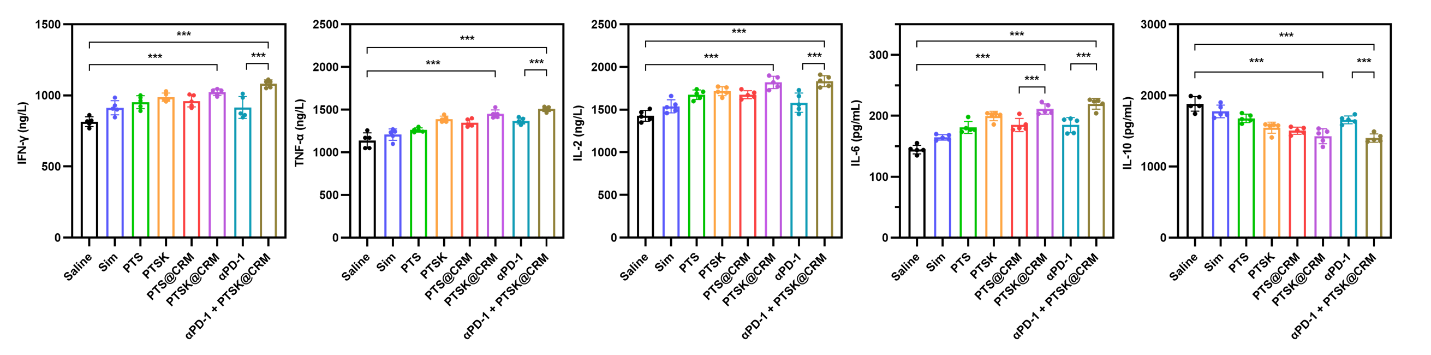


**Figure S44.** The content of IFN-γ, TNF-α, IL-2, IL-6 and IL-10 in serum after different treatments. Data were presented as mean value ± SD (n = 5, one-way ANOVA analysis, ****P* < 0.001).


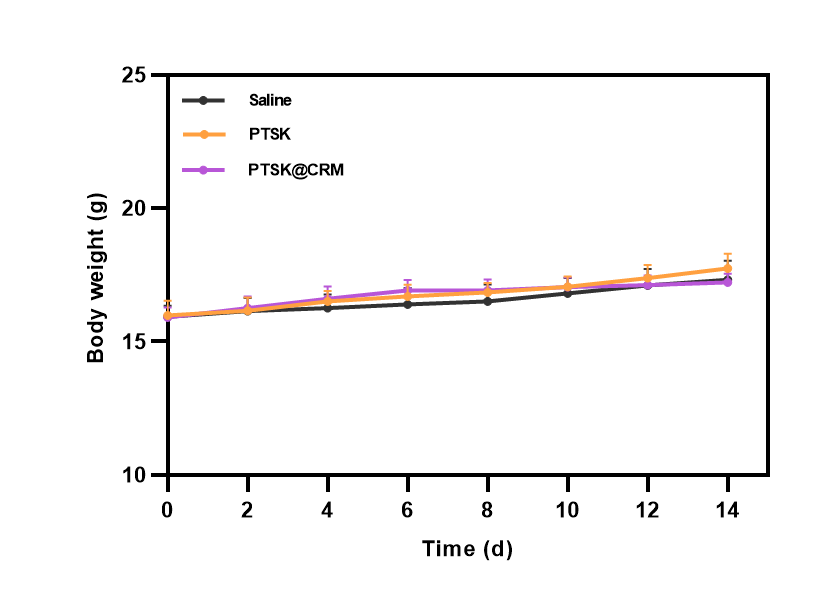


**Figure S45.** Body weight curves of CT26 lung metastasis mice after different treatments. Data were presented as mean value ± SD (n = 5).


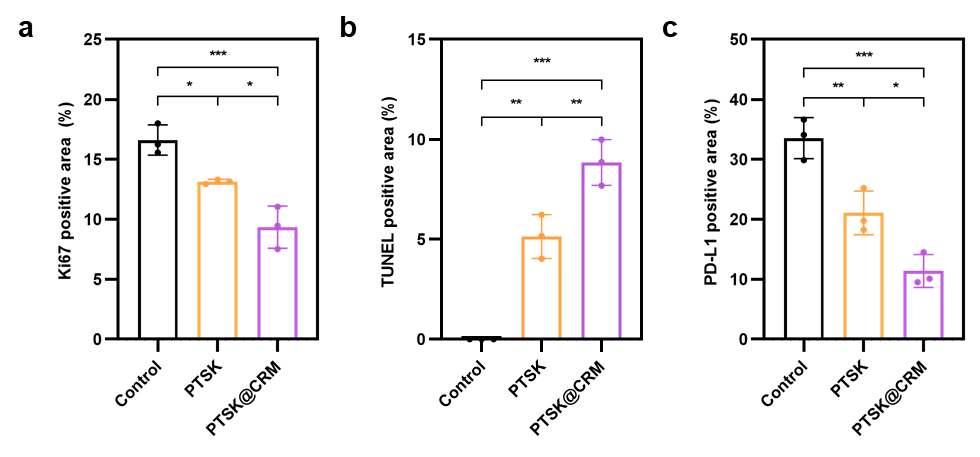


**Figure S46.** Quantitative analysis of pathological staining of **a)** Ki67 IHC, **b)** TUNEL and **c)** PD-L1 IF of lung metastases in CT26 lung metastasis mice. Data were presented as mean value ± SD (n = 3). Statistical significance was calculated *via* one-way ANOVA analysis (******P* < 0.05, *******P* < 0.01, ********P* < 0.001).


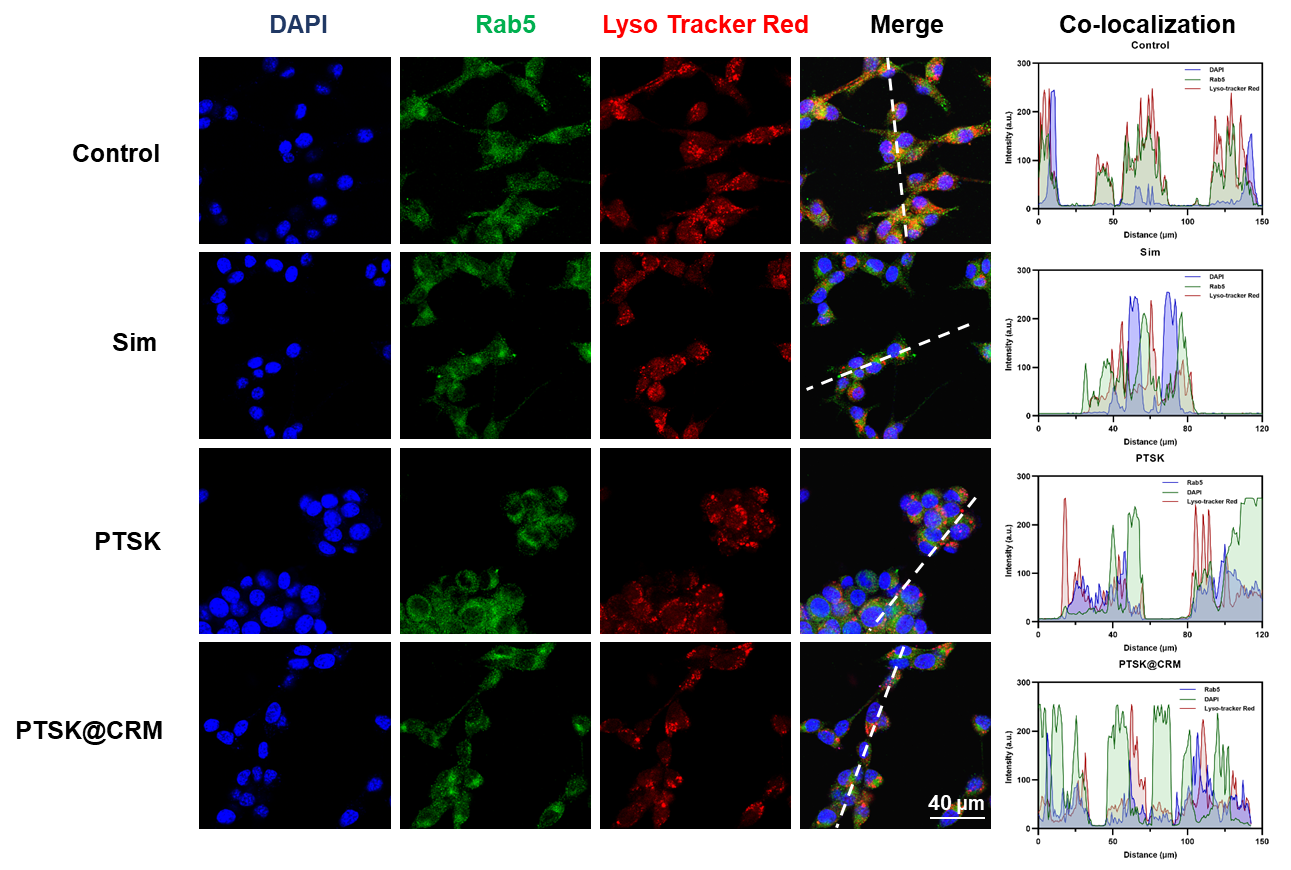


**Figure S47.** CLSM images for the co-location of Rab5 and lysosomes after the treatment of Sim, PTSK or PTSK@CRM (Green: Alexa Fluor^®^488-Rab5, blue: DAPI-nucleus, red: Lyso-Tracker Red, scale bar = 40 μm).


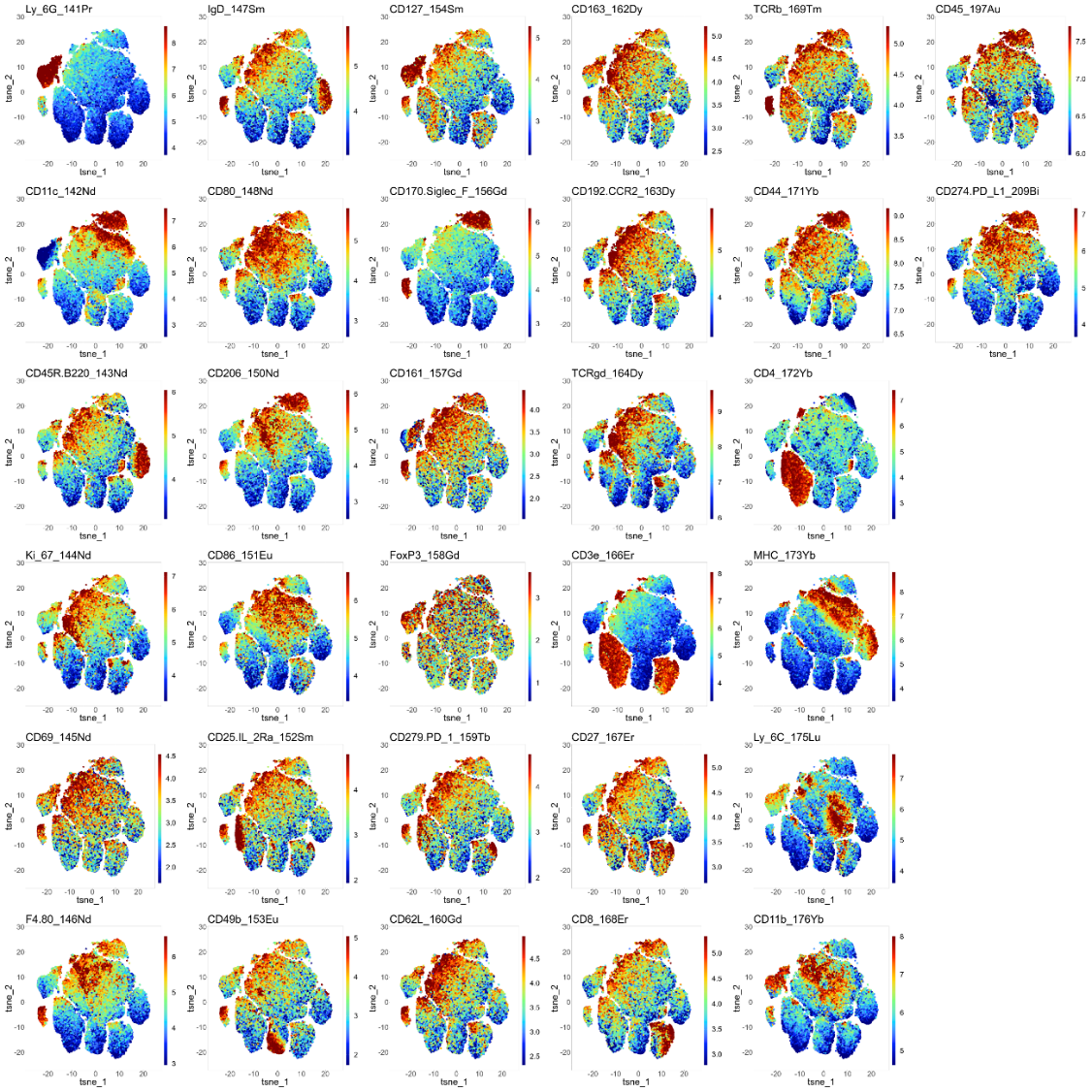


**Figure S48.** t-SNE plots of representative markers in the Saline group detected by CyTOF (n = 3).


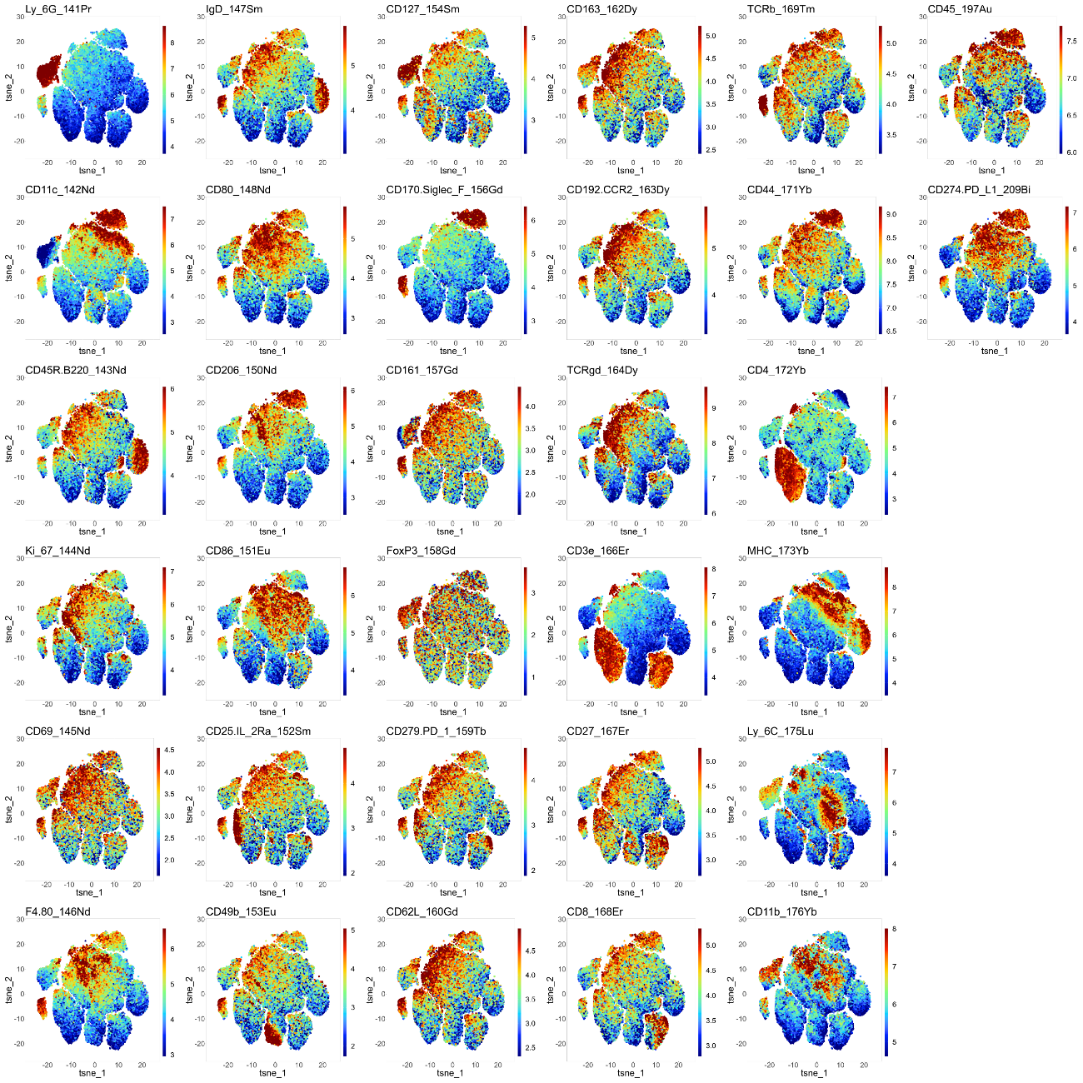


**Figure S49.** t-SNE plots of representative markers in the PTSK@CRM group detected by CyTOF (n = 3).


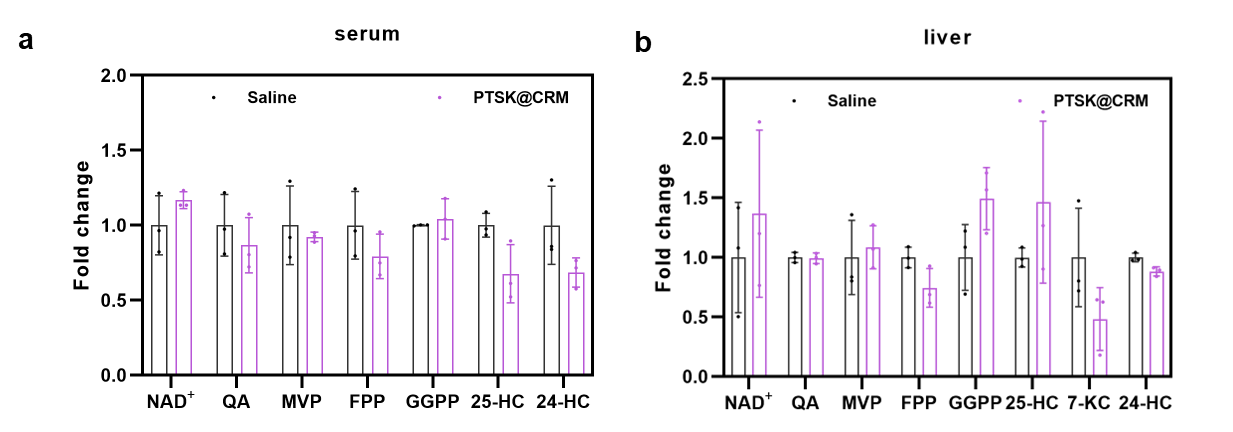


**Figure S50.** The intermediates of Kyn and mevalonate pathways in **a**) serum and **b**) liver tissues. Data were presented as mean value ± SD (n=3). Statistical significance was calculated *via* two-way ANOVA analysis. NAD: nicotinamide adenine dinucleotide, Trp: tryptophane, Kyn: kynurenine, QA: quinolinic acid, MVP: mevalonate-5-phosphate, FPP: farnesyl pyrophosphate, GGPP: geranylgeranyl pyrophosphate, 24-HC: 24-hydroxycholesterol, 25-HC: 25-hydroxycholesterol, 7-KC: 7-ketocholesterol.


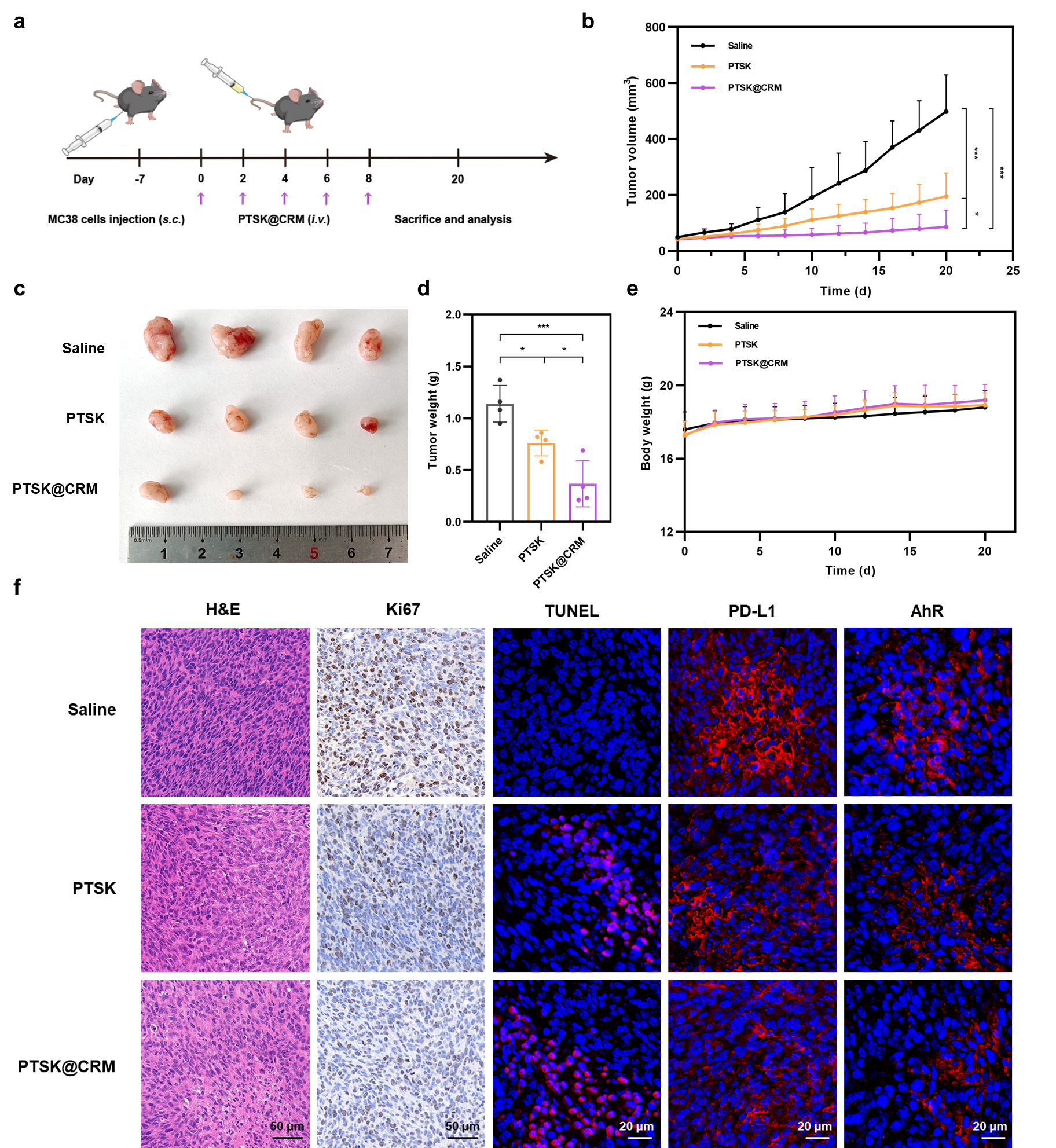


**Figure S51.** *In vivo* anti-tumor efficacy of PTSK@CRM on MC38 tumor-bearing C57BL/6 mice. **a**) Schematic depiction of experimental procedures. **b**) Tumor volume curves of MC38 subcutaneous tumors after different treatments. **c**) Photographs of tumors excised on Day 20 after different treatments. **d**) The mass of excised tumors on Day 20 after different treatments. **e**) Body weight curves of MC38 tumor-bearing mice after different treatments. **f**) Representative pathological staining of tumors on Day 20 (H&E staining, Ki67 IHC, TUNEL, PD-L1 IF and AhR IF). Data were presented as mean value ± SD (n = 4). Statistical significance was calculated *via* one-way ANOVA analysis (******P* < 0.05, ********P* < 0.001).


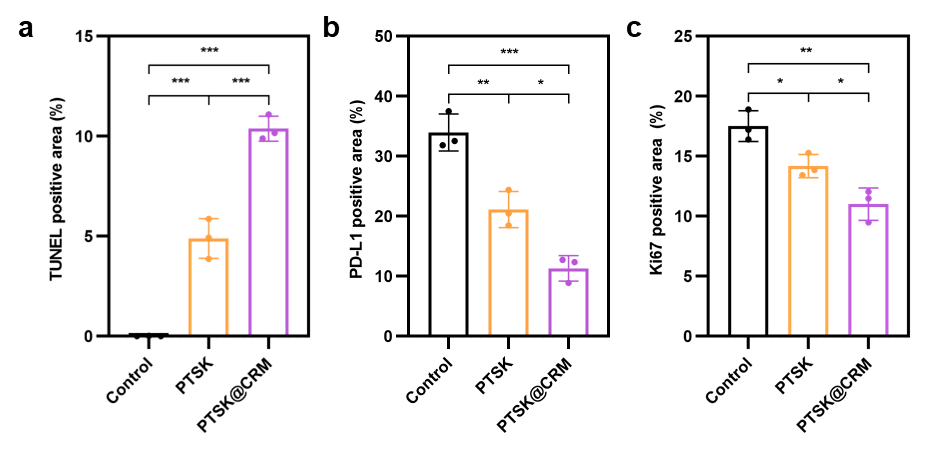


**Figure S52**. Quantitative analysis of pathological staining of **a)** Ki67 IHC, **b)** TUNEL and **c)** PD-L1 IF of MC38 tumors. Data were presented as mean value ± SD (n = 3). Statistical significance was calculated *via* one-way ANOVA analysis (******P* < 0.05, *******P* < 0.01, ********P* < 0.001).


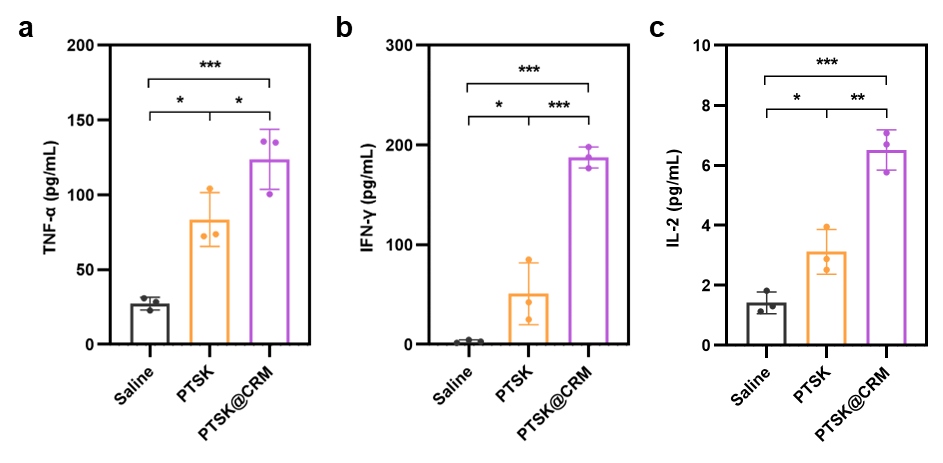


**Figure S53.** The concentrations of IL-2, TNF-α and IFN-γ in the culture supernatants from CT26 lung metastasis model through cytokine recall assay. Data were presented as mean value ± SD (n = 3). Statistical significance was calculated *via* one-way ANOVA analysis (******P* < 0.05, *******P* < 0.01, ********P* < 0.001).


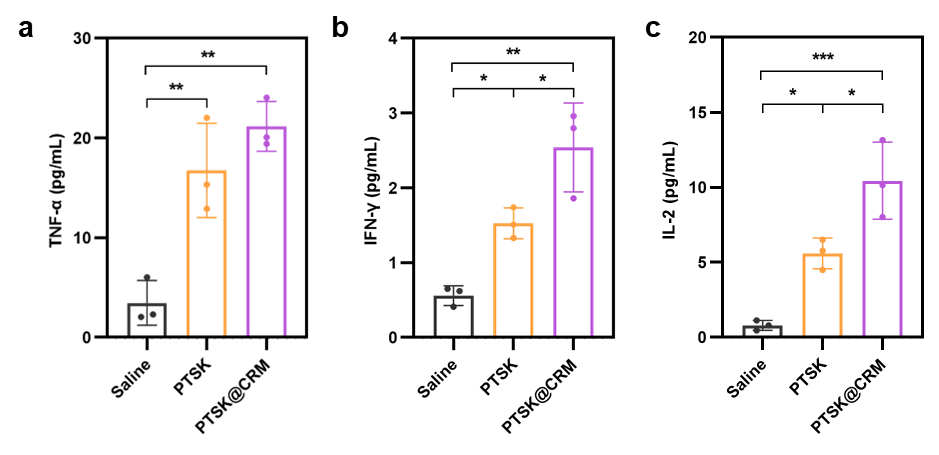


**Figure S54.** The concentrations of IL-2, TNF-α and IFN-γ in the culture supernatants from MC38 tumor model through cytokine recall assay. Data were presented as mean value ± SD (n = 3). Statistical significance was calculated *via* one-way ANOVA analysis (******P* < 0.05, *******P* < 0.01, ********P* < 0.001).

**Table S1.** The characterization of nanoparticles.

|  | PTS | PTSK | PTS@CRM | PTSK@CRM |
| --- | --- | --- | --- | --- |
| EE (%) | 72.8 ± 0.7^a)^ | 83.4 ± 0.8^b)^ | - | - |
| DLC (%) | 10.3 ± 0.5^c)^ | 21.0 ± 1.3^d)^ | - | - |
| Size (d. nm) | 74.1 ± 4.0 | 168.6 ± 7.6 | 131.0 ± 1.7 | 181.3 ± 2.0 |
| Zeta potential (mV) | 15.8 ± 0.5 | 3.11 ± 1.1 | -6.8 ± 0.4 | -10.2 ± 1.6 |
| IC_50_ (μg/mL)^e)^ | 6.4 ± 0.4 | 10.2 ± 0.5 | 6.6 ± 0.4 | 8.0 ± 0.6 |

^a)^ EE of Sim in PTS; ^b)^ EE of KYNase in PTSK; ^c)^ DLC of Sim in PTS; ^d)^ DLC of KYNase in PTSK; ^e)^ the half maximal inhibitory concentration.

**Table S2.** Pharmacokinetic parameters of KYNase.

|  | Free Cy5-KYNase | Cy5-PTSK | Cy5-PTSK@CRM |
| --- | --- | --- | --- |
| AUC_0-t_ (%IDꞏh/mL) | 90.52 ± 5.83 | 122.26 ± 7.98 | 189.72 ± 12.67 |
| AUC_0-inf_ (%IDꞏh/mL) | 99.00 ± 5.50 | 132.56 ± 7.92 | 204.58 ± 15.03 |
| MRT_0-24h_ (h) | 1.43 ± 0.19 | 2.29 ± 0.32 | 3.07 ± 0.32 |
| t_1/2_ (h) | 0.96 ± 0.02 | 1.43 ± 0.06 | 2.38 ± 0.33 |

AUC: area under the curve; t_1/2_: half-life; MRT: mean residence time. Data were presented as mean  ±  SD (n =3, independent experiments).

**Table S3.** Pharmacokinetic parameters of Sim.

|  | PTSK | PTSK@CRM |
| --- | --- | --- |
| AUC_0-t_ (μgꞏh/mL) | 0.79 ± 0.10 | 1.58 ± 0.02 |
| AUC_0-inf_ (μgꞏh/mL) | 0.98 ± 0.15 | 2.15 ± 0.11 |
| MRT_0-24h_ (h) | 3.47 ± 0.80 | 8.90 ± 0.77 |
| t_1/2_ (h) | 2.29 ± 0.64 | 6.77 ± 0.81 |

AUC: area under the curve; t_1/2_: half-life; MRT: mean residence time. Data were presented as mean  ±  SD (n =3, independent experiments).
